# Supplementary figures and images for: Silencing of topical proline hydroxylase domain 2 promotes the healing of rat diabetic wounds by phosphorylating AMPK (part 1 of 2)
Source: PLoS One. 2023 Dec 1;18(12):e0294566. doi: 10.1371/journal.pone.0294566 (PMC10691724; doi:10.1371/journal.pone.0294566)

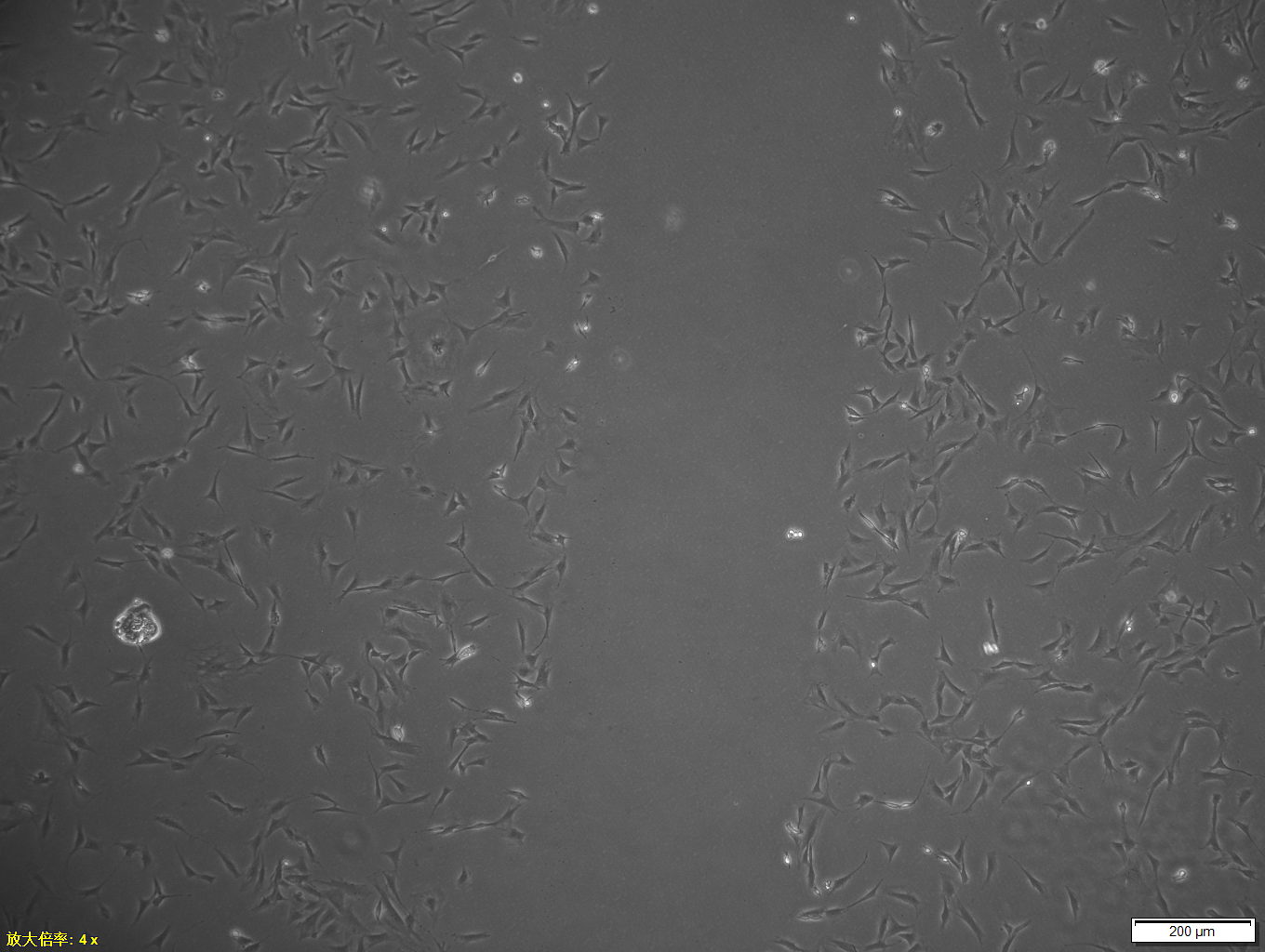

Supplement: S4 File — (ZIP) [file pone.0294566.s004.zip › support information/scrach/0h/sh-Control/scrach-0h-shcontrol-1.tif]

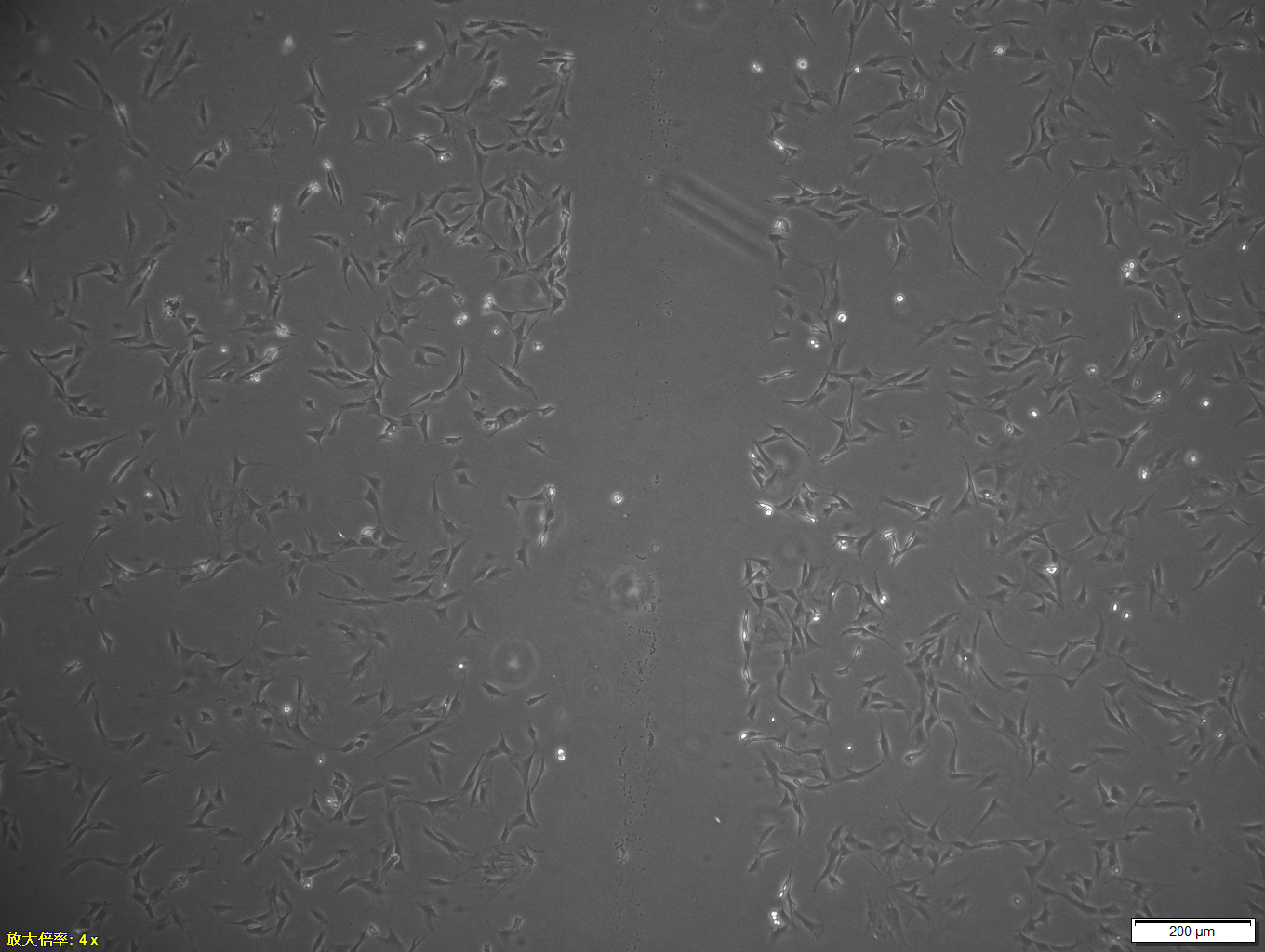

Supplement: S4 File — (ZIP) [file pone.0294566.s004.zip › support information/scrach/0h/sh-Control/scrach-0h-shcontrol-2.tif]

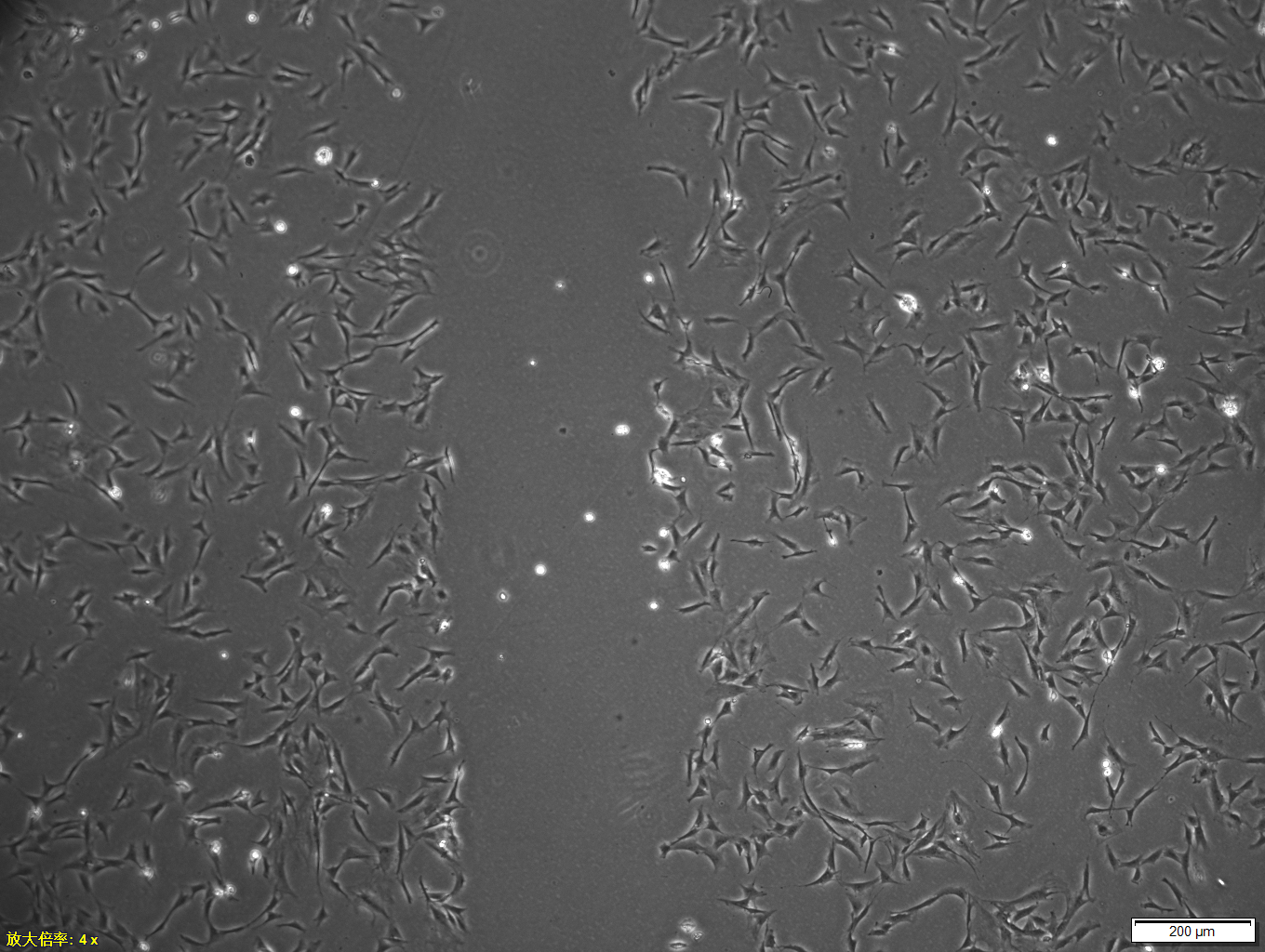

Supplement: S4 File — (ZIP) [file pone.0294566.s004.zip › support information/scrach/0h/sh-PHD2/scrach-0h-shphd2-1.tif]

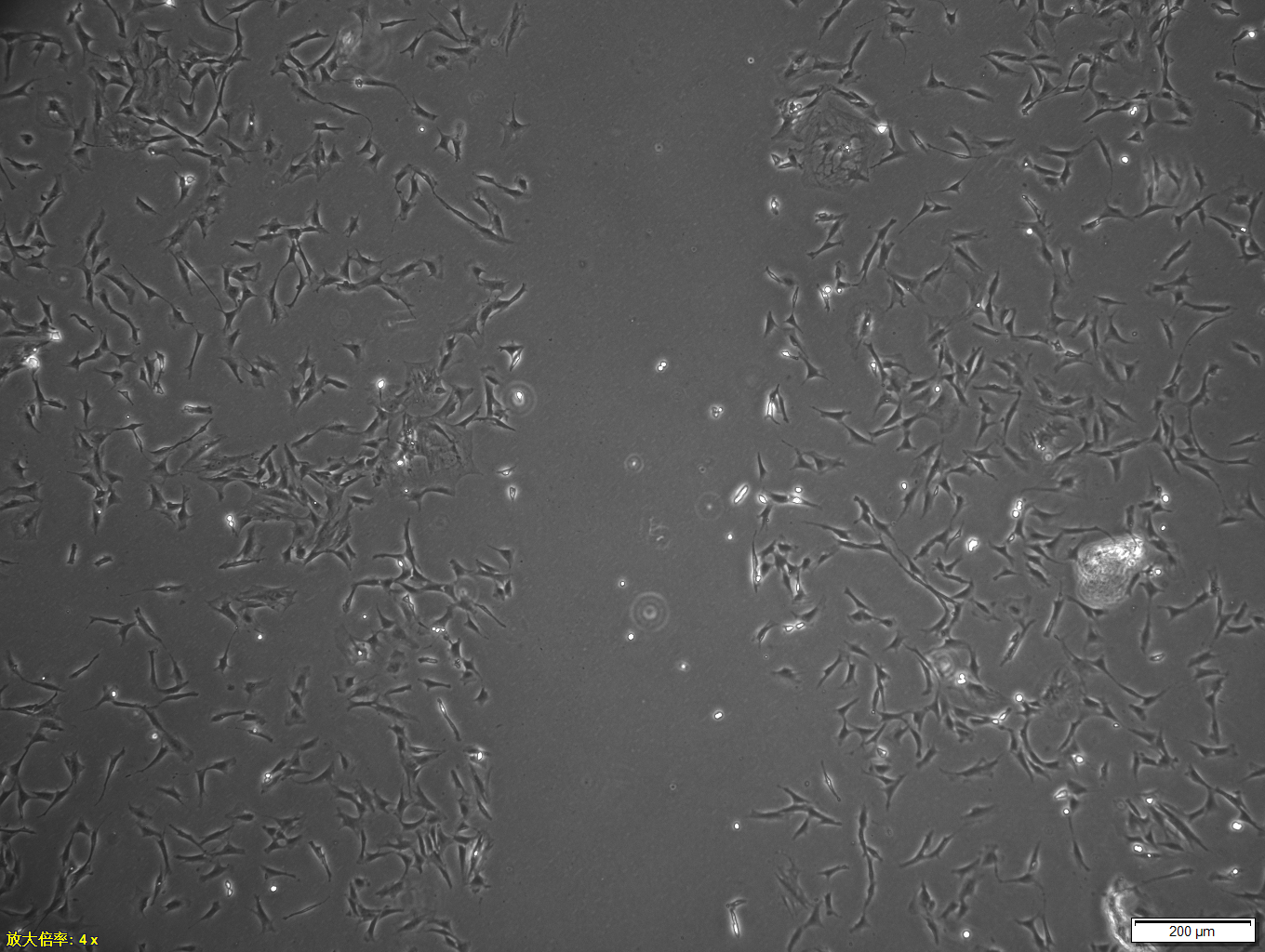

Supplement: S4 File — (ZIP) [file pone.0294566.s004.zip › support information/scrach/0h/sh-PHD2/scrach-0h-shphd2-2.tif]

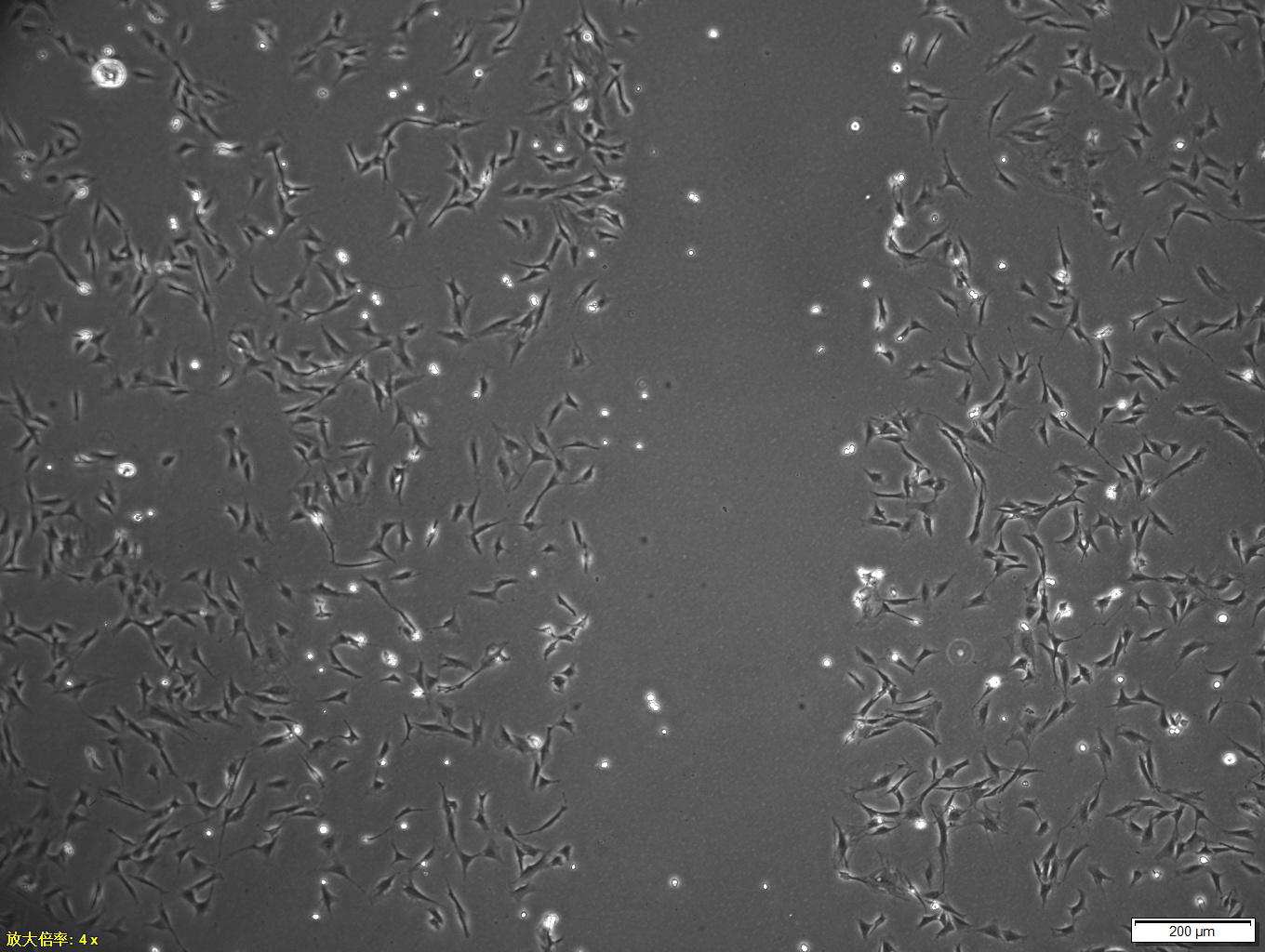

Supplement: S4 File — (ZIP) [file pone.0294566.s004.zip › support information/scrach/0h/sh-PHD2+8um Dorsomorphin/scrach-0h-sh-PHD2+8um Dorsomorphin-1.tif]

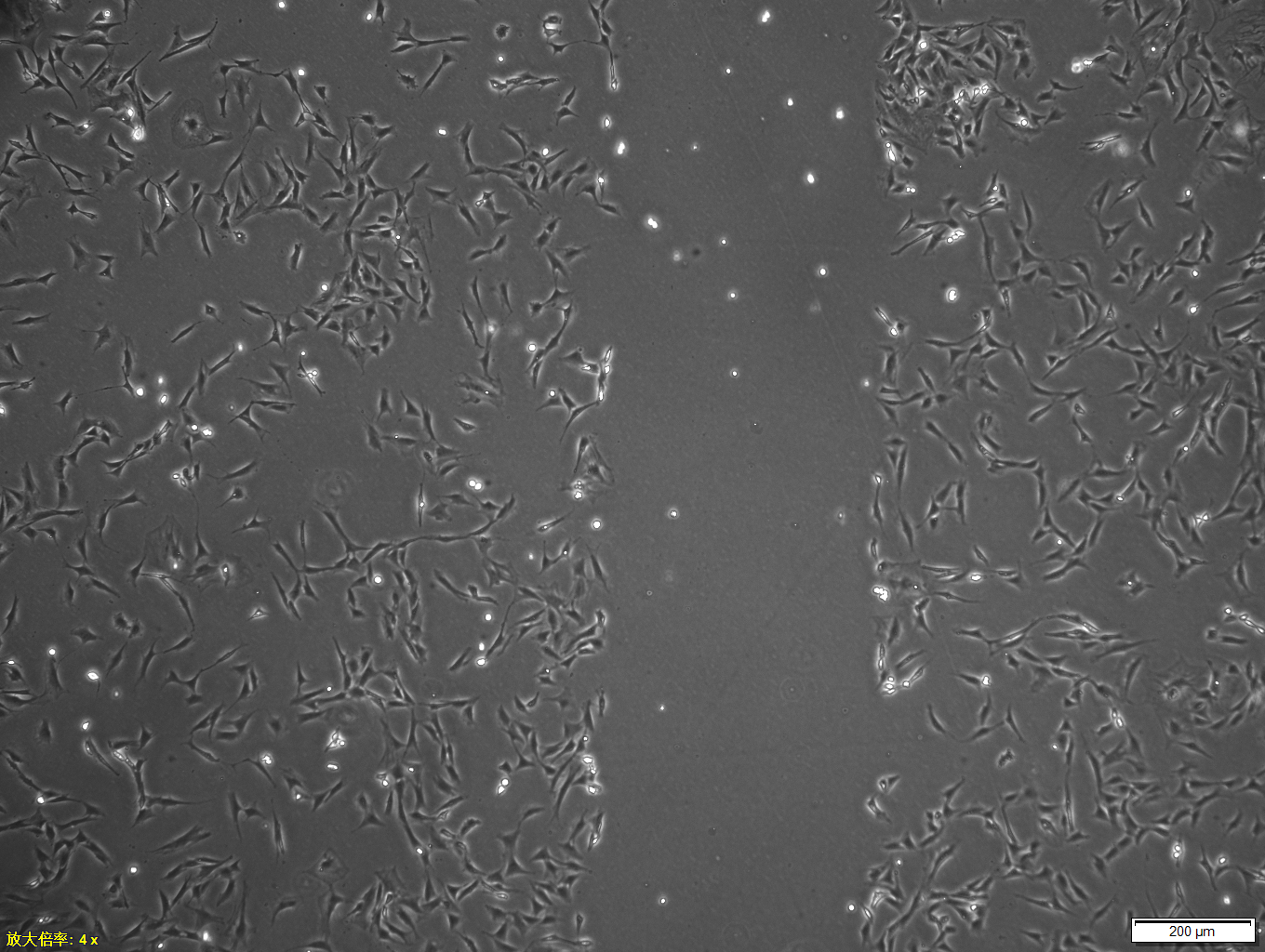

Supplement: S4 File — (ZIP) [file pone.0294566.s004.zip › support information/scrach/0h/sh-PHD2+8um Dorsomorphin/scrach-0h-sh-PHD2+8um Dorsomorphin-2.tif]

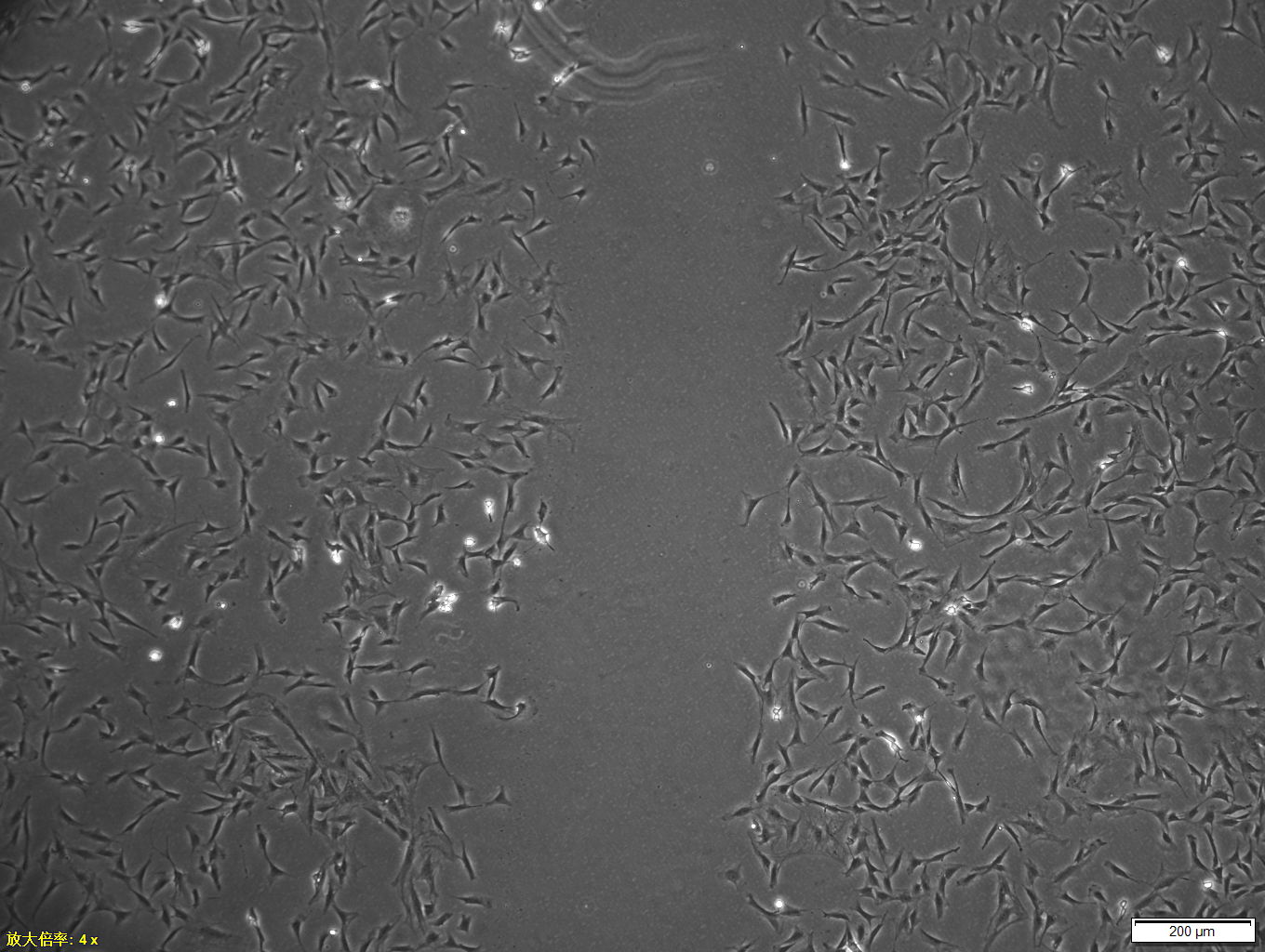

Supplement: S4 File — (ZIP) [file pone.0294566.s004.zip › support information/scrach/12h/sh-Control/scrach-12h-shcontrol-1.tif]

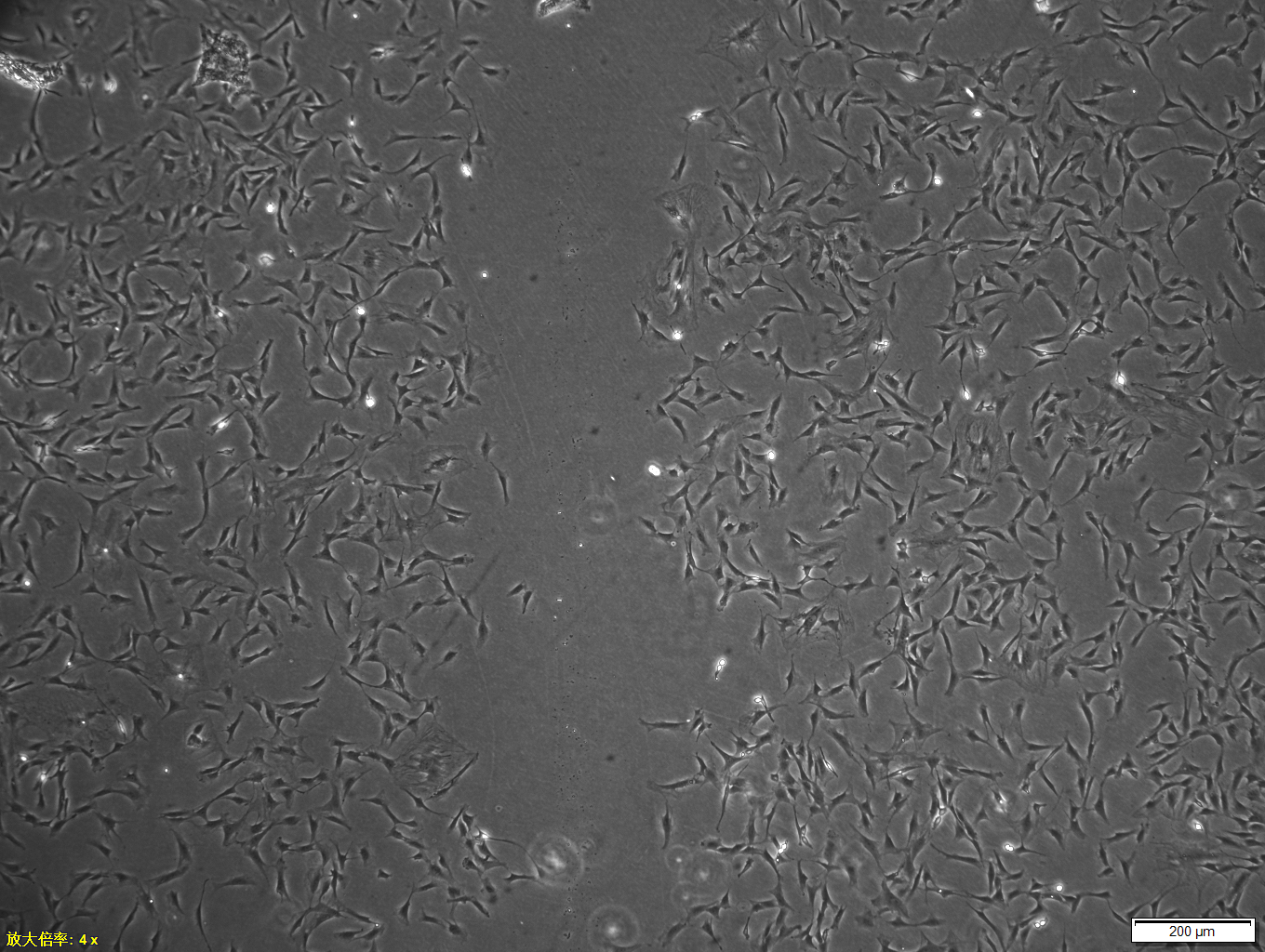

Supplement: S4 File — (ZIP) [file pone.0294566.s004.zip › support information/scrach/12h/sh-Control/scrach-12h-shcontrol-2.tif]

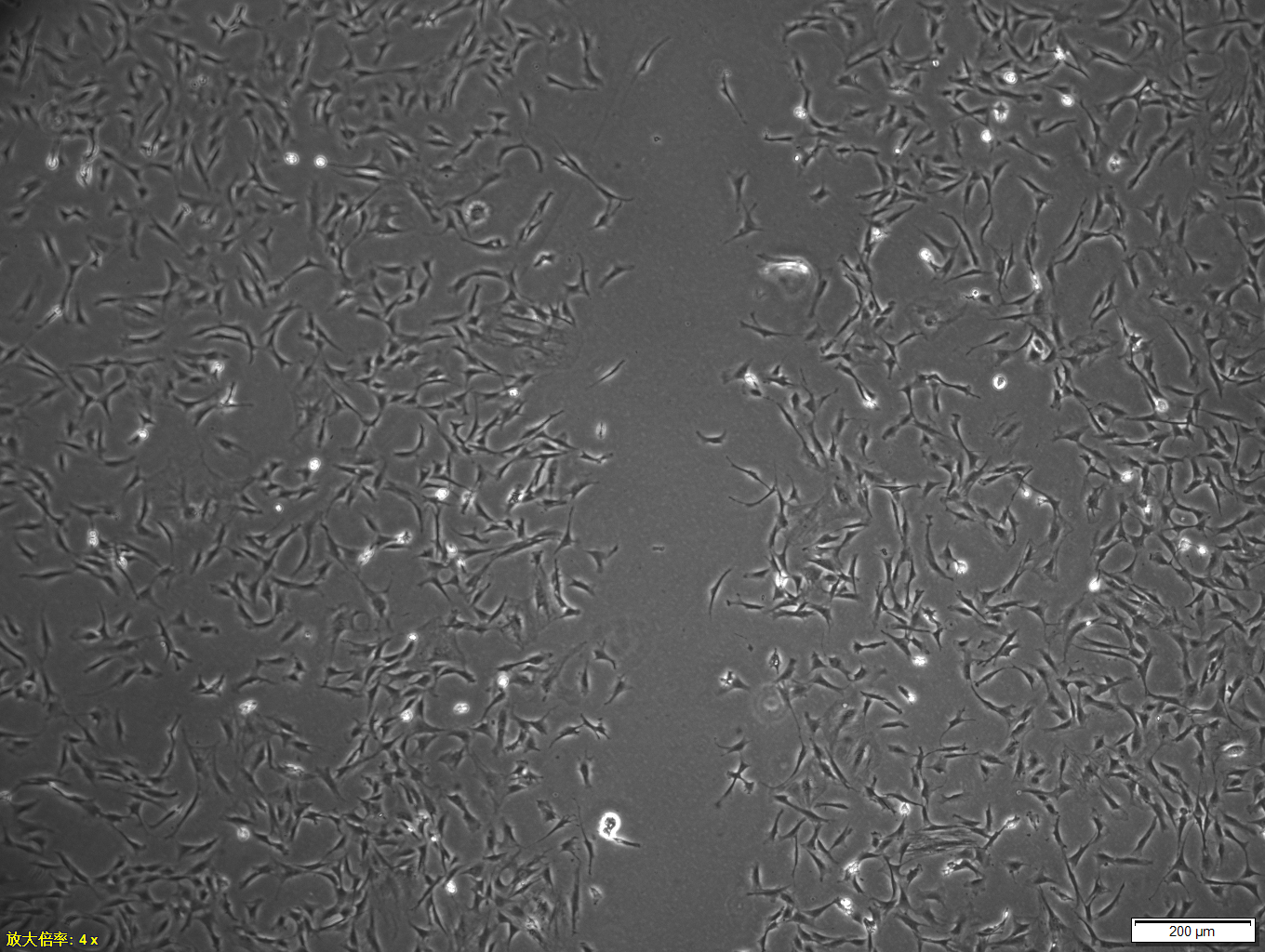

Supplement: S4 File — (ZIP) [file pone.0294566.s004.zip › support information/scrach/12h/sh-PHD2/scrach-12h-shphd2-1.tif]

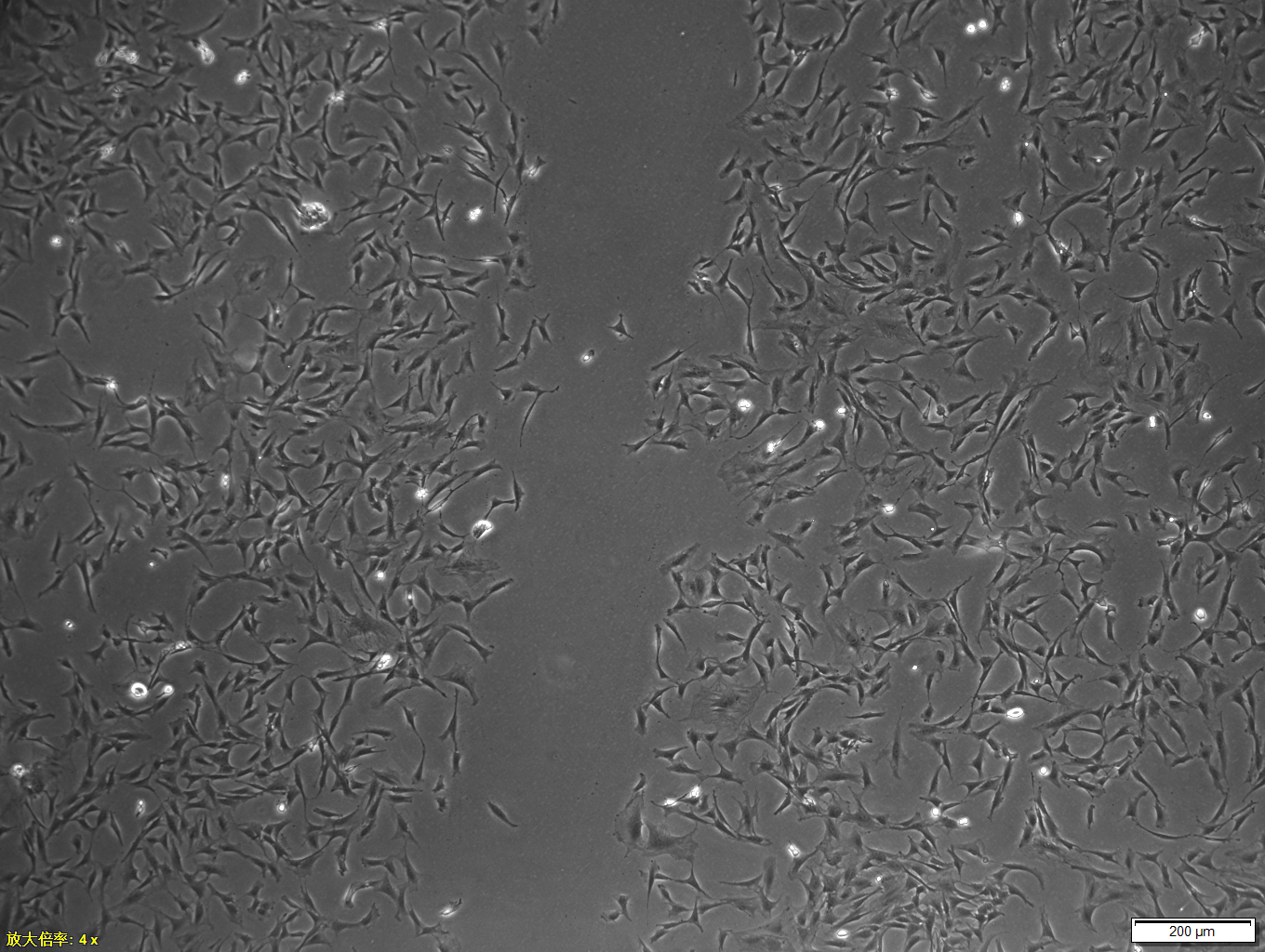

Supplement: S4 File — (ZIP) [file pone.0294566.s004.zip › support information/scrach/12h/sh-PHD2/scrach-12h-shphd2-2.tif]

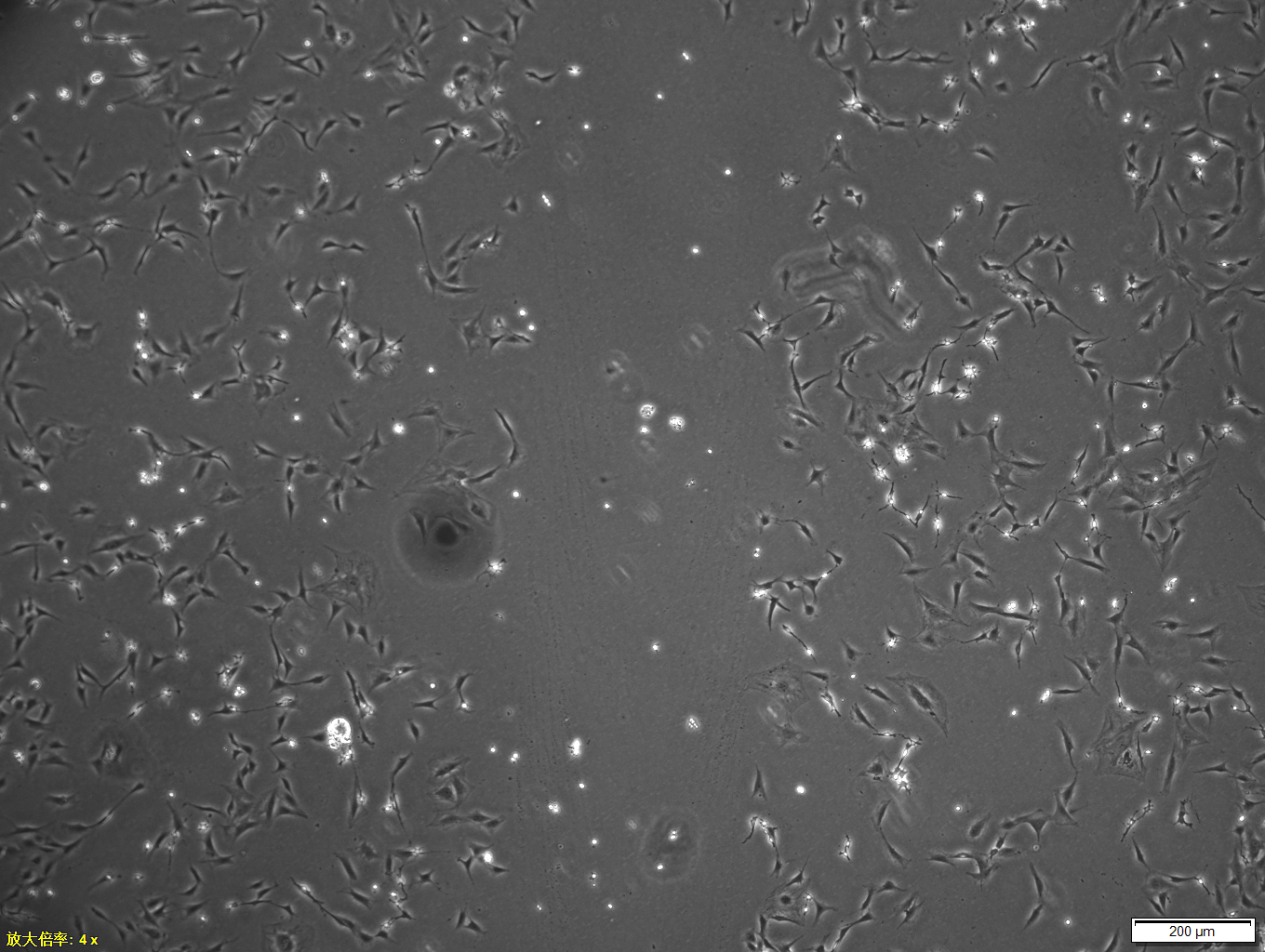

Supplement: S4 File — (ZIP) [file pone.0294566.s004.zip › support information/scrach/12h/sh-PHD2+8um Dorsomorphin/scrach-12h-sh-PHD2+8um Dorsomorphin-1.tif]

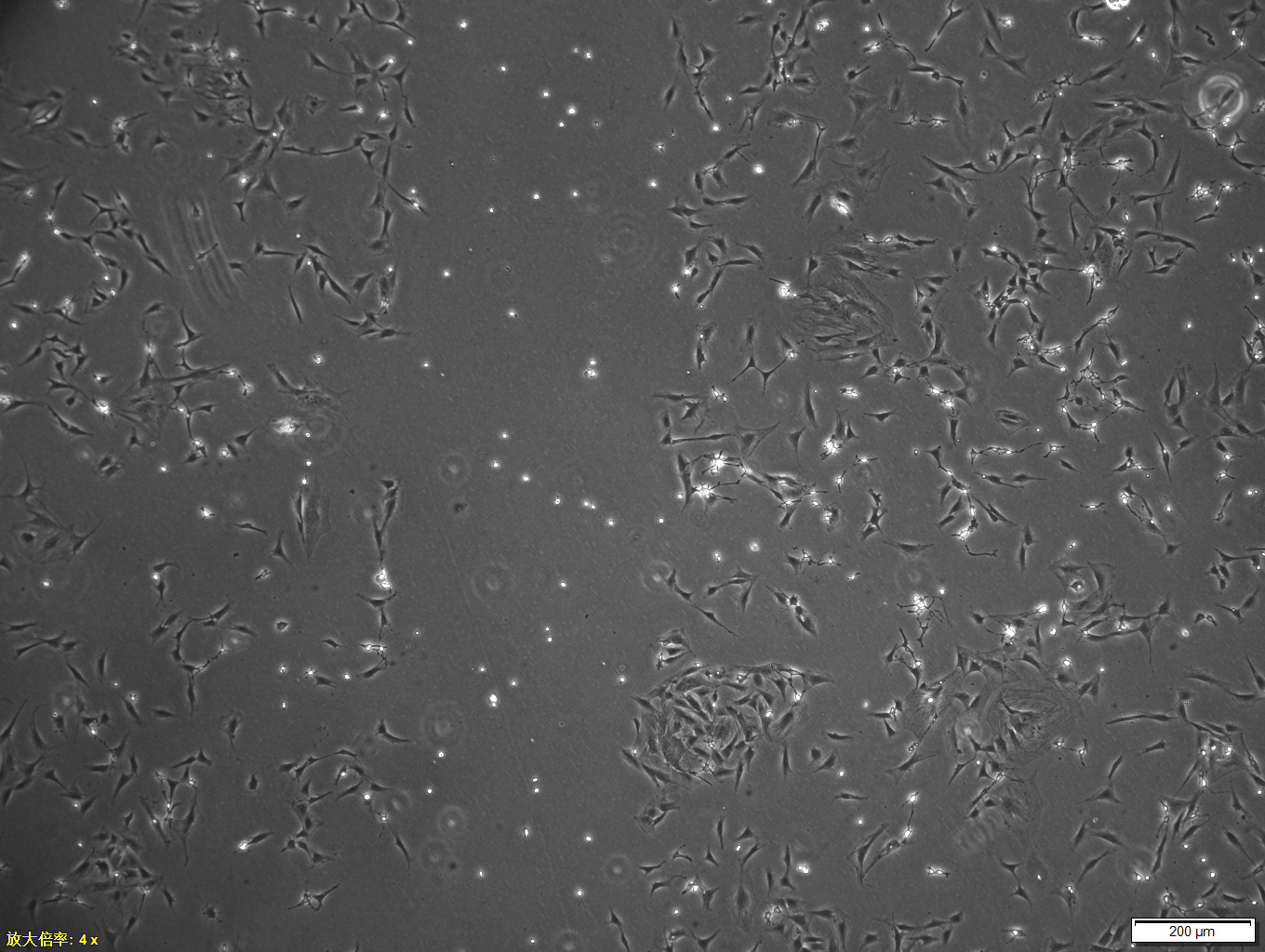

Supplement: S4 File — (ZIP) [file pone.0294566.s004.zip › support information/scrach/12h/sh-PHD2+8um Dorsomorphin/scrach-12h-sh-PHD2+8um Dorsomorphin-2.tif]

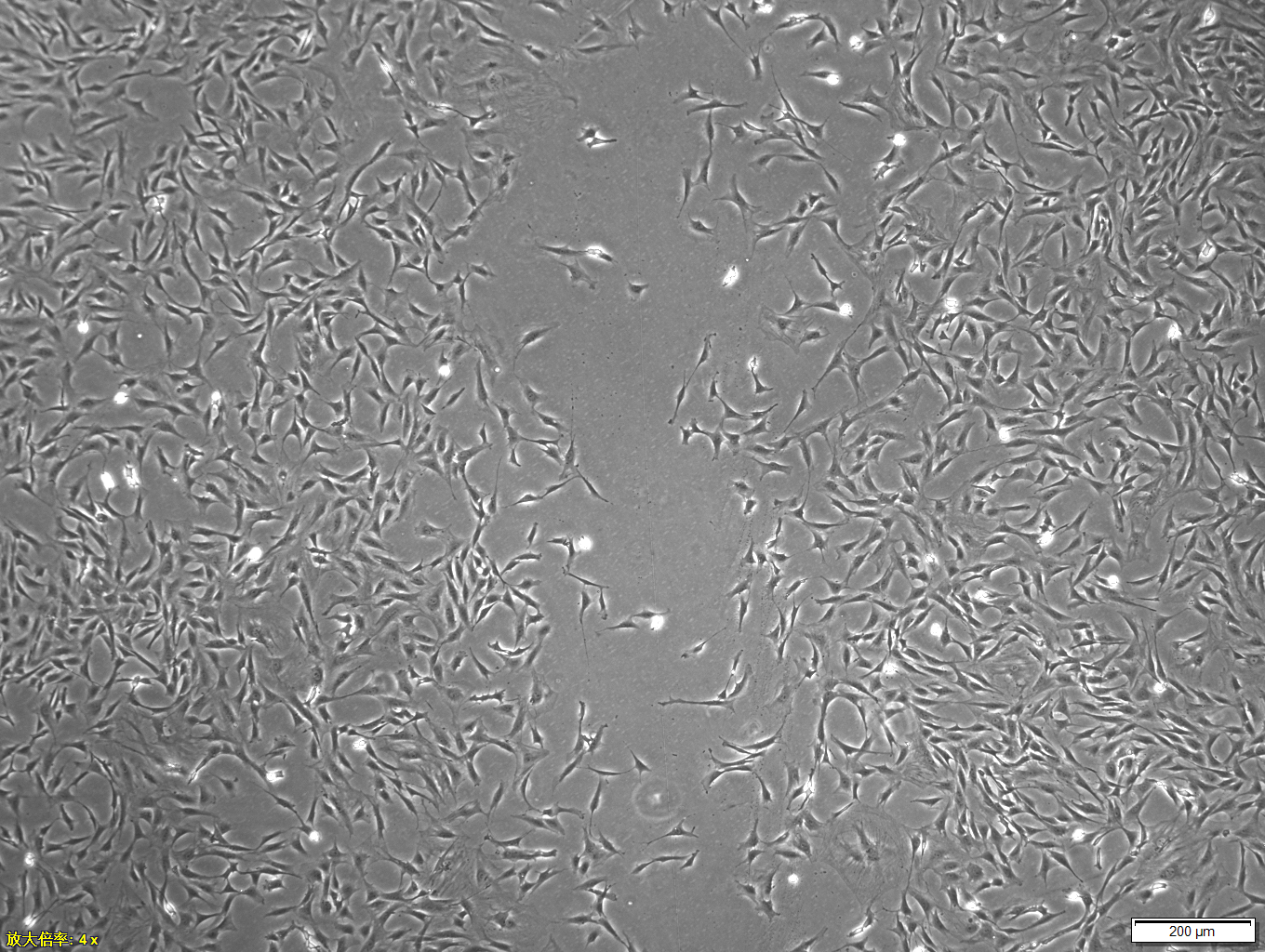

Supplement: S4 File — (ZIP) [file pone.0294566.s004.zip › support information/scrach/24h/sh-Control/scrach-24h-shcontrol-1.tif]

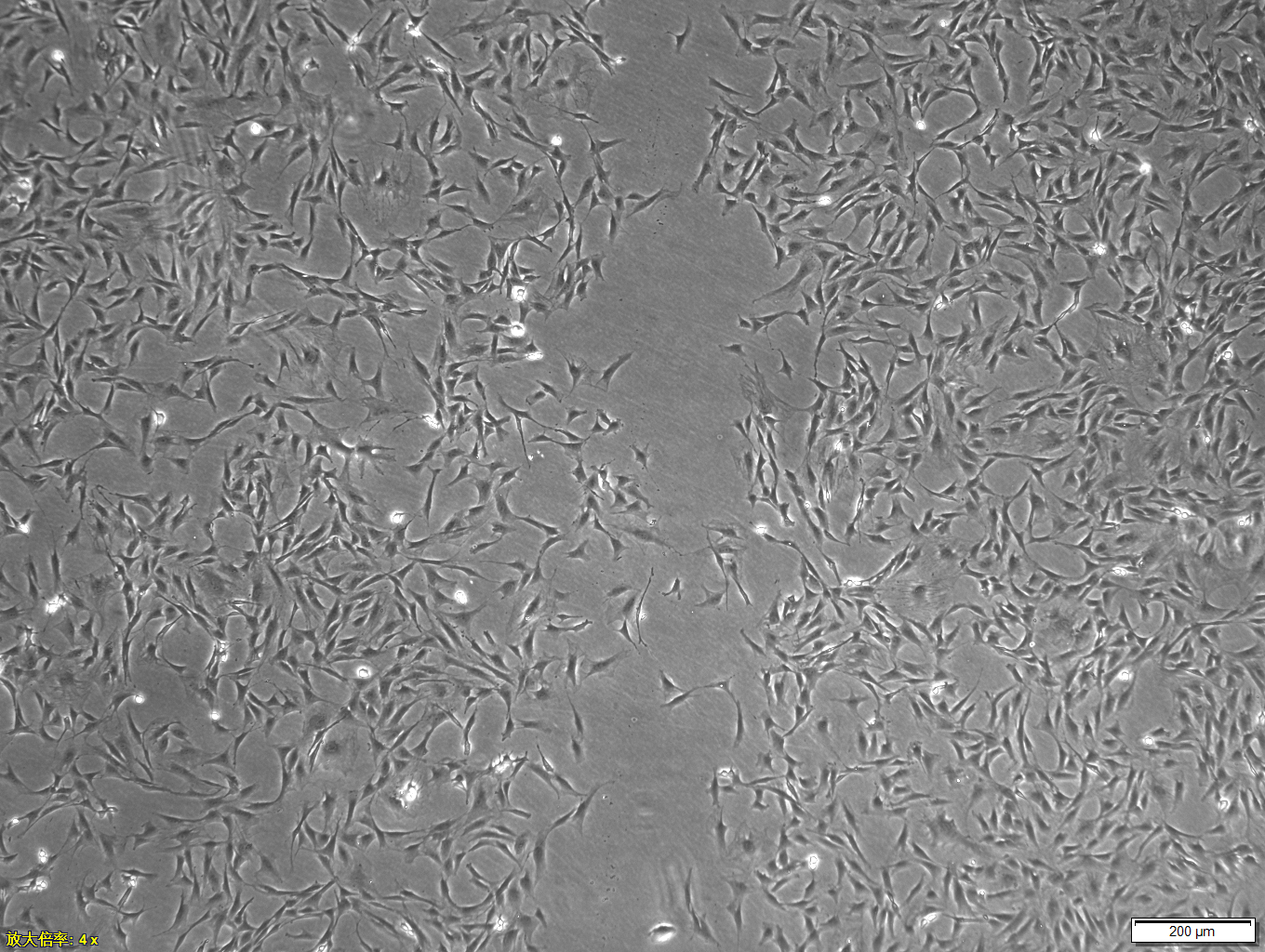

Supplement: S4 File — (ZIP) [file pone.0294566.s004.zip › support information/scrach/24h/sh-Control/scrach-24h-shcontrol-2.tif]

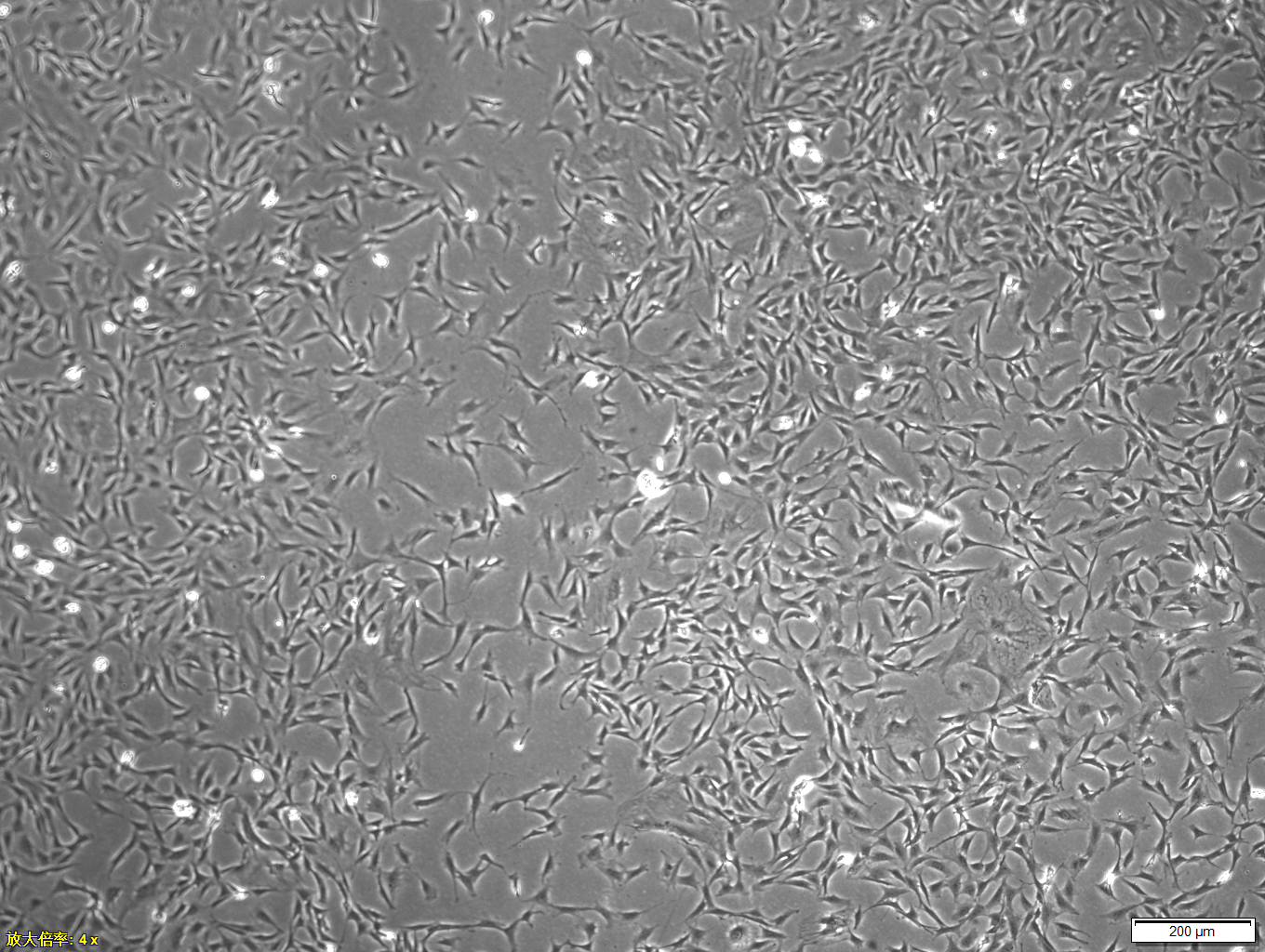

Supplement: S4 File — (ZIP) [file pone.0294566.s004.zip › support information/scrach/24h/sh-PHD2/scrach-24h-shphd2-1.tif]

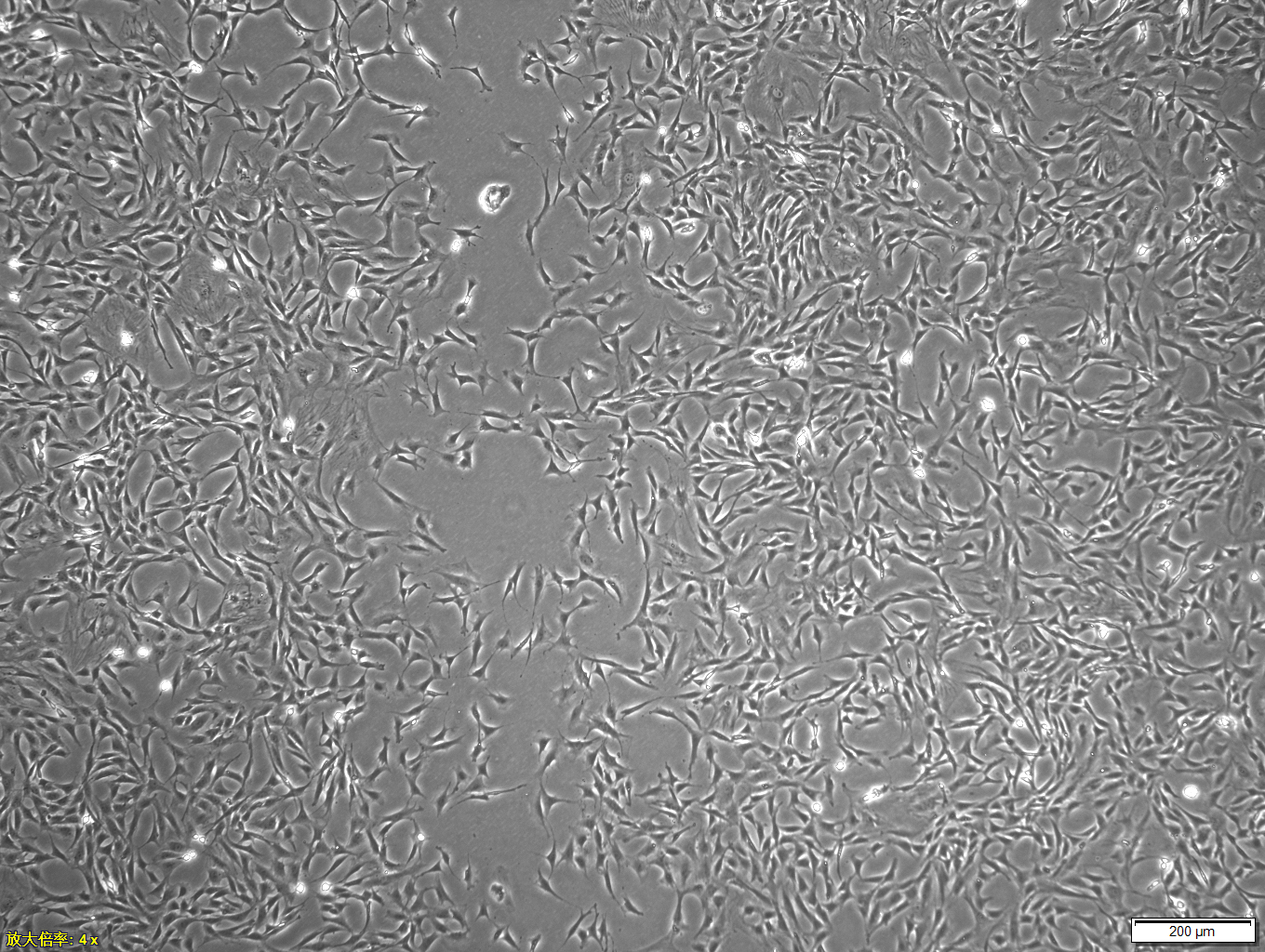

Supplement: S4 File — (ZIP) [file pone.0294566.s004.zip › support information/scrach/24h/sh-PHD2/scrach-24h-shphd2-2.tif]

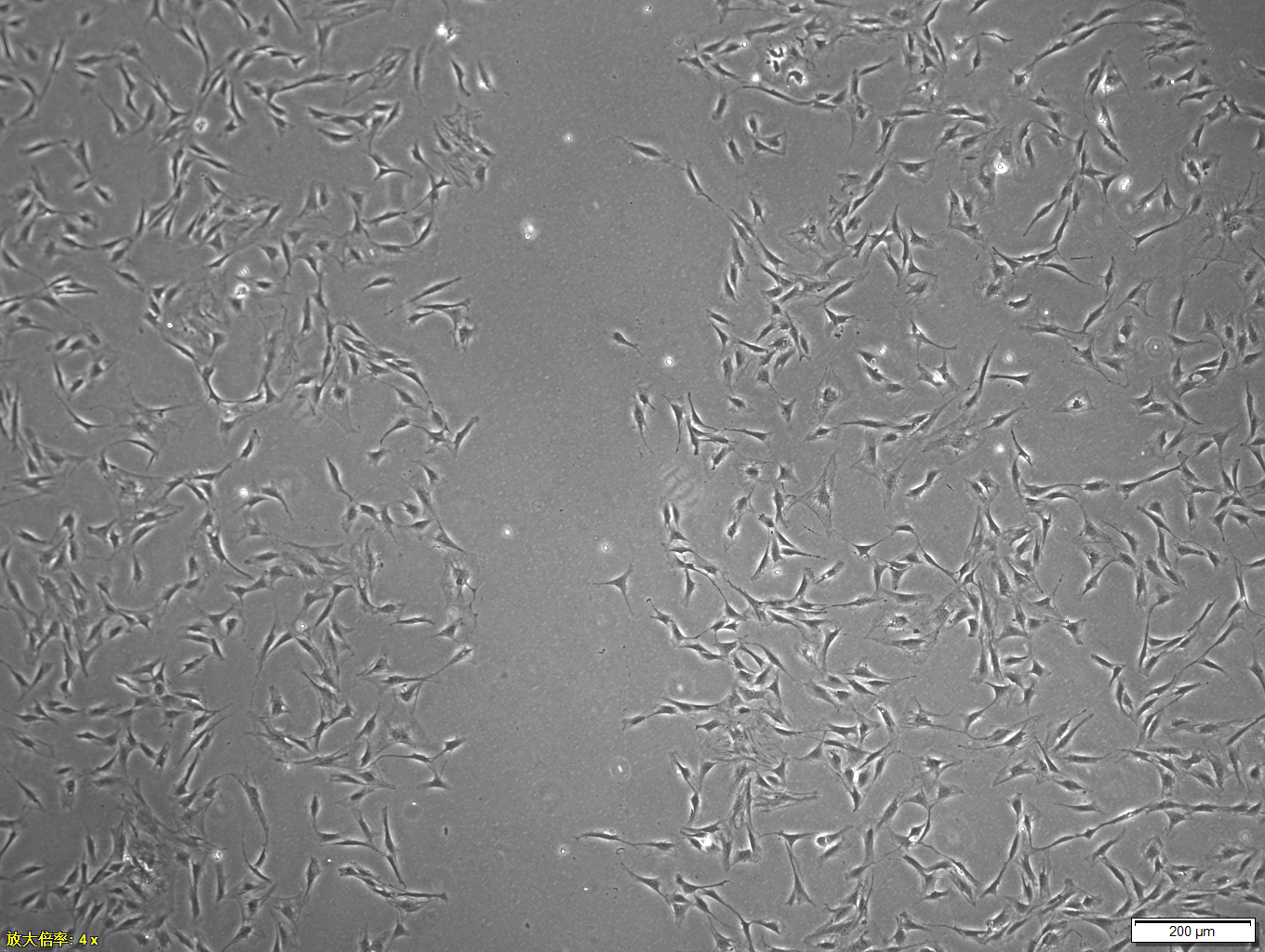

Supplement: S4 File — (ZIP) [file pone.0294566.s004.zip › support information/scrach/24h/sh-PHD2+8um Dorsomorphin/scrach-24h-sh-PHD2+8um Dorsomorphin-1.tif]

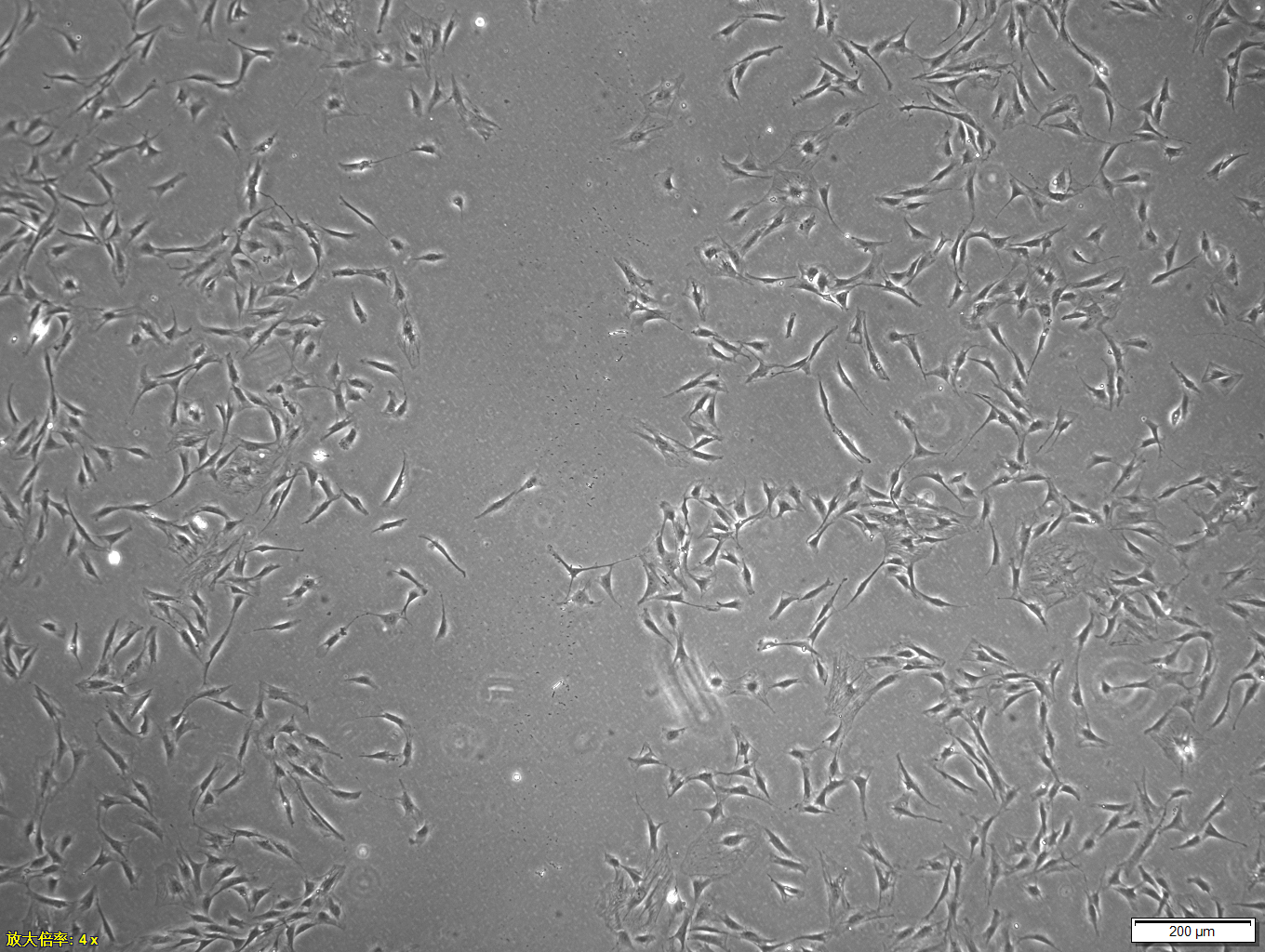

Supplement: S4 File — (ZIP) [file pone.0294566.s004.zip › support information/scrach/24h/sh-PHD2+8um Dorsomorphin/scrach-24h-sh-PHD2+8um Dorsomorphin-2.tif]

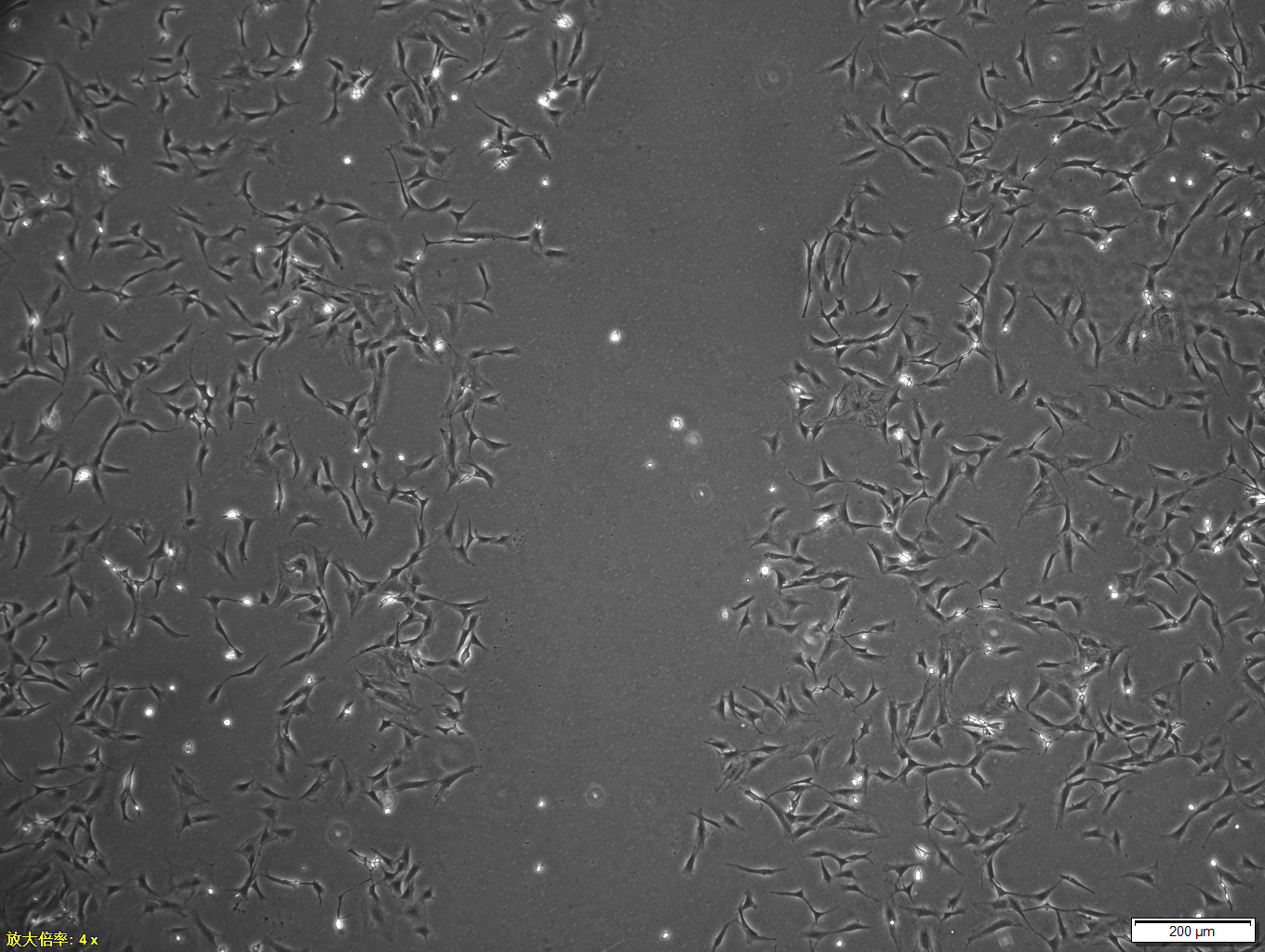

Supplement: S4 File — (ZIP) [file pone.0294566.s004.zip › support information/scrach/6h/sh-Control/scrach-6h-shcontrol-1.tif]

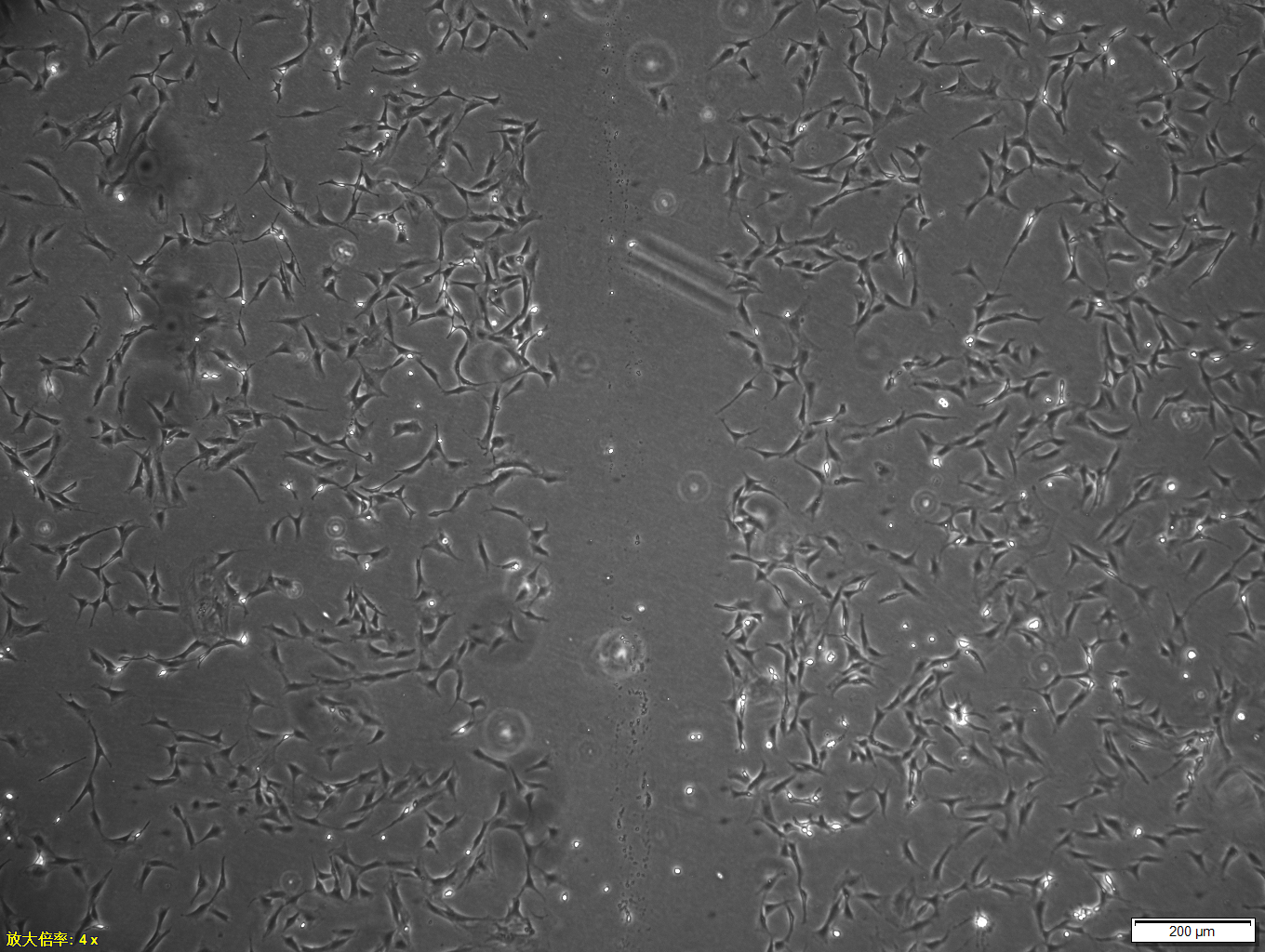

Supplement: S4 File — (ZIP) [file pone.0294566.s004.zip › support information/scrach/6h/sh-Control/scrach-6h-shcontrol-2.tif]

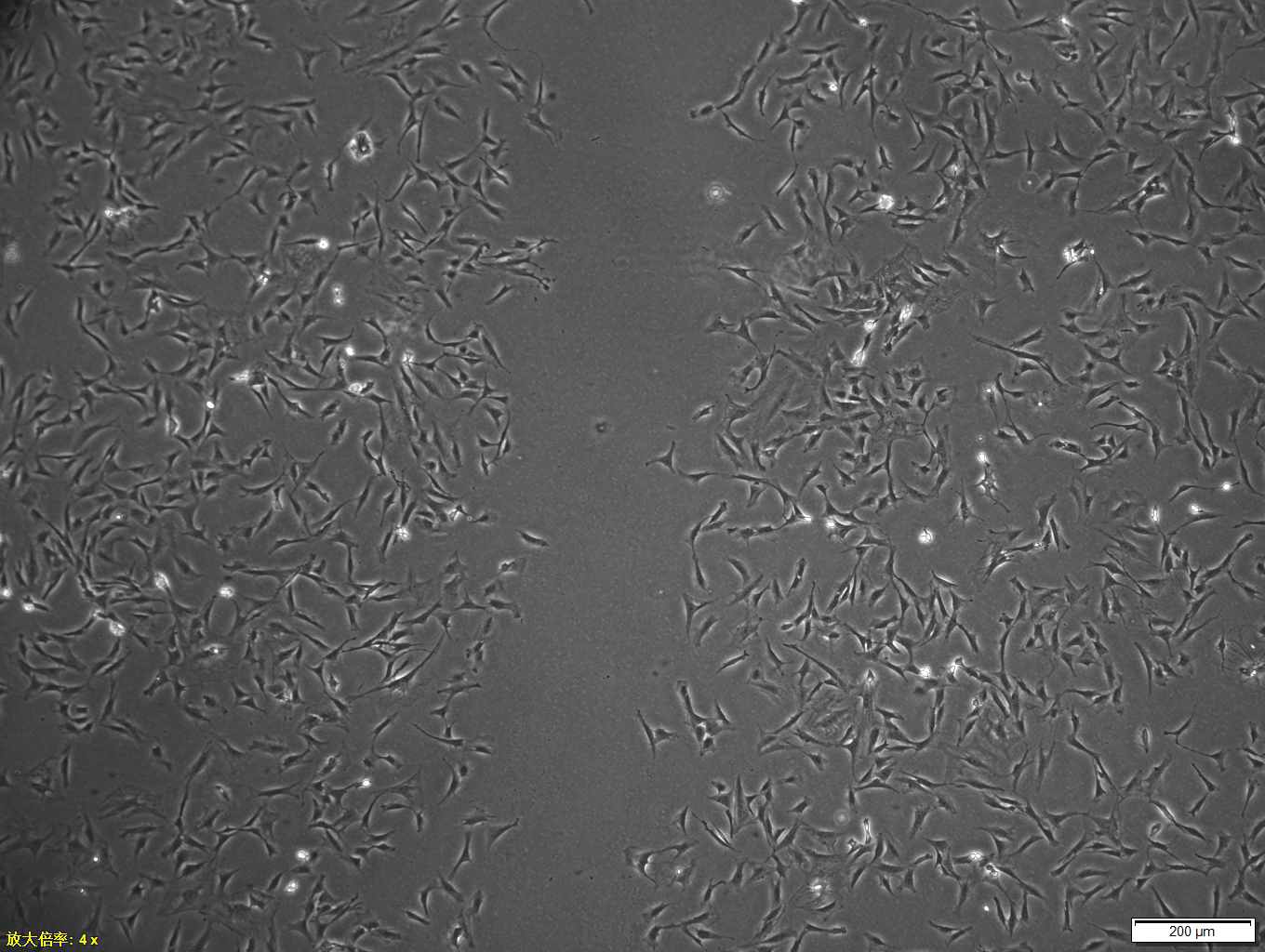

Supplement: S4 File — (ZIP) [file pone.0294566.s004.zip › support information/scrach/6h/sh-PHD2/scrach-6h-shphd2-1.tif]

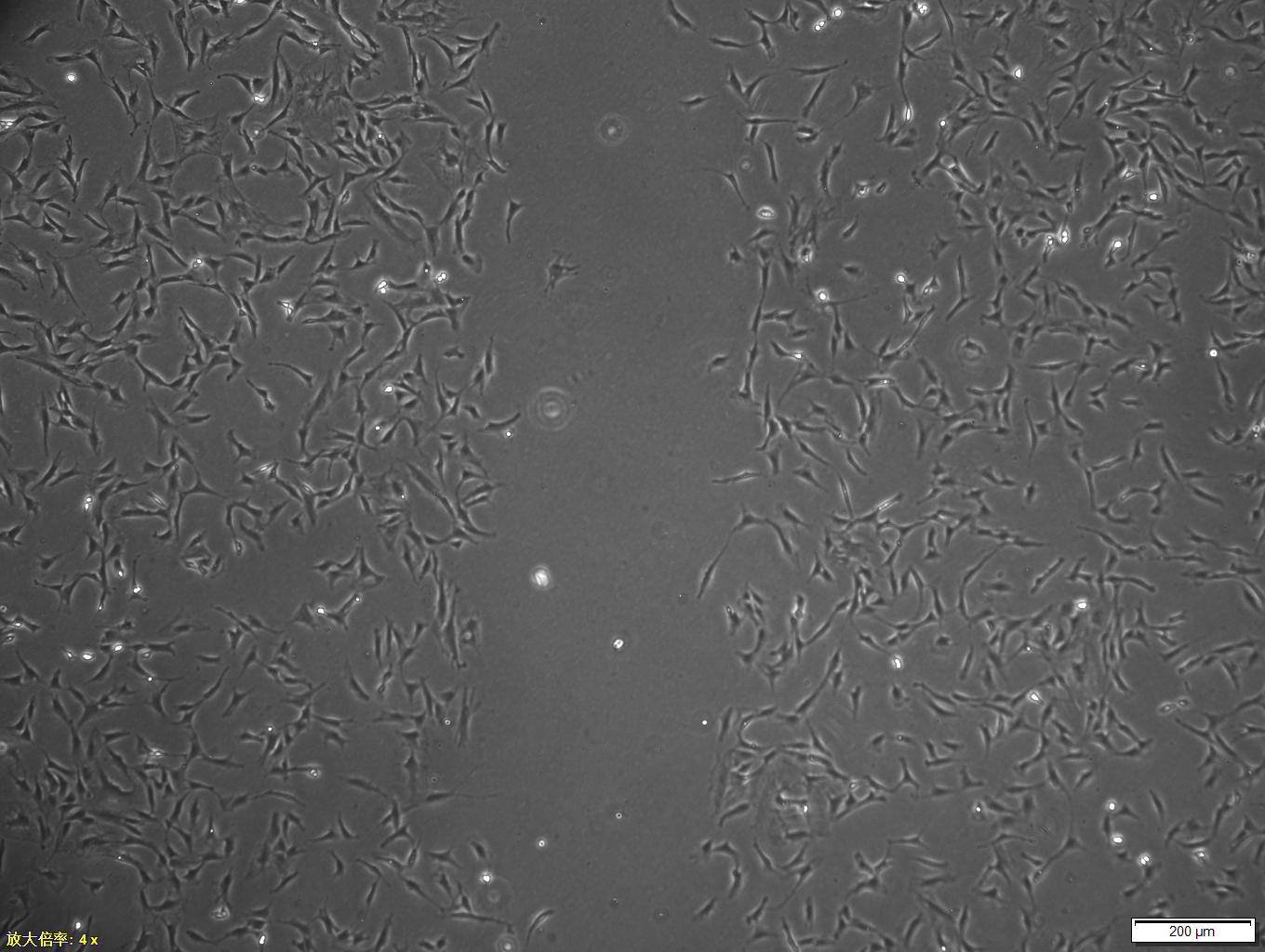

Supplement: S4 File — (ZIP) [file pone.0294566.s004.zip › support information/scrach/6h/sh-PHD2/scrach-6h-shphd2-2.tif]

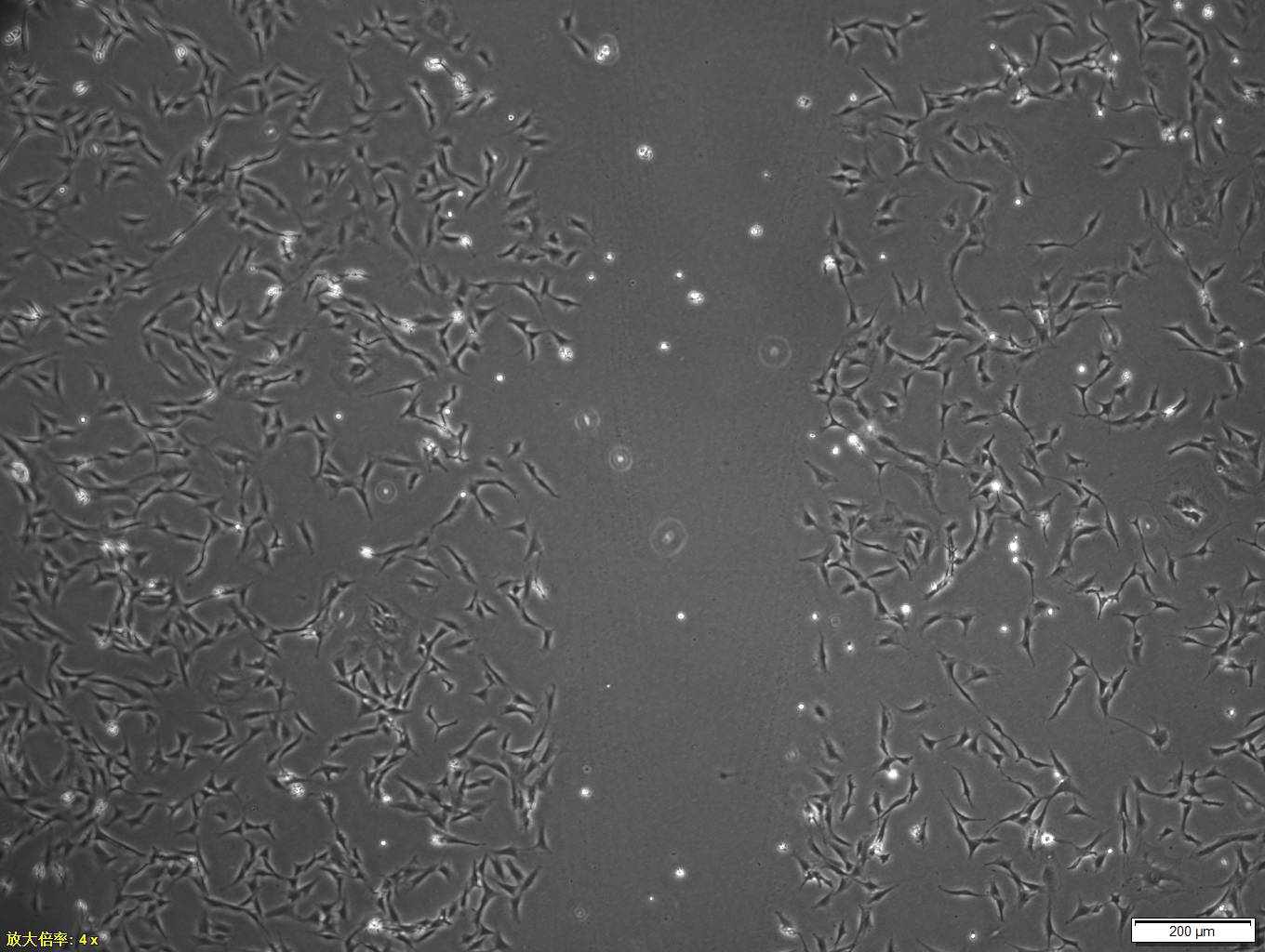

Supplement: S4 File — (ZIP) [file pone.0294566.s004.zip › support information/scrach/6h/sh-PHD2+8um Dorsomorphin/scrach-6h-sh-PHD2+8um Dorsomorphin-1.tif]

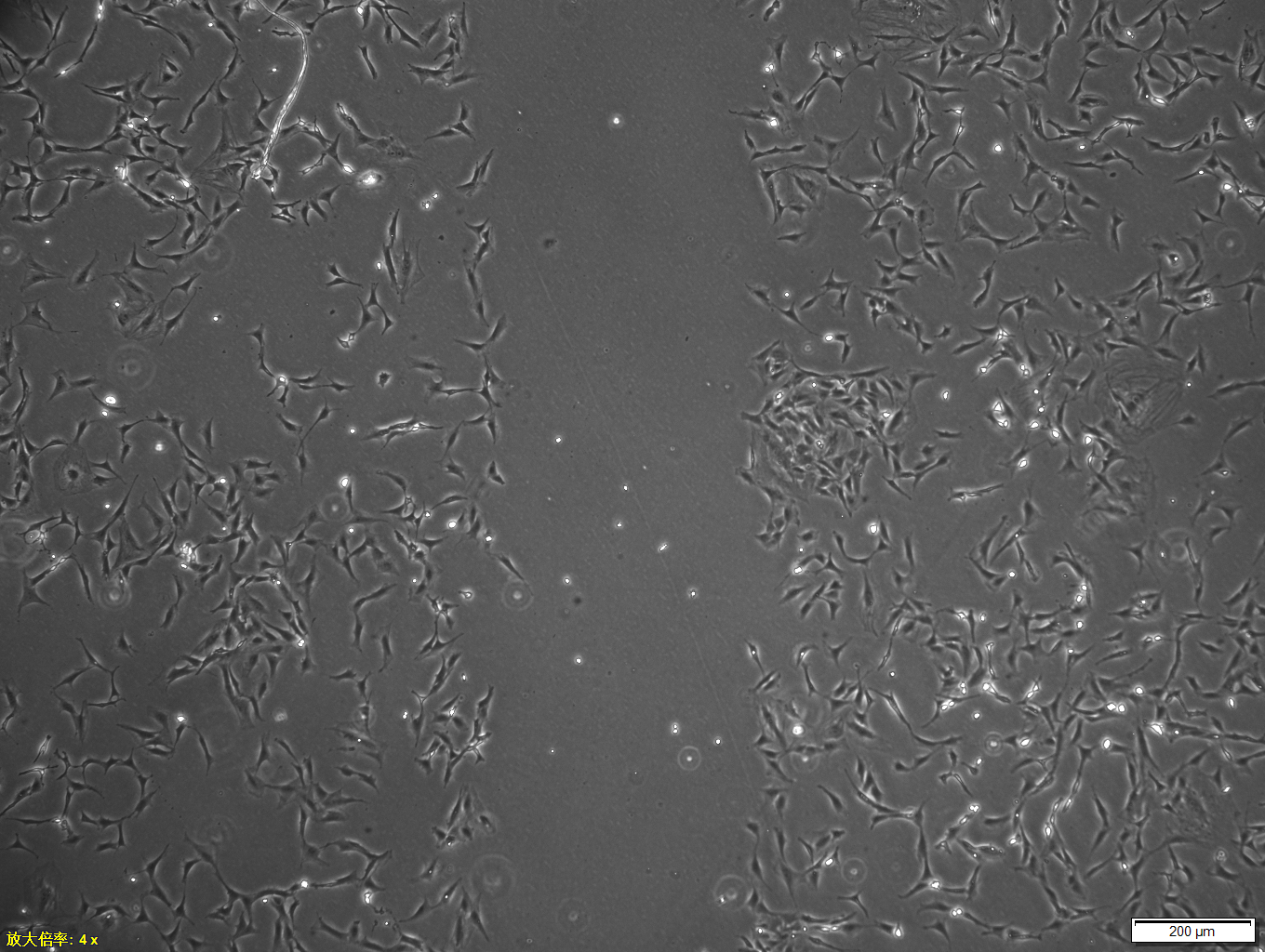

Supplement: S4 File — (ZIP) [file pone.0294566.s004.zip › support information/scrach/6h/sh-PHD2+8um Dorsomorphin/scrach-6h-sh-PHD2+8um Dorsomorphin-2.tif]

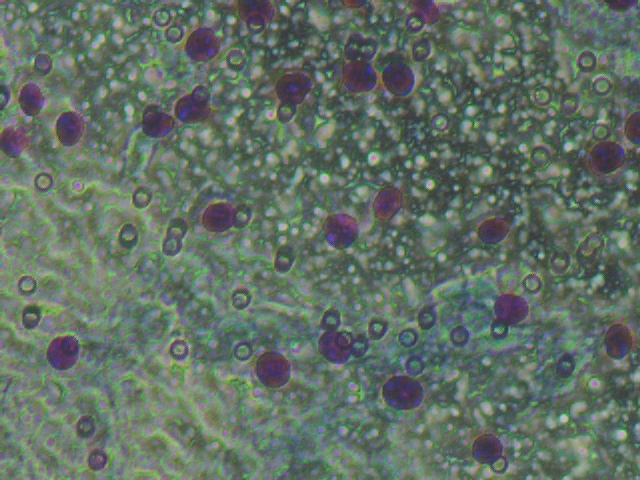

Supplement: S5 File — (ZIP) [file pone.0294566.s005.zip › support information/transwell/sh-Control/transwell-shcontrol-1.jpg]

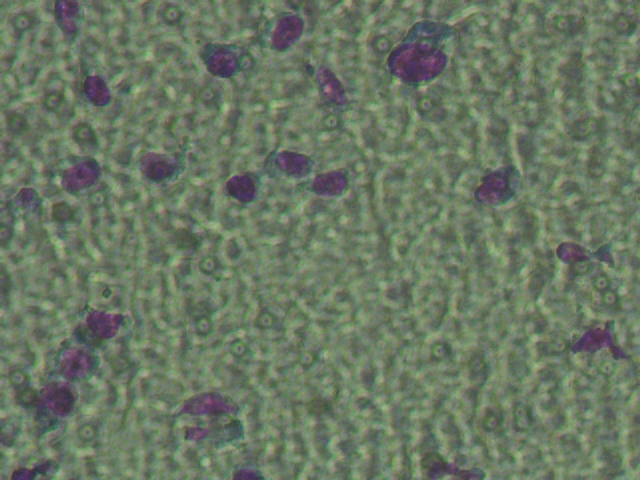

Supplement: S5 File — (ZIP) [file pone.0294566.s005.zip › support information/transwell/sh-Control/transwell-shcontrol-10.jpg]

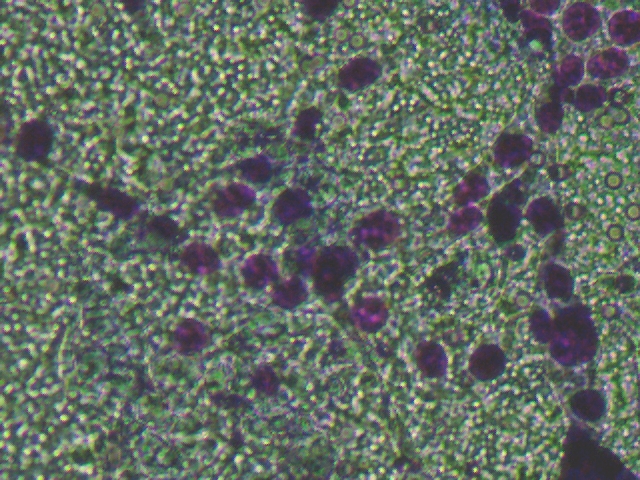

Supplement: S5 File — (ZIP) [file pone.0294566.s005.zip › support information/transwell/sh-Control/transwell-shcontrol-2.jpg]

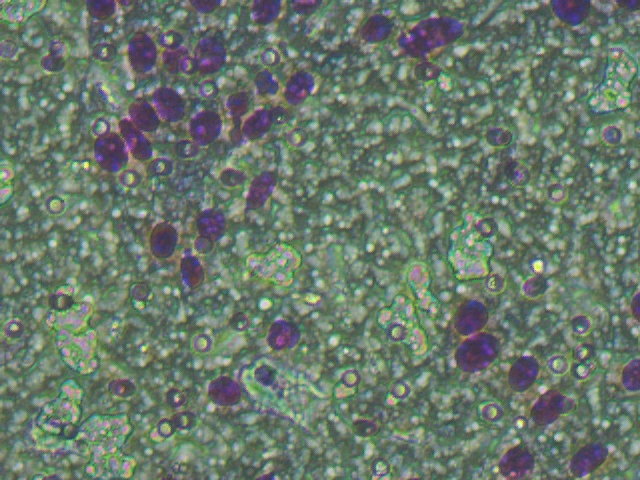

Supplement: S5 File — (ZIP) [file pone.0294566.s005.zip › support information/transwell/sh-Control/transwell-shcontrol-3.jpg]

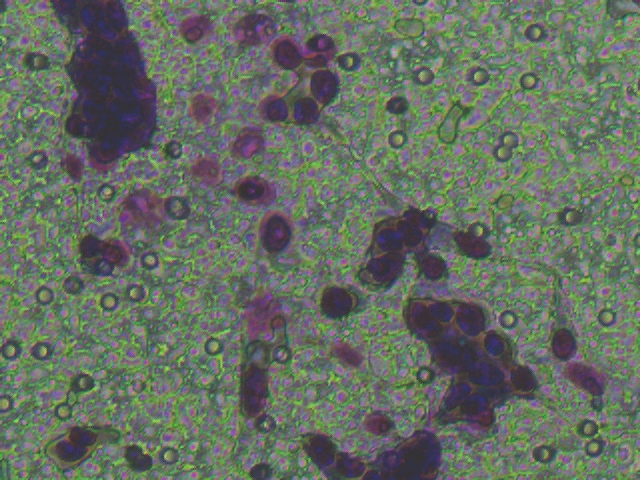

Supplement: S5 File — (ZIP) [file pone.0294566.s005.zip › support information/transwell/sh-Control/transwell-shcontrol-4.jpg]

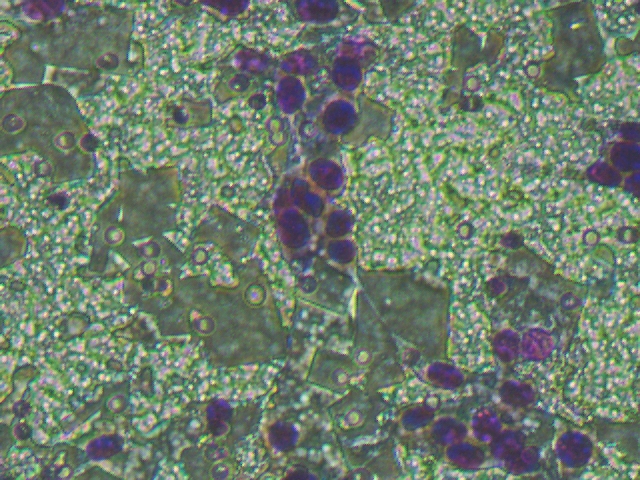

Supplement: S5 File — (ZIP) [file pone.0294566.s005.zip › support information/transwell/sh-Control/transwell-shcontrol-5.jpg]

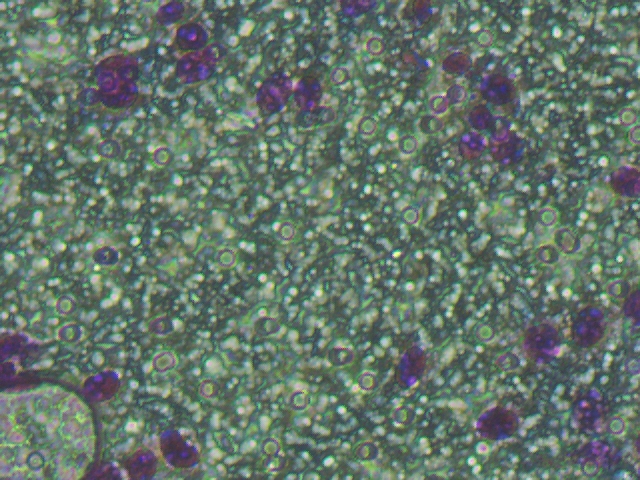

Supplement: S5 File — (ZIP) [file pone.0294566.s005.zip › support information/transwell/sh-Control/transwell-shcontrol-6.jpg]

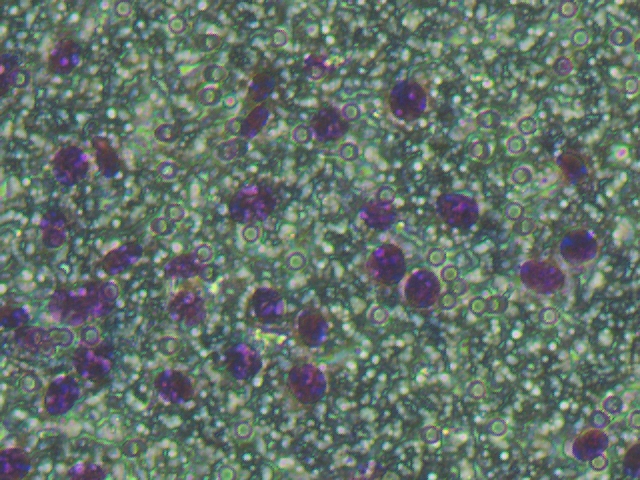

Supplement: S5 File — (ZIP) [file pone.0294566.s005.zip › support information/transwell/sh-Control/transwell-shcontrol-7.jpg]

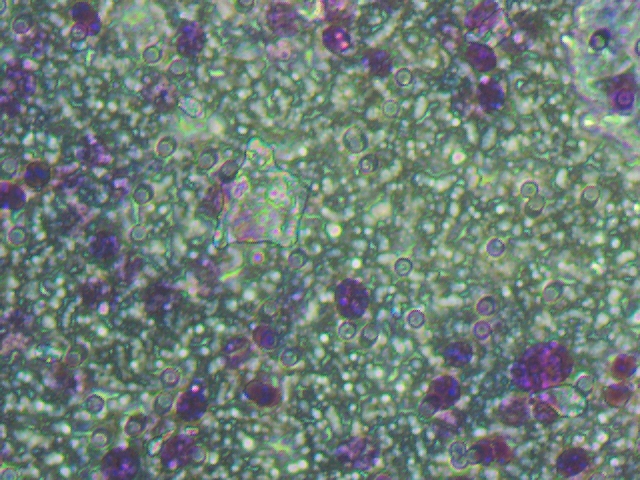

Supplement: S5 File — (ZIP) [file pone.0294566.s005.zip › support information/transwell/sh-Control/transwell-shcontrol-8.jpg]

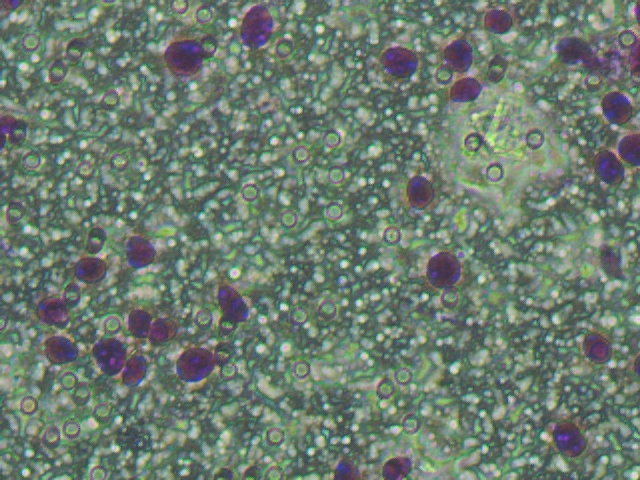

Supplement: S5 File — (ZIP) [file pone.0294566.s005.zip › support information/transwell/sh-Control/transwell-shcontrol-9.jpg]

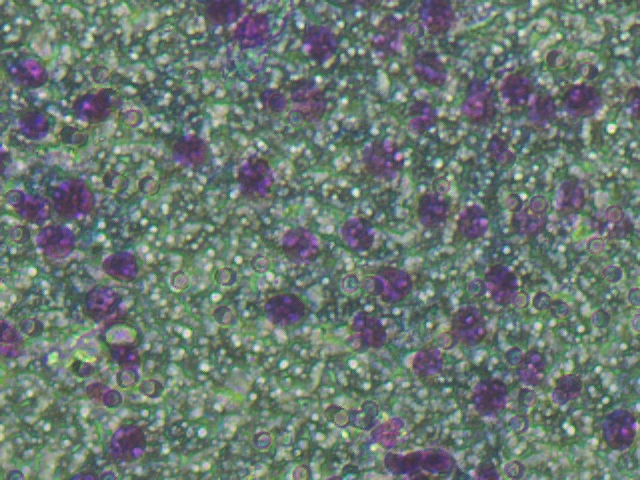

Supplement: S5 File — (ZIP) [file pone.0294566.s005.zip › support information/transwell/sh-PHD2/transwell-sh-PHD2- (1).jpg]

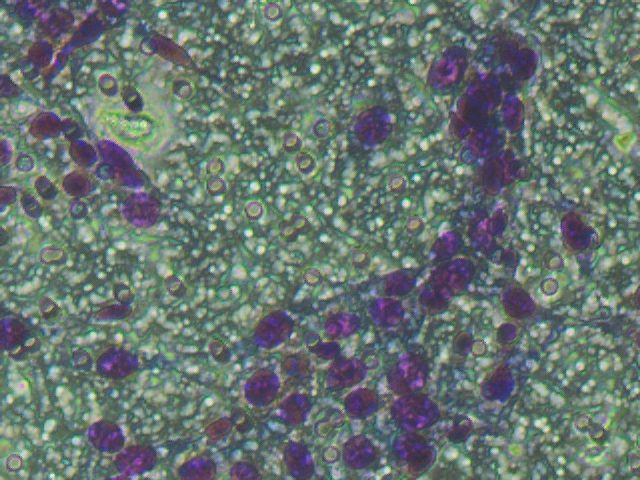

Supplement: S5 File — (ZIP) [file pone.0294566.s005.zip › support information/transwell/sh-PHD2/transwell-sh-PHD2- (10).jpg]

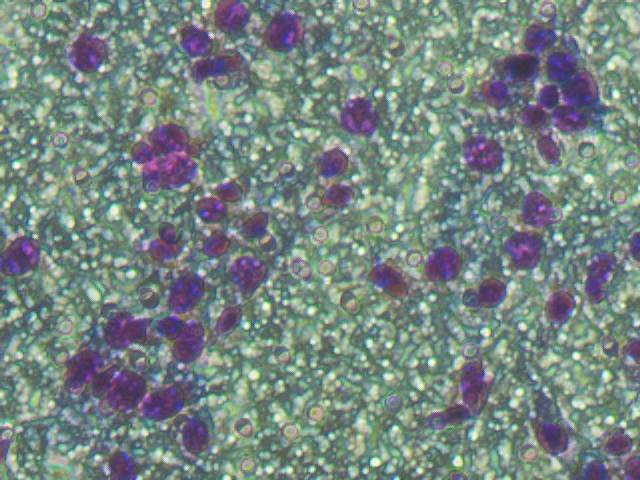

Supplement: S5 File — (ZIP) [file pone.0294566.s005.zip › support information/transwell/sh-PHD2/transwell-sh-PHD2- (11).jpg]

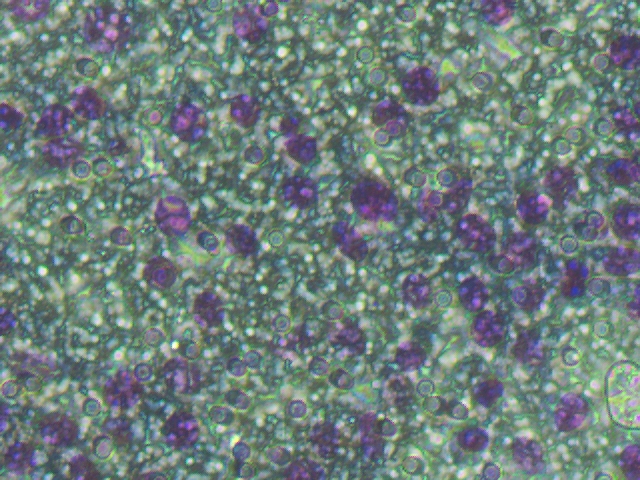

Supplement: S5 File — (ZIP) [file pone.0294566.s005.zip › support information/transwell/sh-PHD2/transwell-sh-PHD2- (12).jpg]

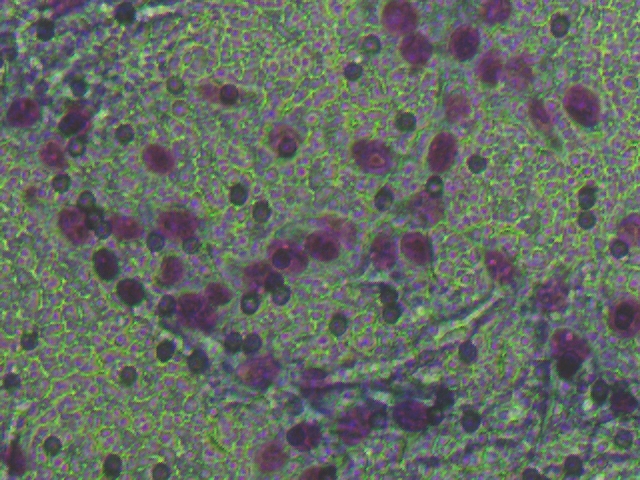

Supplement: S5 File — (ZIP) [file pone.0294566.s005.zip › support information/transwell/sh-PHD2/transwell-sh-PHD2- (13).jpg]

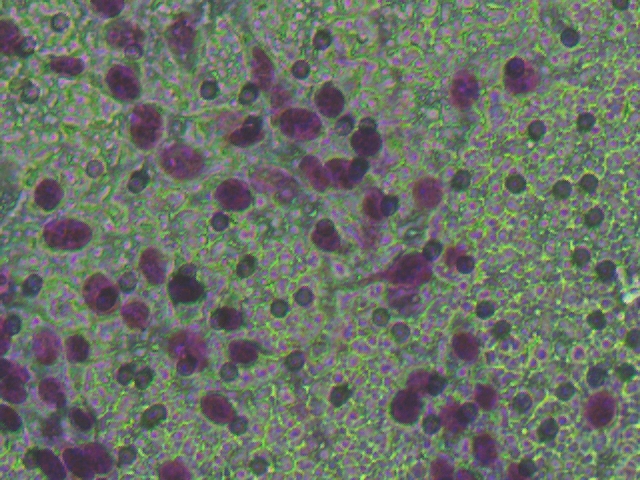

Supplement: S5 File — (ZIP) [file pone.0294566.s005.zip › support information/transwell/sh-PHD2/transwell-sh-PHD2- (14).jpg]

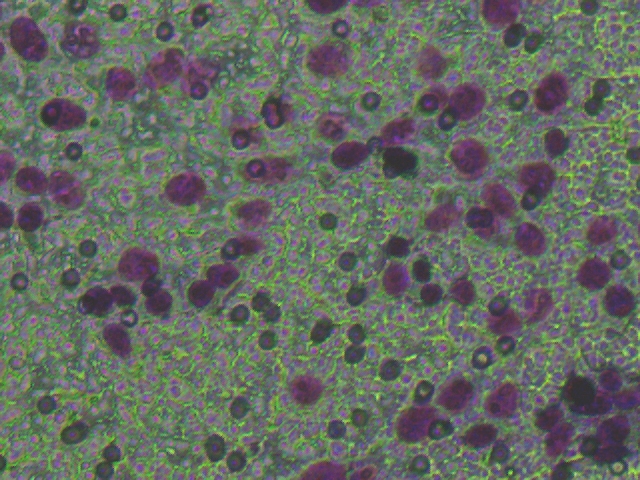

Supplement: S5 File — (ZIP) [file pone.0294566.s005.zip › support information/transwell/sh-PHD2/transwell-sh-PHD2- (15).jpg]

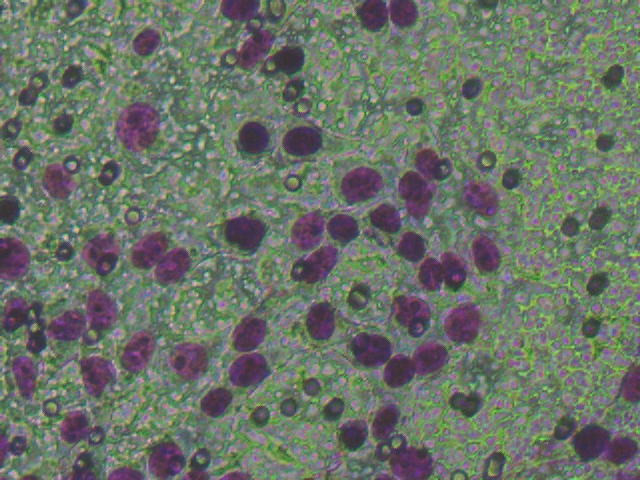

Supplement: S5 File — (ZIP) [file pone.0294566.s005.zip › support information/transwell/sh-PHD2/transwell-sh-PHD2- (16).jpg]

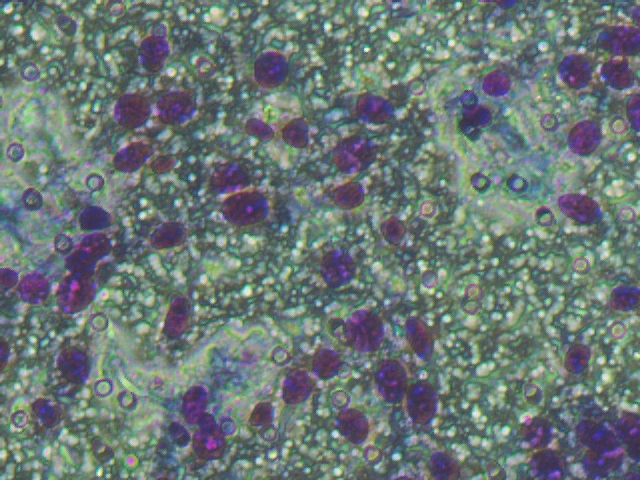

Supplement: S5 File — (ZIP) [file pone.0294566.s005.zip › support information/transwell/sh-PHD2/transwell-sh-PHD2- (17).jpg]

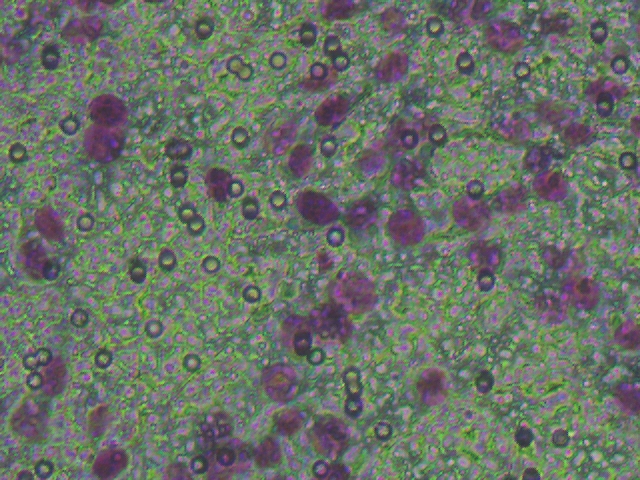

Supplement: S5 File — (ZIP) [file pone.0294566.s005.zip › support information/transwell/sh-PHD2/transwell-sh-PHD2- (18).jpg]

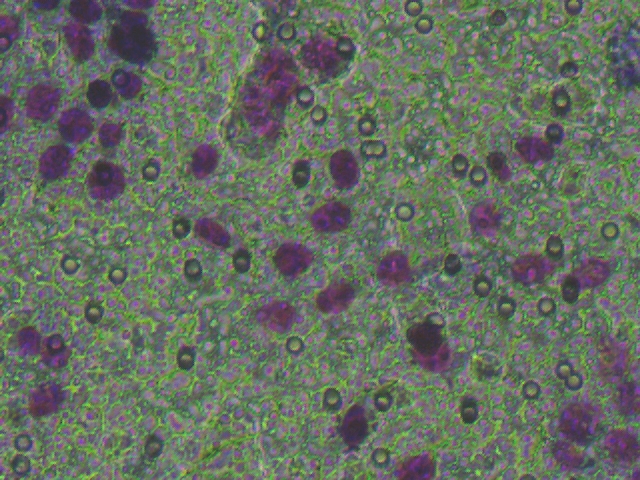

Supplement: S5 File — (ZIP) [file pone.0294566.s005.zip › support information/transwell/sh-PHD2/transwell-sh-PHD2- (19).jpg]

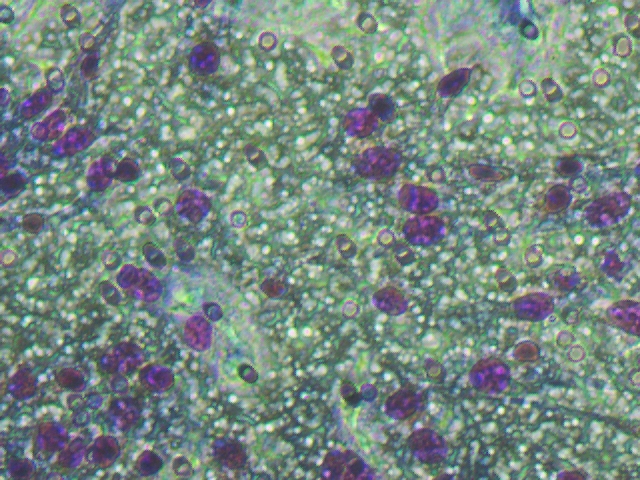

Supplement: S5 File — (ZIP) [file pone.0294566.s005.zip › support information/transwell/sh-PHD2/transwell-sh-PHD2- (2).jpg]

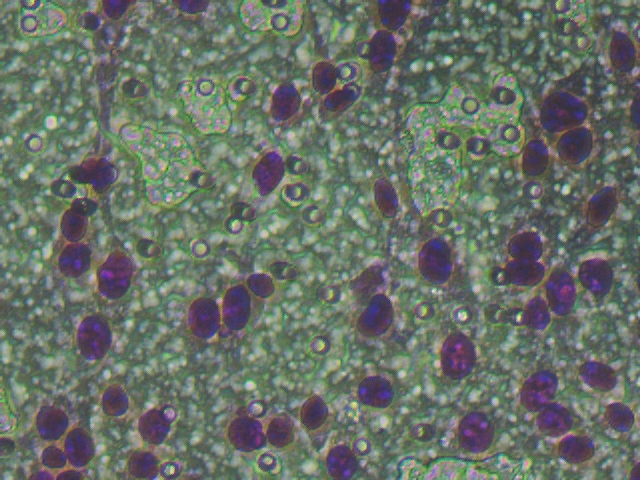

Supplement: S5 File — (ZIP) [file pone.0294566.s005.zip › support information/transwell/sh-PHD2/transwell-sh-PHD2- (20).jpg]

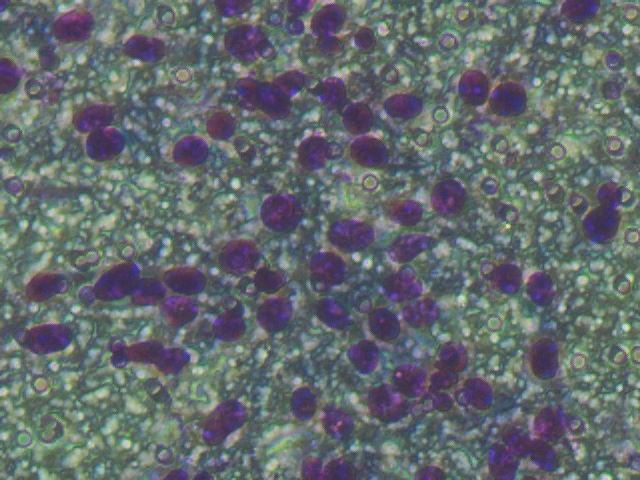

Supplement: S5 File — (ZIP) [file pone.0294566.s005.zip › support information/transwell/sh-PHD2/transwell-sh-PHD2- (21).jpg]

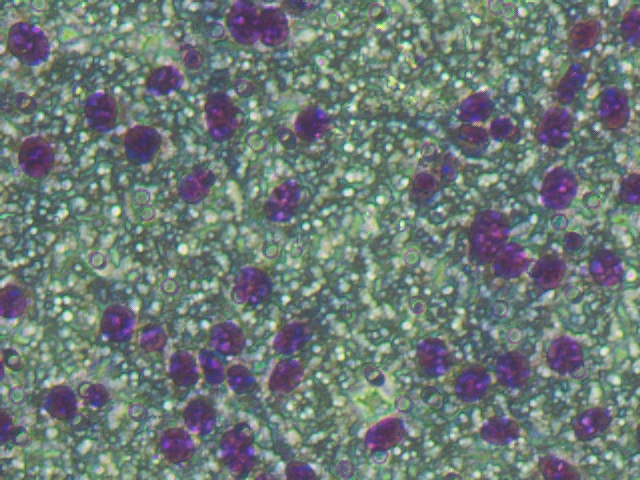

Supplement: S5 File — (ZIP) [file pone.0294566.s005.zip › support information/transwell/sh-PHD2/transwell-sh-PHD2- (22).jpg]

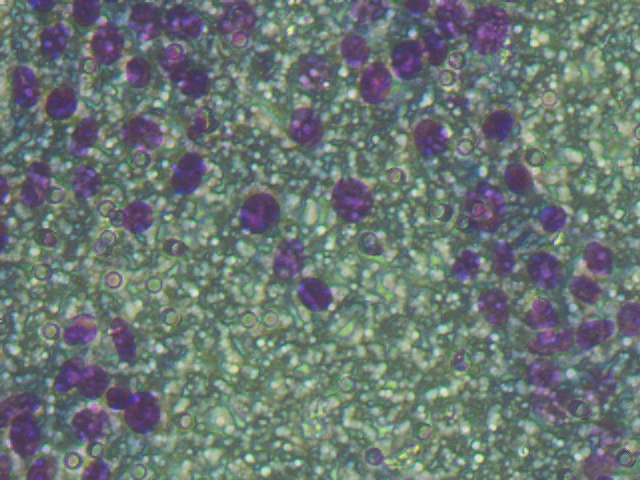

Supplement: S5 File — (ZIP) [file pone.0294566.s005.zip › support information/transwell/sh-PHD2/transwell-sh-PHD2- (23).jpg]

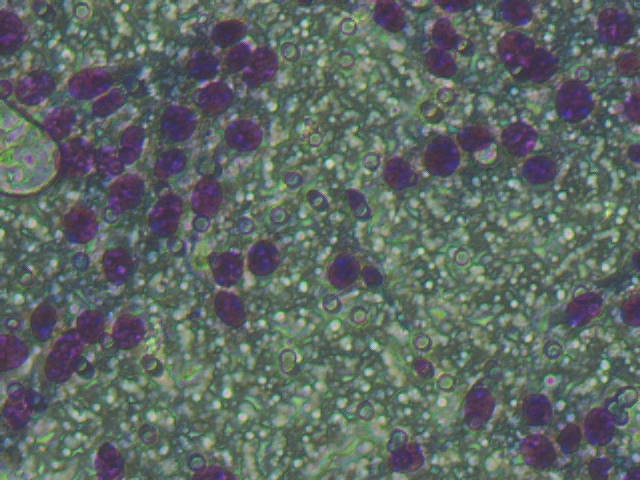

Supplement: S5 File — (ZIP) [file pone.0294566.s005.zip › support information/transwell/sh-PHD2/transwell-sh-PHD2- (24).jpg]

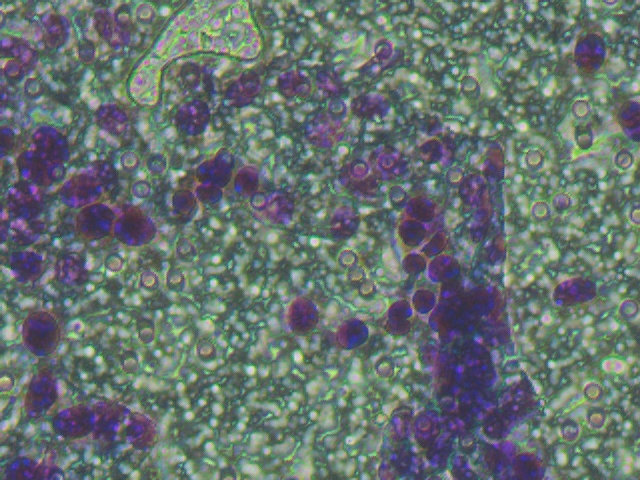

Supplement: S5 File — (ZIP) [file pone.0294566.s005.zip › support information/transwell/sh-PHD2/transwell-sh-PHD2- (3).jpg]

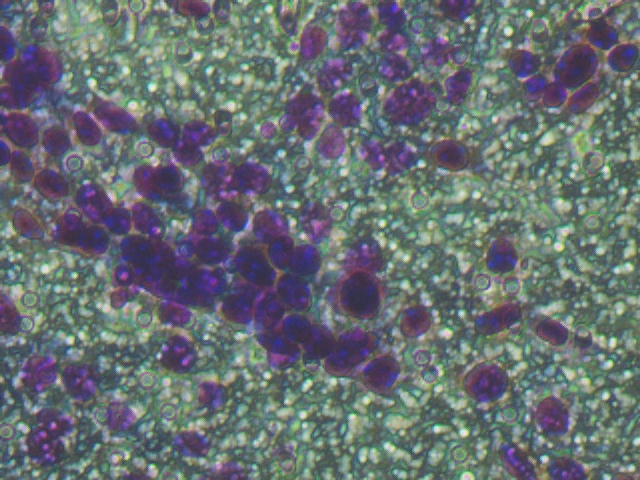

Supplement: S5 File — (ZIP) [file pone.0294566.s005.zip › support information/transwell/sh-PHD2/transwell-sh-PHD2- (4).jpg]

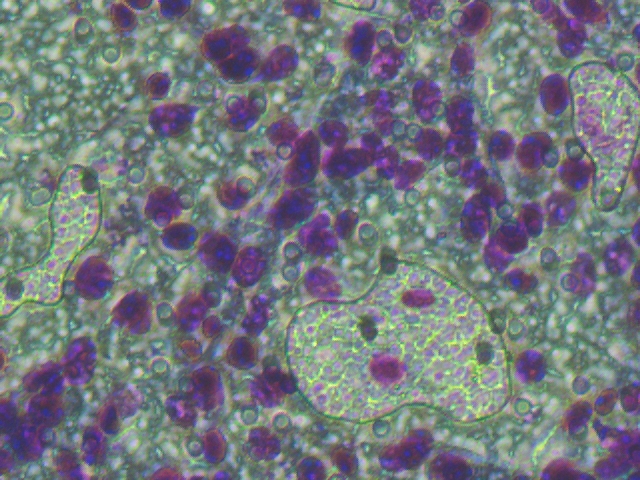

Supplement: S5 File — (ZIP) [file pone.0294566.s005.zip › support information/transwell/sh-PHD2/transwell-sh-PHD2- (5).jpg]

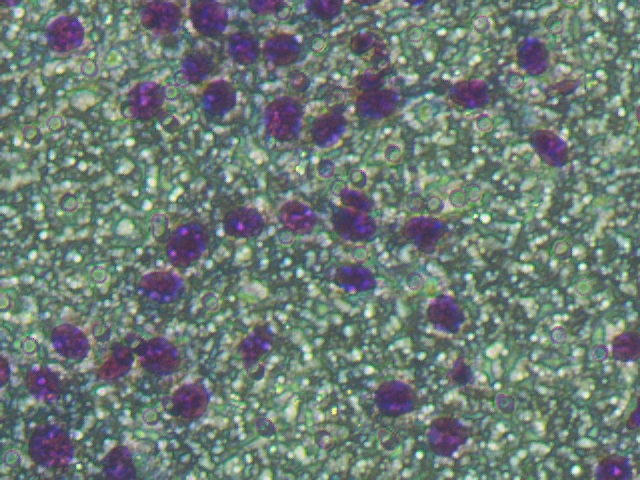

Supplement: S5 File — (ZIP) [file pone.0294566.s005.zip › support information/transwell/sh-PHD2/transwell-sh-PHD2- (6).jpg]

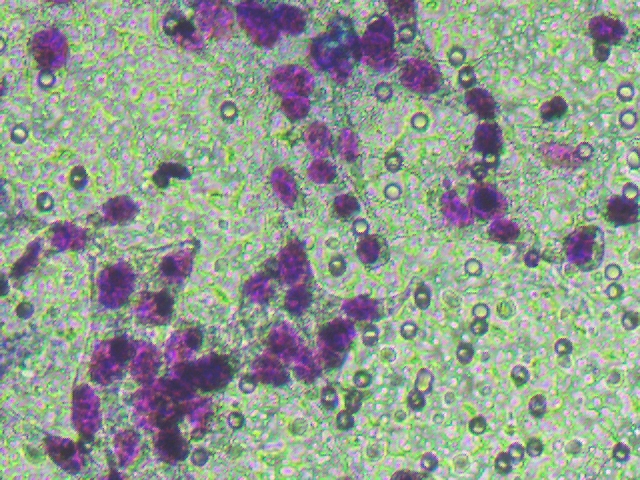

Supplement: S5 File — (ZIP) [file pone.0294566.s005.zip › support information/transwell/sh-PHD2/transwell-sh-PHD2- (7).jpg]

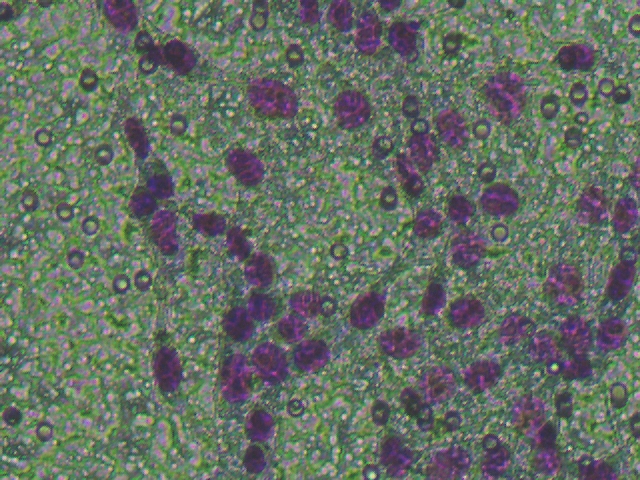

Supplement: S5 File — (ZIP) [file pone.0294566.s005.zip › support information/transwell/sh-PHD2/transwell-sh-PHD2- (8).jpg]

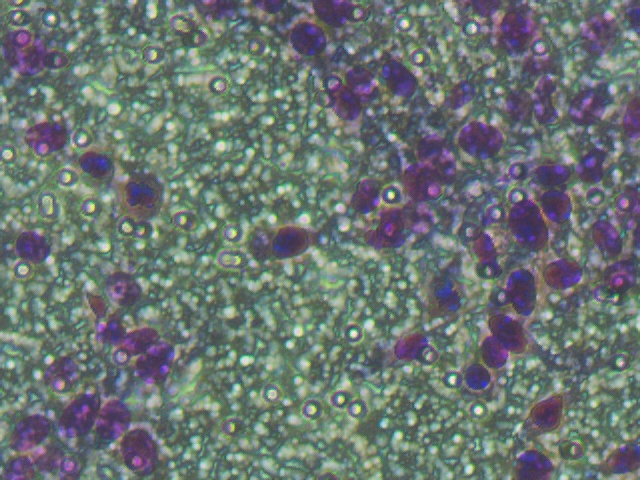

Supplement: S5 File — (ZIP) [file pone.0294566.s005.zip › support information/transwell/sh-PHD2/transwell-sh-PHD2- (9).jpg]

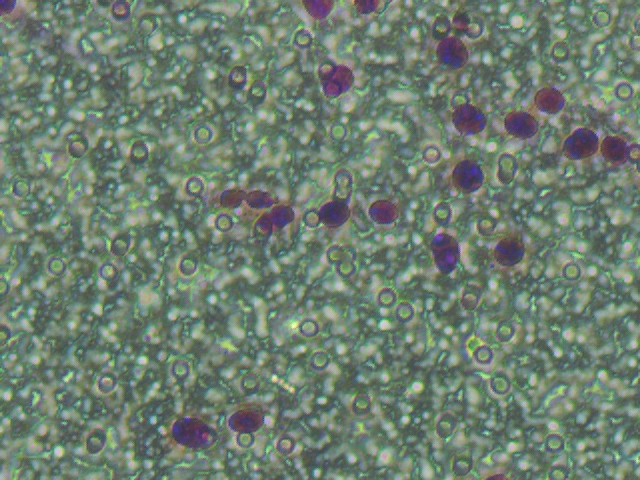

Supplement: S5 File — (ZIP) [file pone.0294566.s005.zip › support information/transwell/sh-PHD2+8um Dorsomorphin/transwell-sh-PHD2+8um Dorsomorphin (1).jpg]

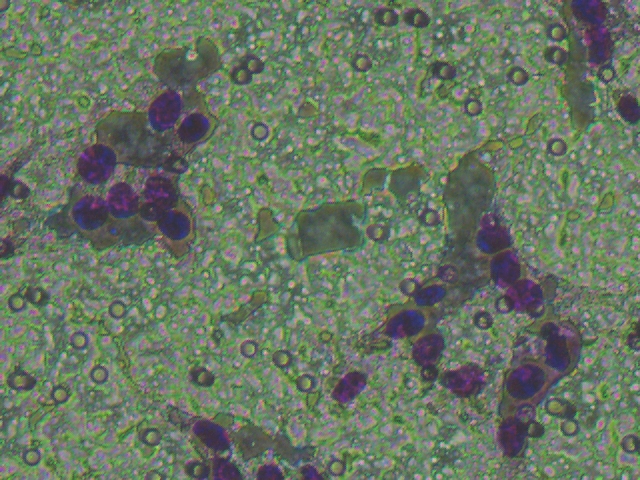

Supplement: S5 File — (ZIP) [file pone.0294566.s005.zip › support information/transwell/sh-PHD2+8um Dorsomorphin/transwell-sh-PHD2+8um Dorsomorphin (2).jpg]

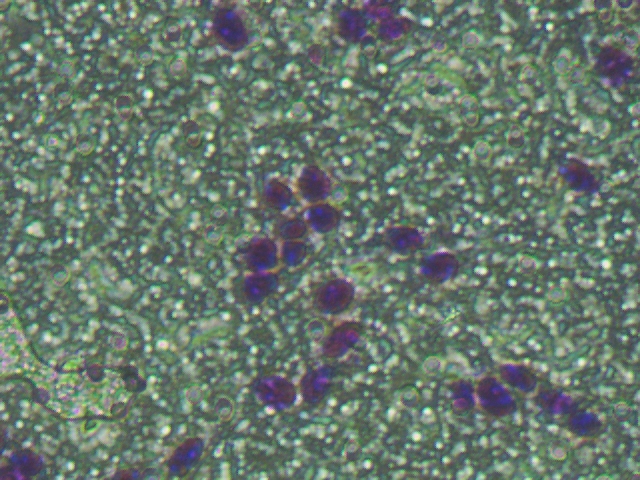

Supplement: S5 File — (ZIP) [file pone.0294566.s005.zip › support information/transwell/sh-PHD2+8um Dorsomorphin/transwell-sh-PHD2+8um Dorsomorphin (3).jpg]

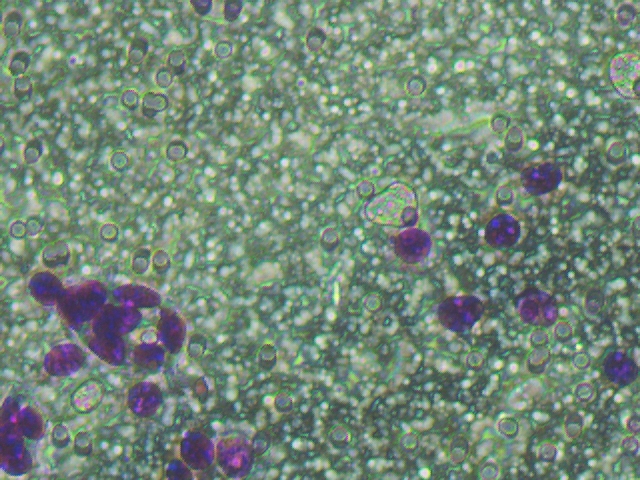

Supplement: S5 File — (ZIP) [file pone.0294566.s005.zip › support information/transwell/sh-PHD2+8um Dorsomorphin/transwell-sh-PHD2+8um Dorsomorphin (4).jpg]

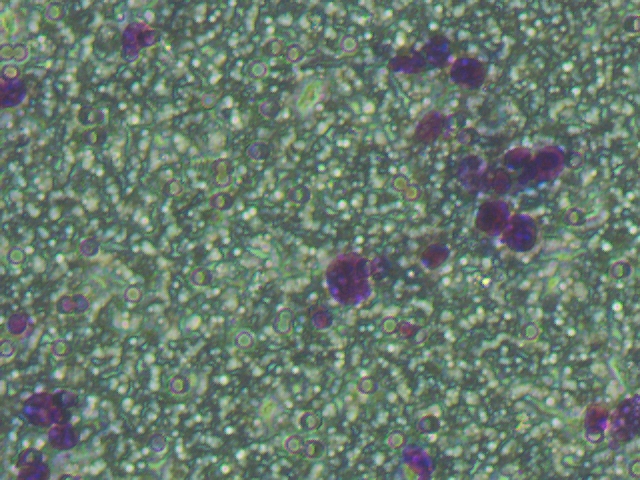

Supplement: S5 File — (ZIP) [file pone.0294566.s005.zip › support information/transwell/sh-PHD2+8um Dorsomorphin/transwell-sh-PHD2+8um Dorsomorphin (5).jpg]

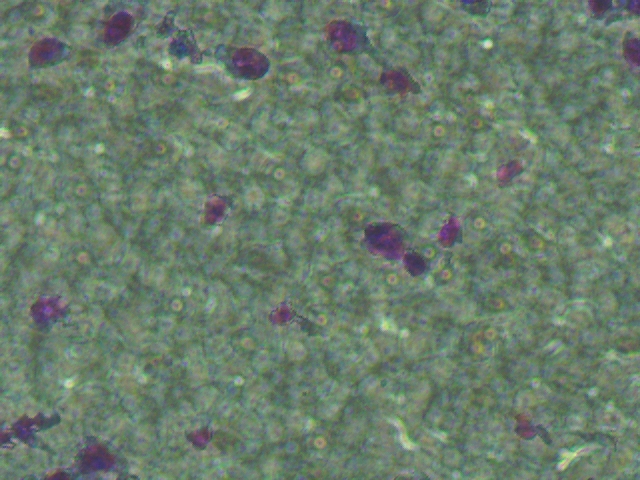

Supplement: S5 File — (ZIP) [file pone.0294566.s005.zip › support information/transwell/sh-PHD2+8um Dorsomorphin/transwell-sh-PHD2+8um Dorsomorphin (6).jpg]

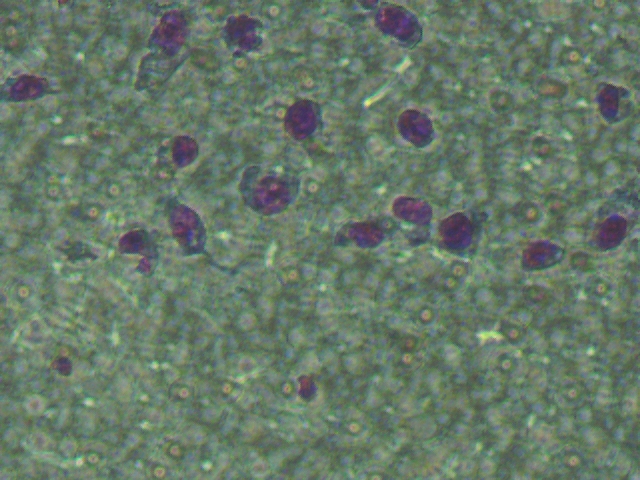

Supplement: S5 File — (ZIP) [file pone.0294566.s005.zip › support information/transwell/sh-PHD2+8um Dorsomorphin/transwell-sh-PHD2+8um Dorsomorphin (7).jpg]

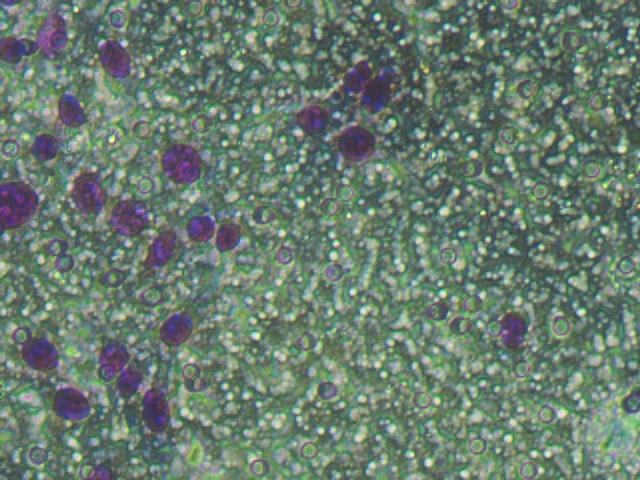

Supplement: S5 File — (ZIP) [file pone.0294566.s005.zip › support information/transwell/sh-PHD2+8um Dorsomorphin/transwell-sh-PHD2+8um Dorsomorphin (8).jpg]

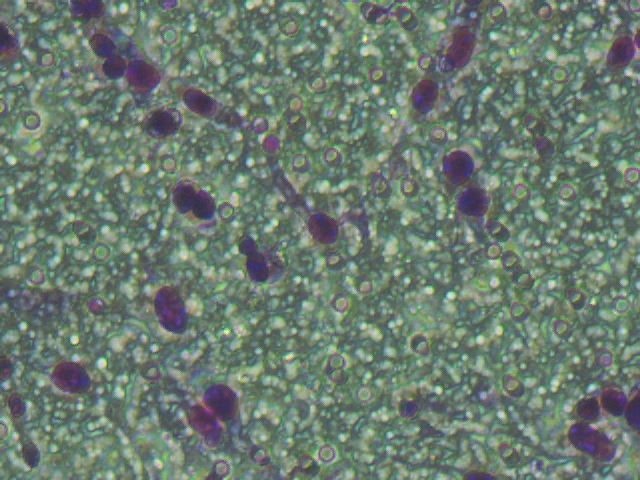

Supplement: S5 File — (ZIP) [file pone.0294566.s005.zip › support information/transwell/sh-PHD2+8um Dorsomorphin/transwell-sh-PHD2+8um Dorsomorphin (9).jpg]

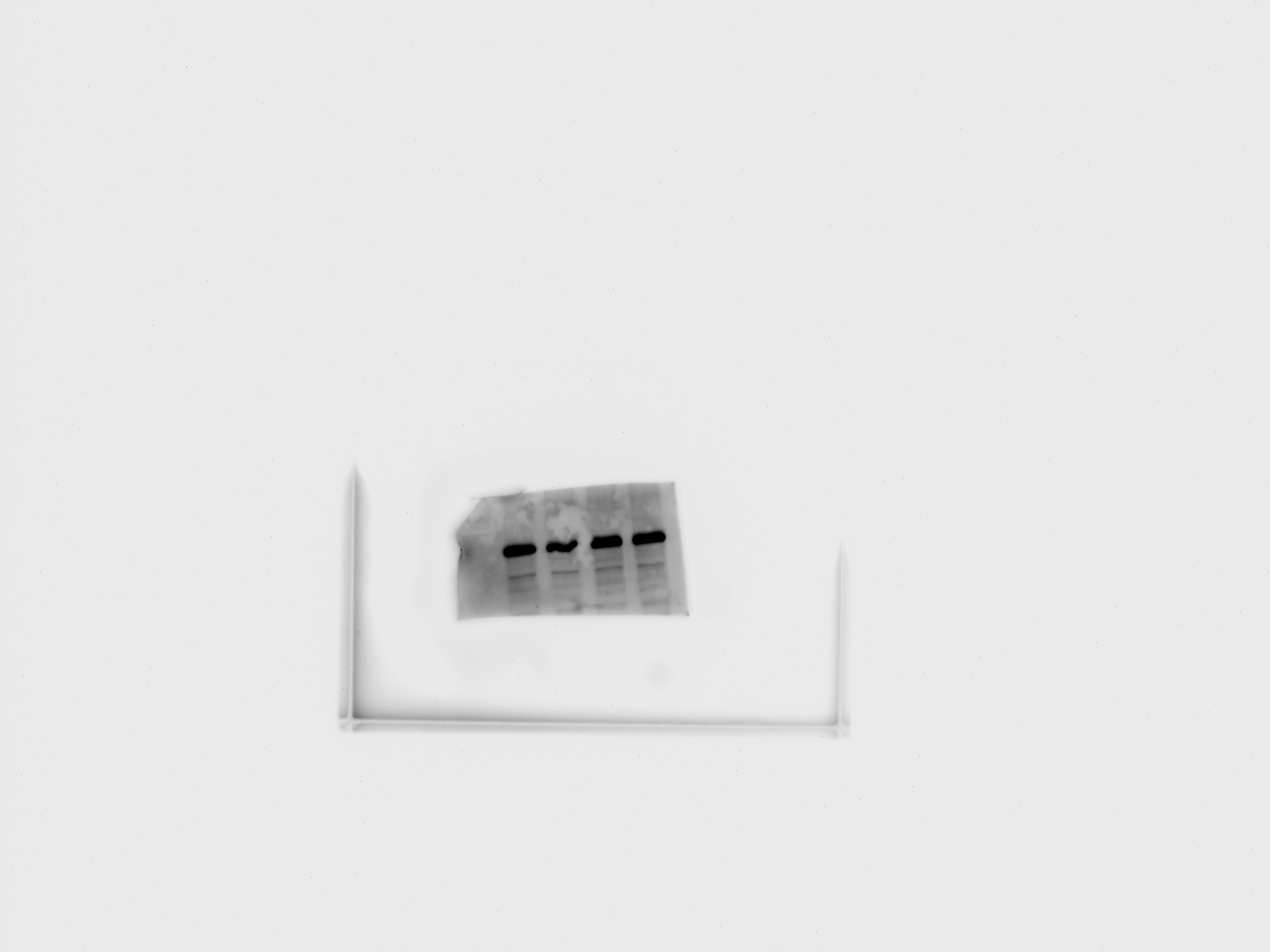

Supplement: S6 File — (ZIP) [file pone.0294566.s006.zip › support information/wb/cell/AMPK/wb-cell-ampk (1).TIF]

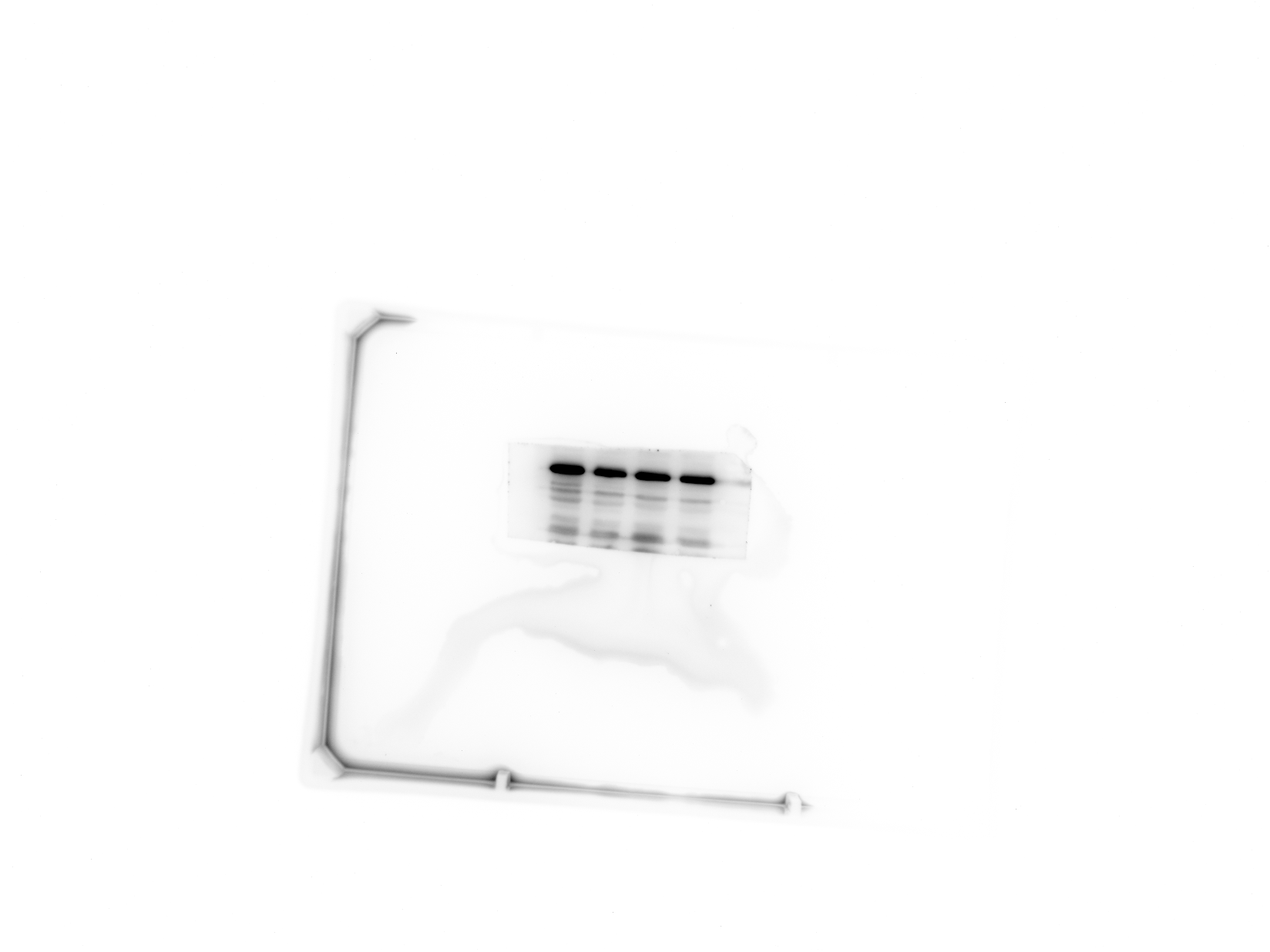

Supplement: S6 File — (ZIP) [file pone.0294566.s006.zip › support information/wb/cell/AMPK/wb-cell-ampk (2).TIF]

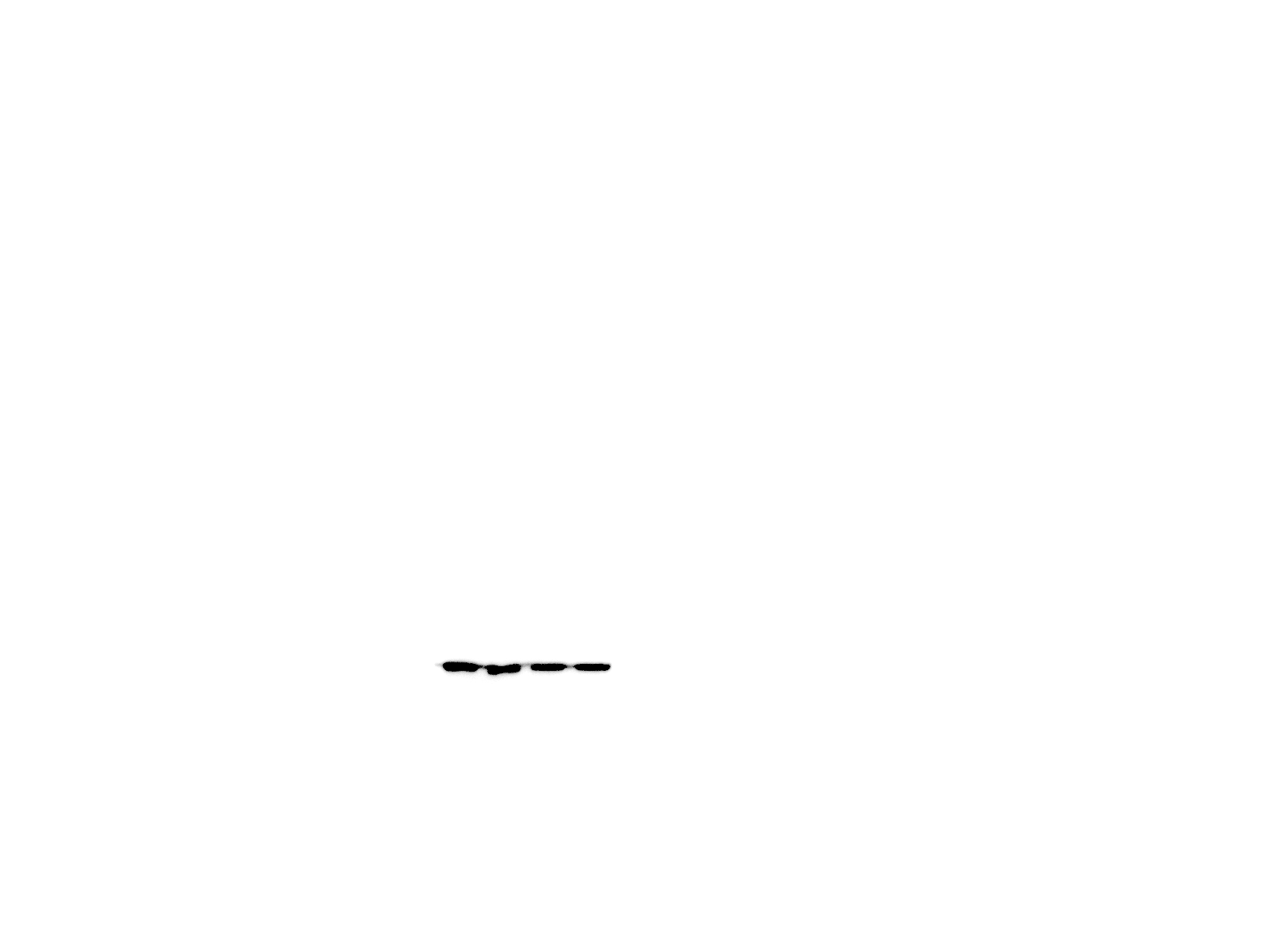

Supplement: S6 File — (ZIP) [file pone.0294566.s006.zip › support information/wb/cell/AMPK/wb-cell-ampk (3).TIF]

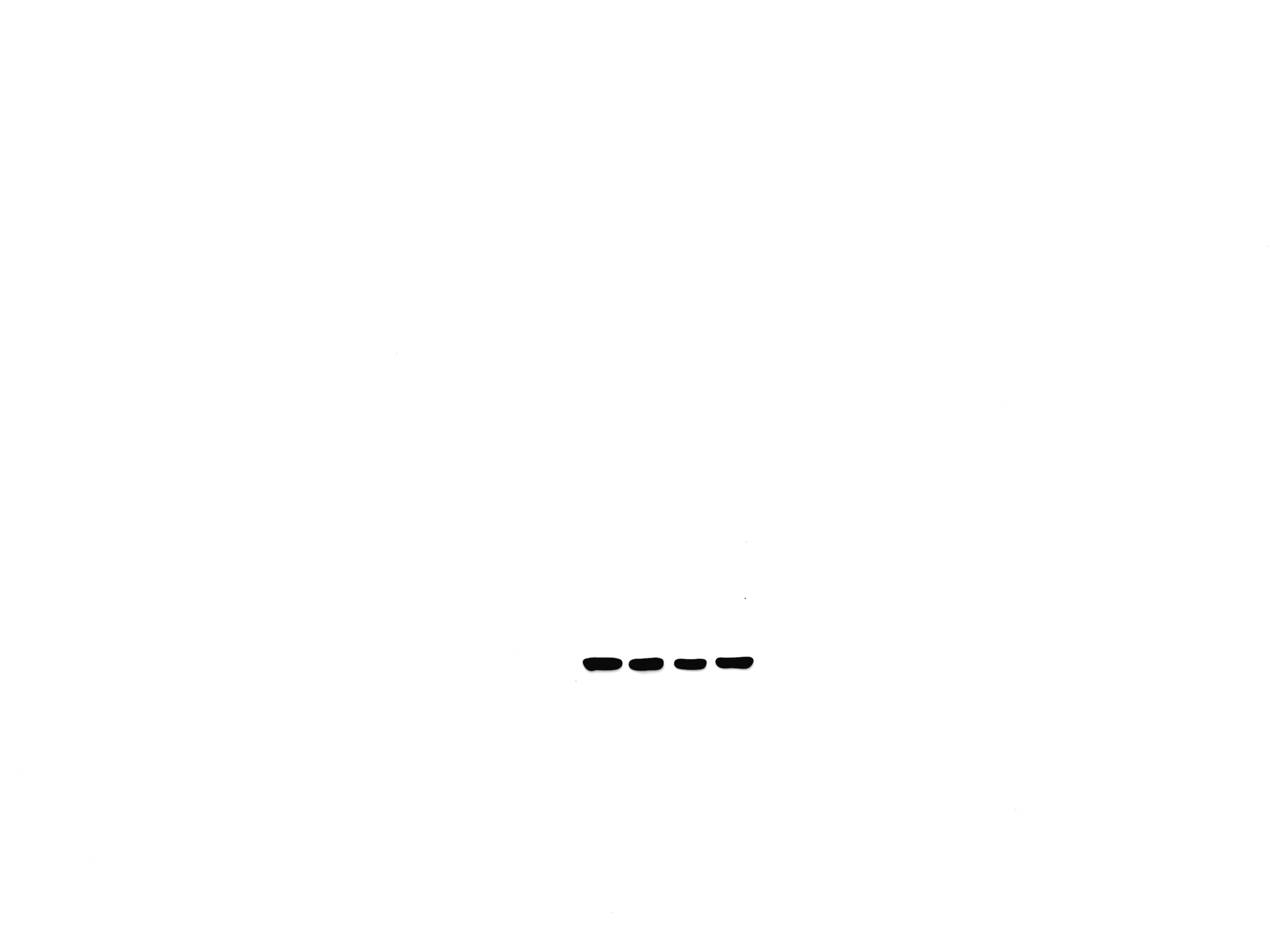

Supplement: S6 File — (ZIP) [file pone.0294566.s006.zip › support information/wb/cell/GAPDH/wb-cell-GAPDH (1).TIF]

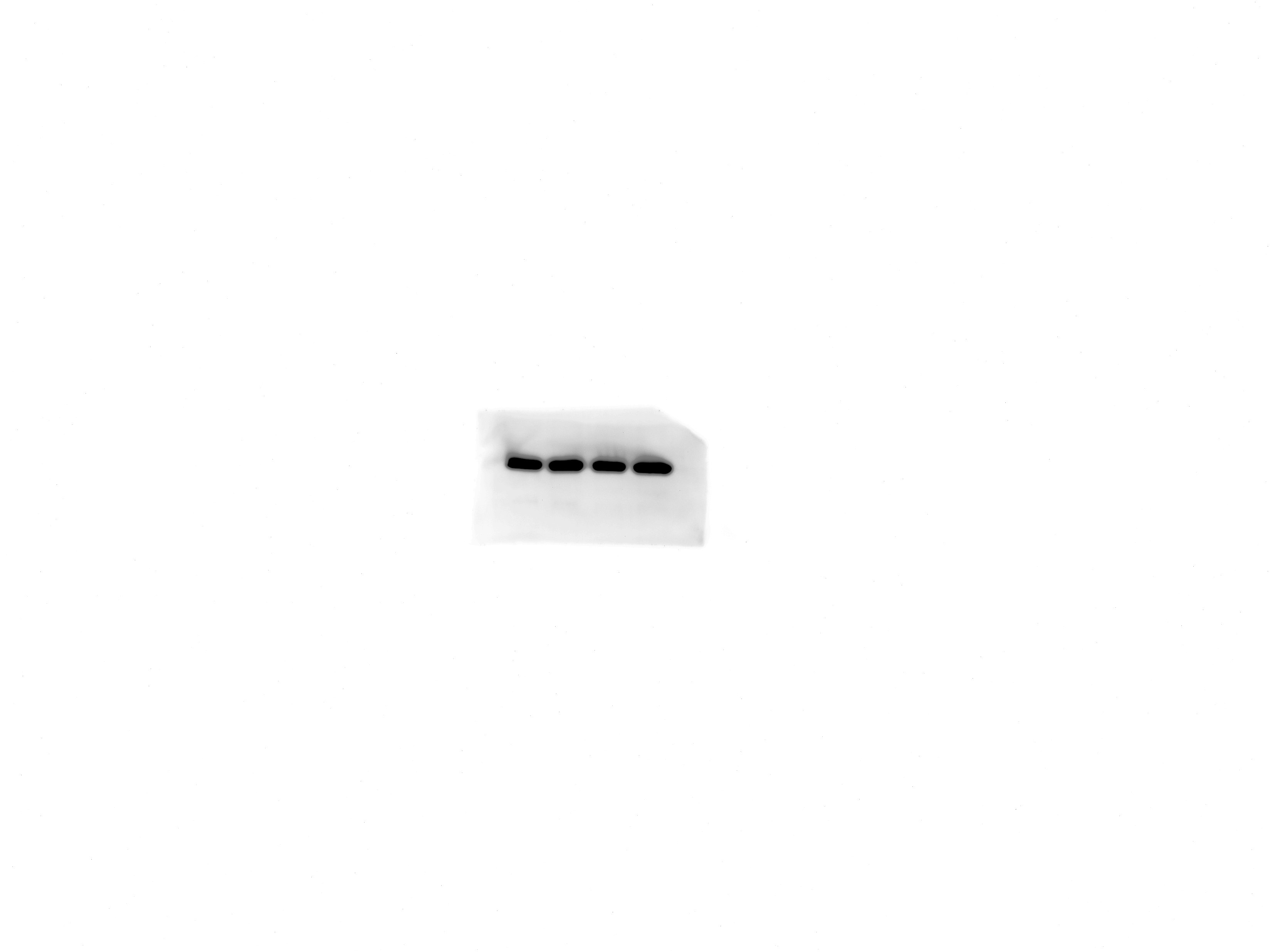

Supplement: S6 File — (ZIP) [file pone.0294566.s006.zip › support information/wb/cell/GAPDH/wb-cell-GAPDH (2).TIF]

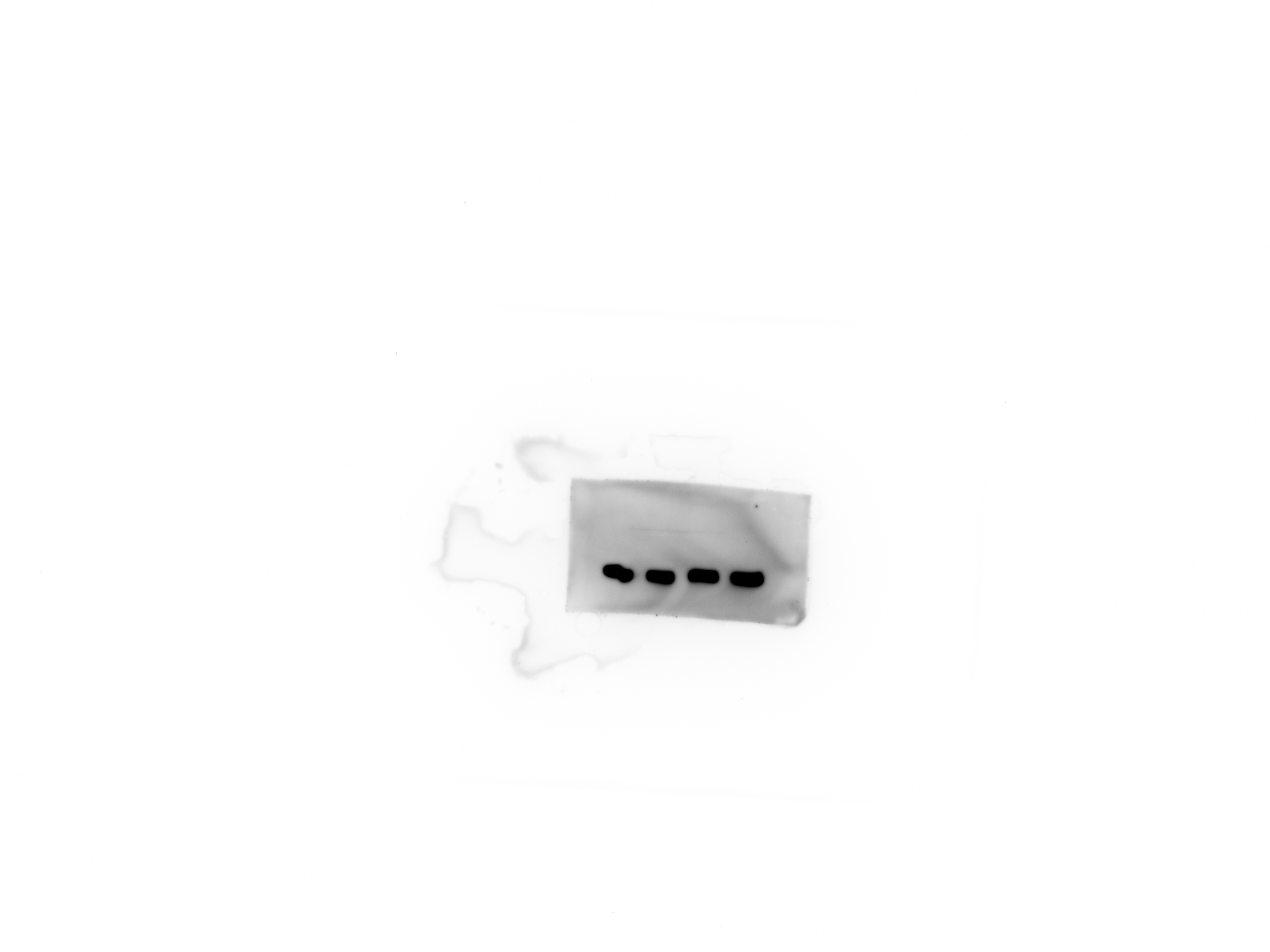

Supplement: S6 File — (ZIP) [file pone.0294566.s006.zip › support information/wb/cell/GAPDH/wb-cell-GAPDH (3).TIF]

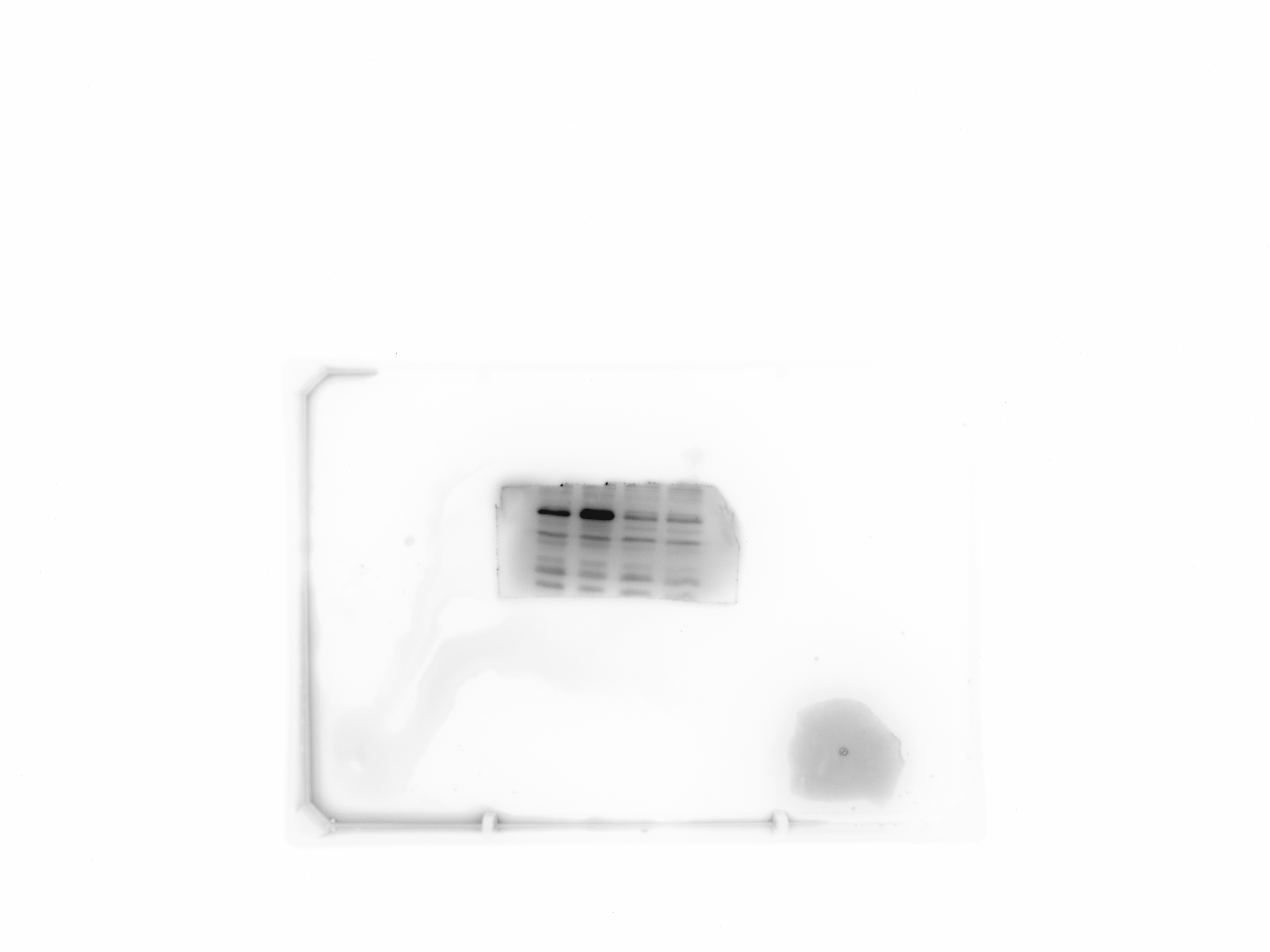

Supplement: S6 File — (ZIP) [file pone.0294566.s006.zip › support information/wb/cell/P-AMPK/wb-cell-p-AMPK (1).TIF]

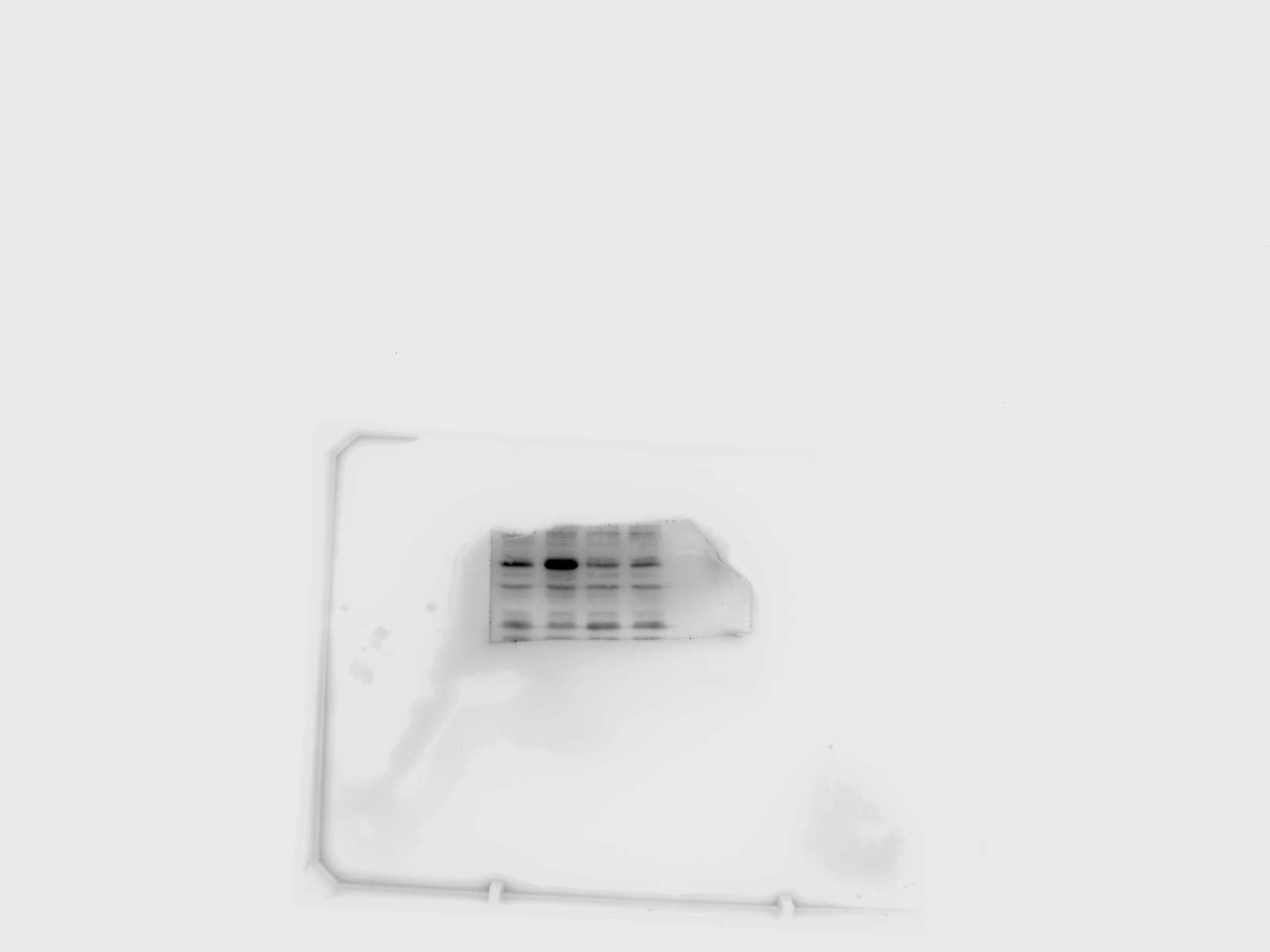

Supplement: S6 File — (ZIP) [file pone.0294566.s006.zip › support information/wb/cell/P-AMPK/wb-cell-p-AMPK (2).TIF]

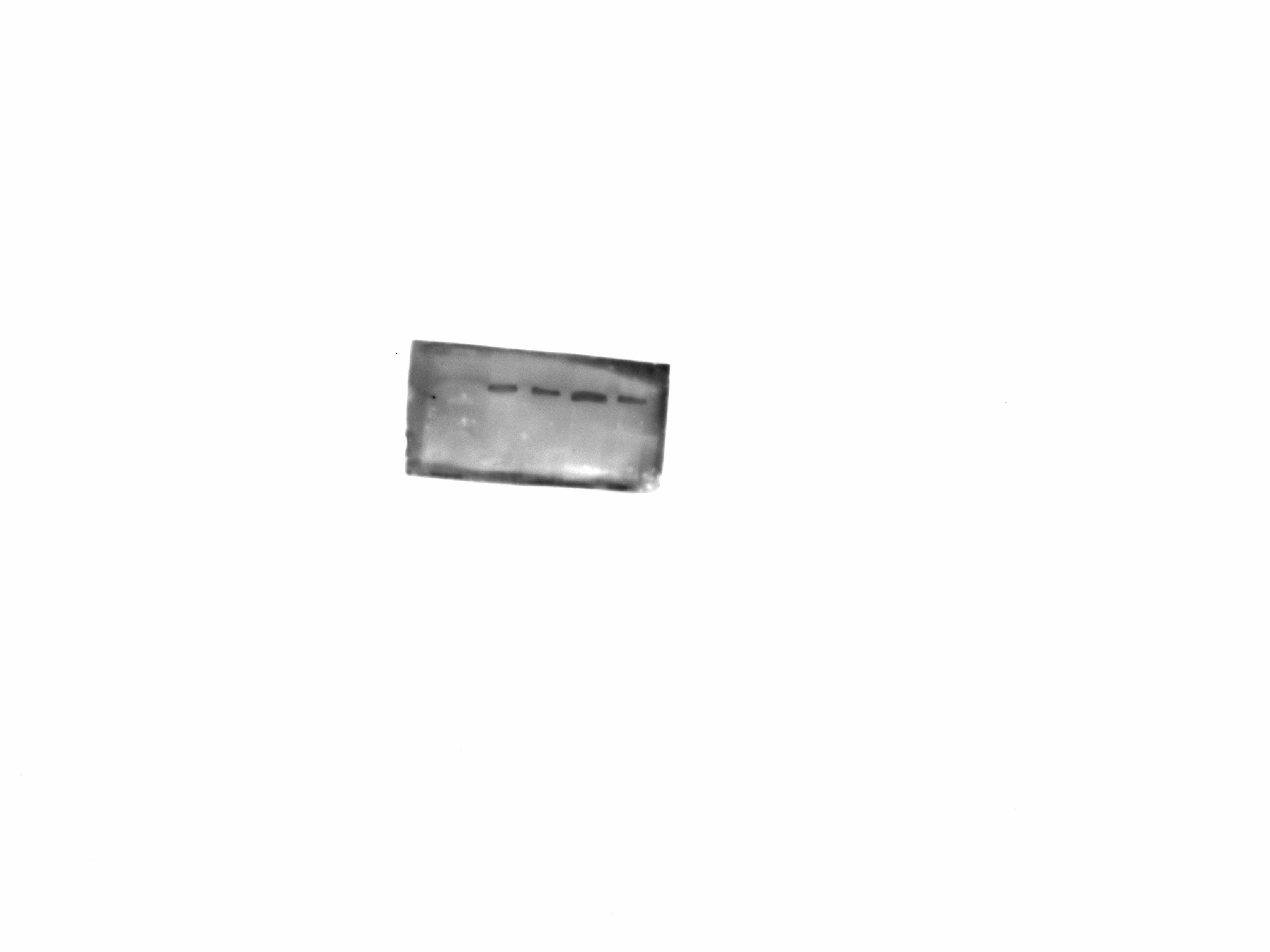

Supplement: S6 File — (ZIP) [file pone.0294566.s006.zip › support information/wb/cell/P-AMPK/wb-cell-p-AMPK (3).TIF]

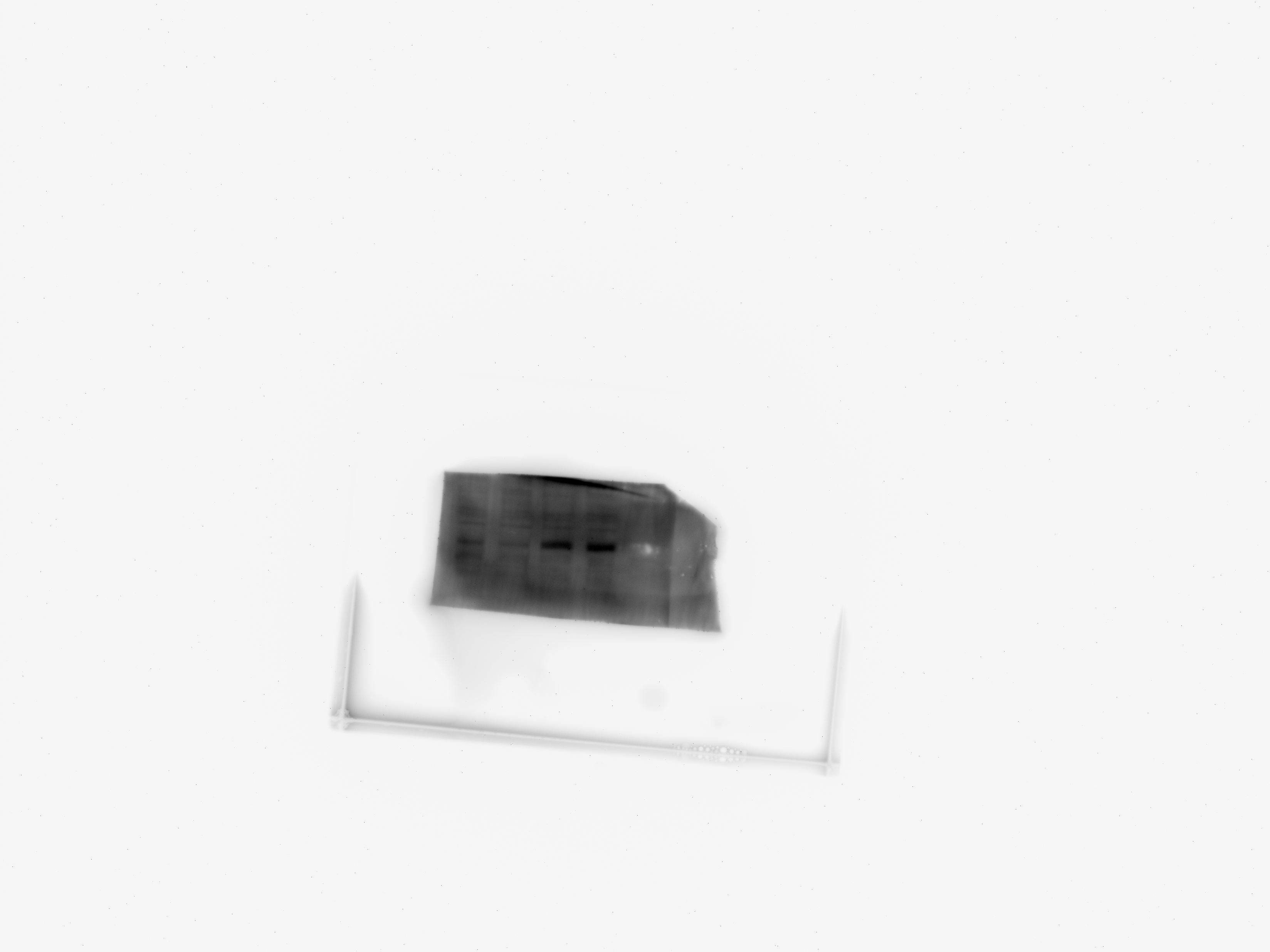

Supplement: S6 File — (ZIP) [file pone.0294566.s006.zip › support information/wb/cell/PHD2/wb-cell-PHD2 (1).TIF]

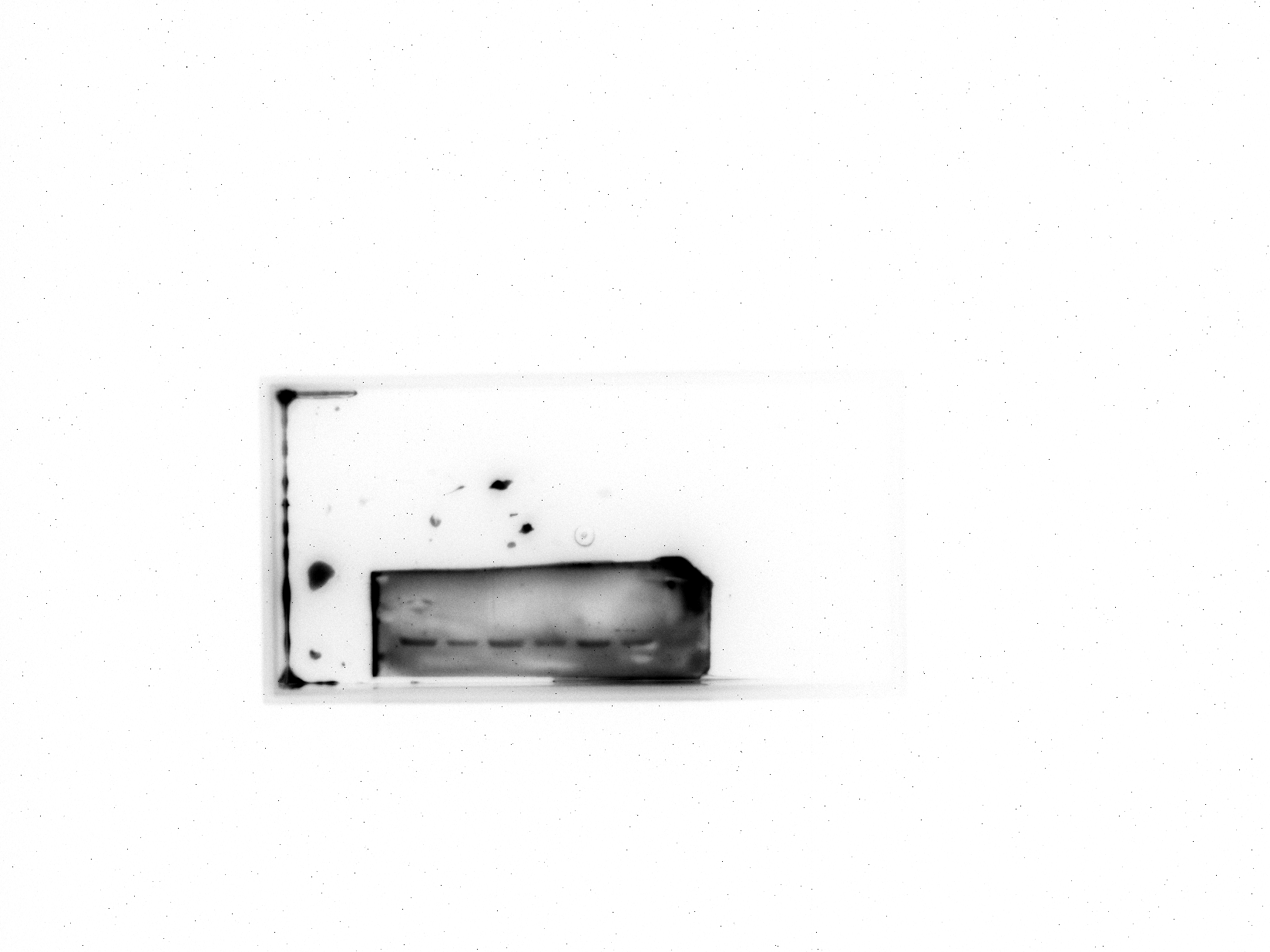

Supplement: S6 File — (ZIP) [file pone.0294566.s006.zip › support information/wb/tissue/FGF-2/wb-tissue-FGF-2.TIF]

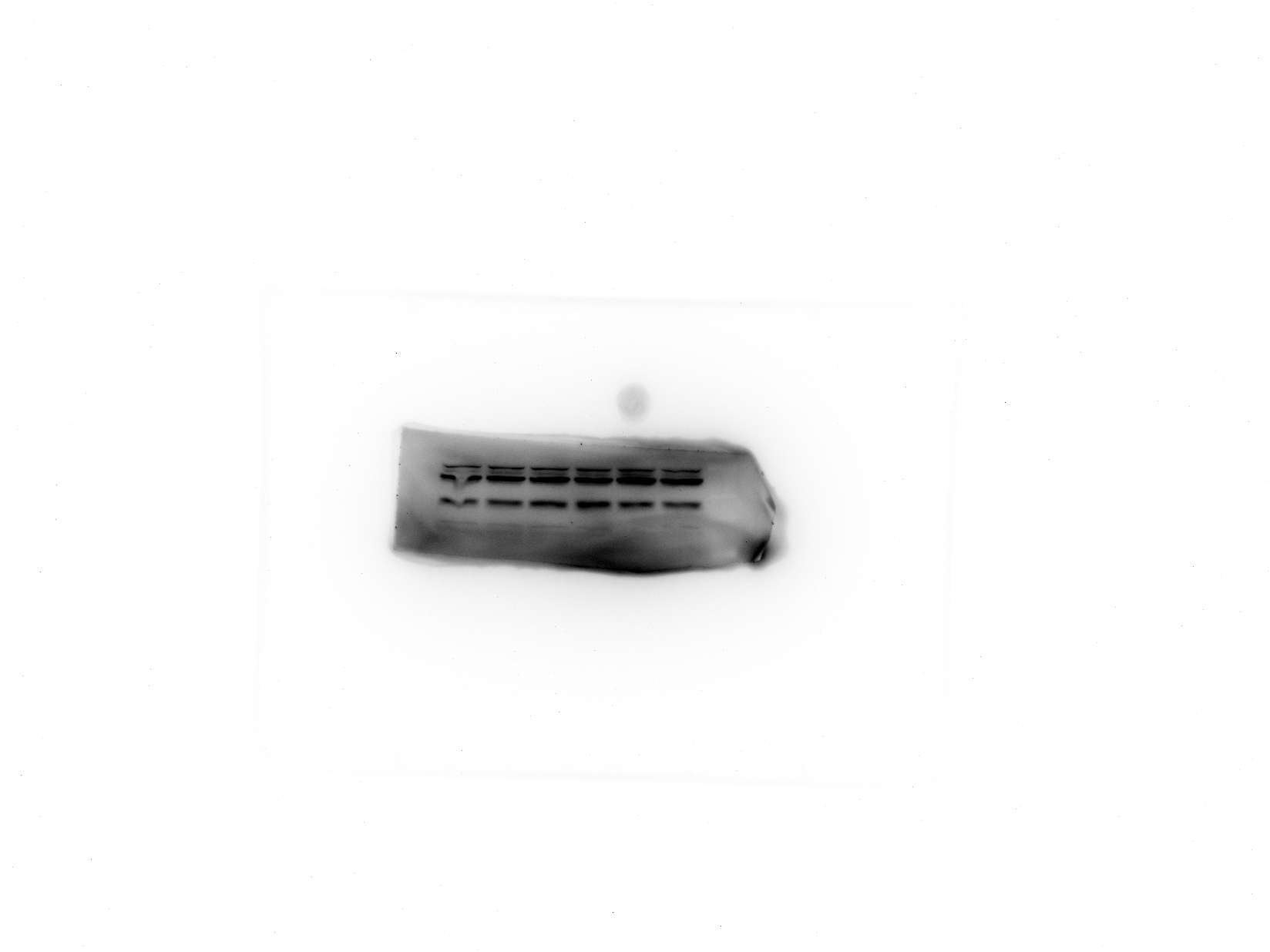

Supplement: S6 File — (ZIP) [file pone.0294566.s006.zip › support information/wb/tissue/GAPDH/wb-tissue-GAPDH (1).TIF]

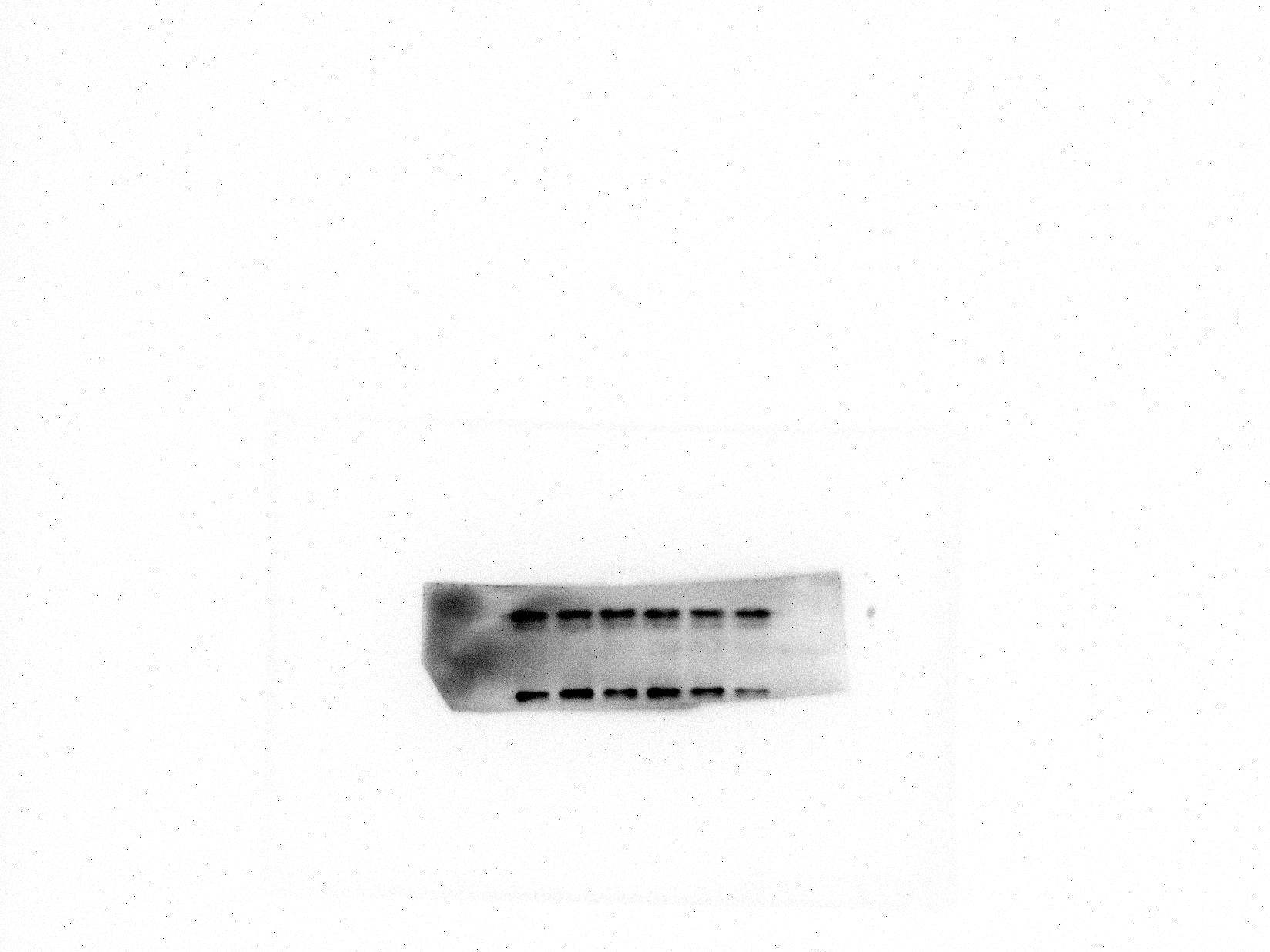

Supplement: S6 File — (ZIP) [file pone.0294566.s006.zip › support information/wb/tissue/GAPDH/wb-tissue-GAPDH (2).TIF]

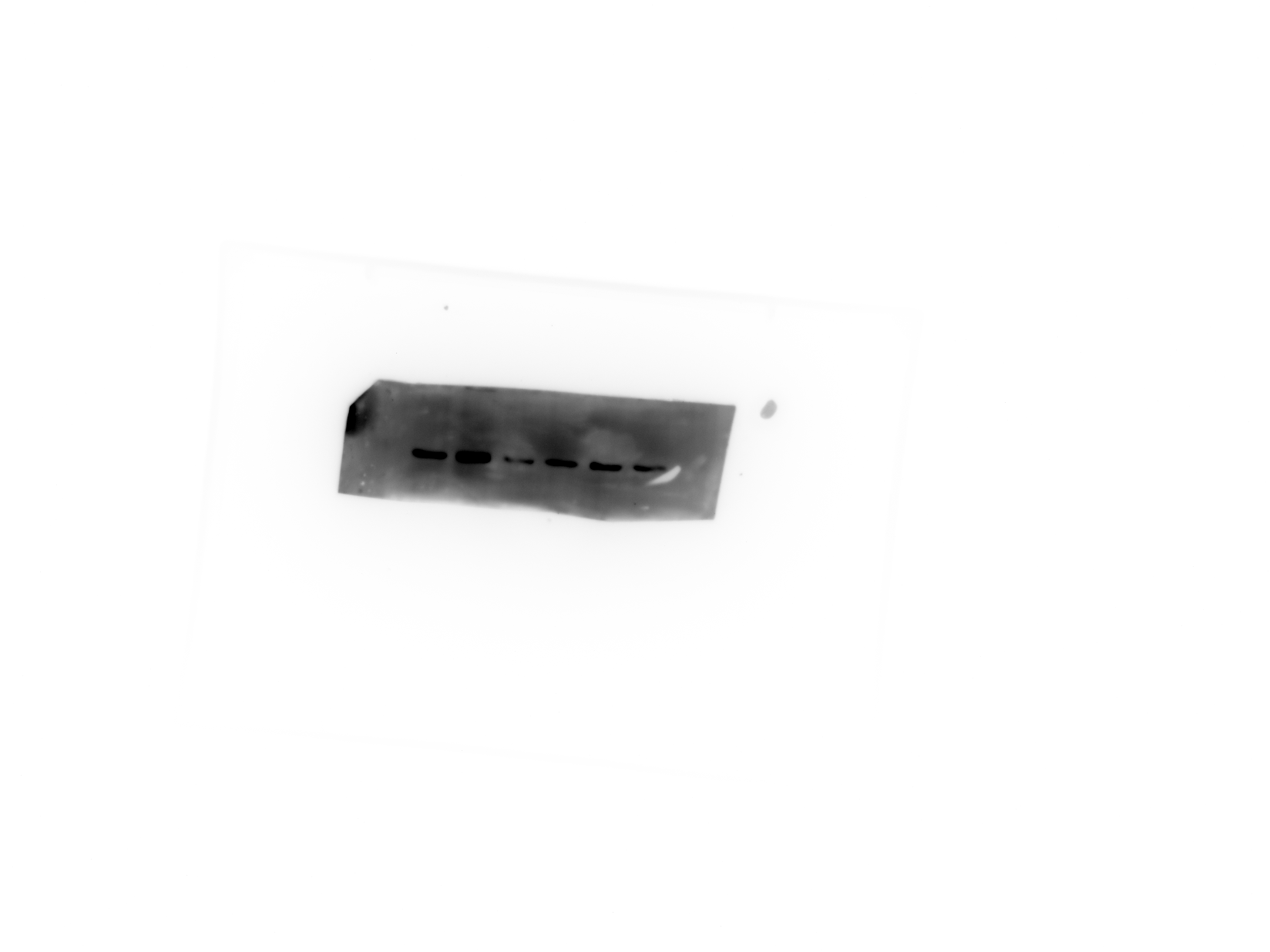

Supplement: S6 File — (ZIP) [file pone.0294566.s006.zip › support information/wb/tissue/PHD2/wb-tissue-PHD2 (1).TIF]

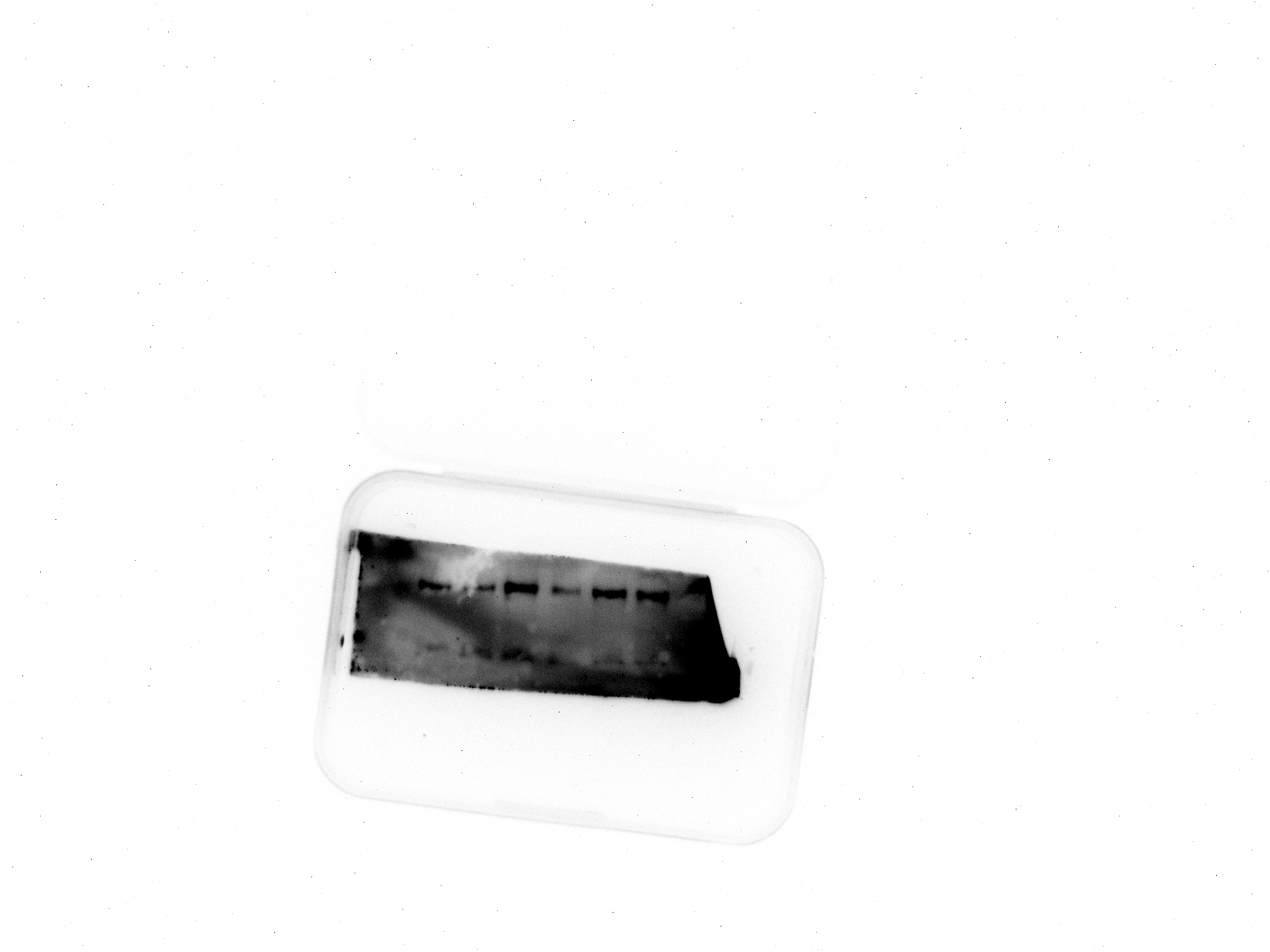

Supplement: S6 File — (ZIP) [file pone.0294566.s006.zip › support information/wb/tissue/PHD2/wb-tissue-PHD2 (2).TIF]

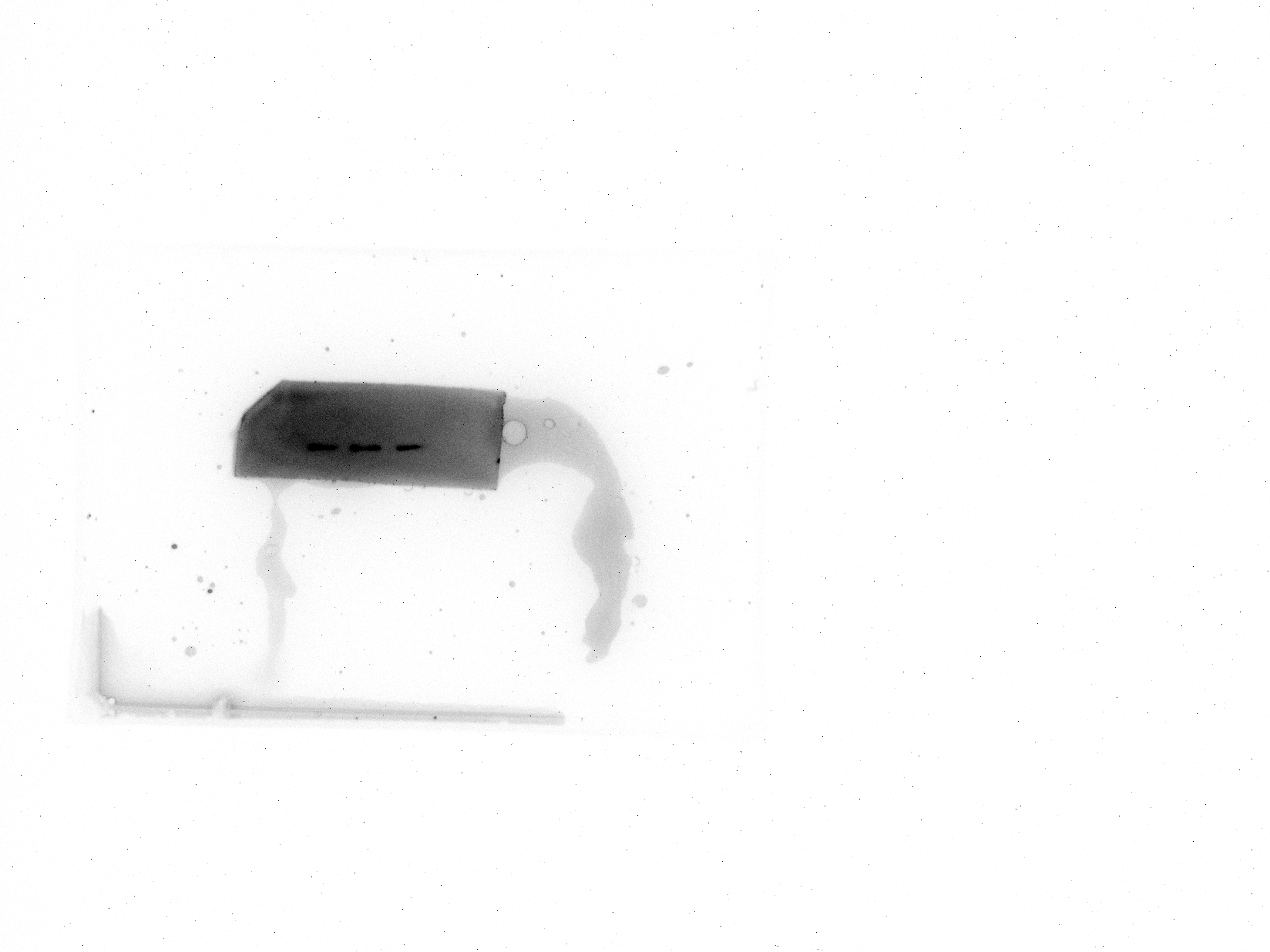

Supplement: S6 File — (ZIP) [file pone.0294566.s006.zip › support information/wb/tissue/VEGF/wb-tissue-VEGF (1).TIF]

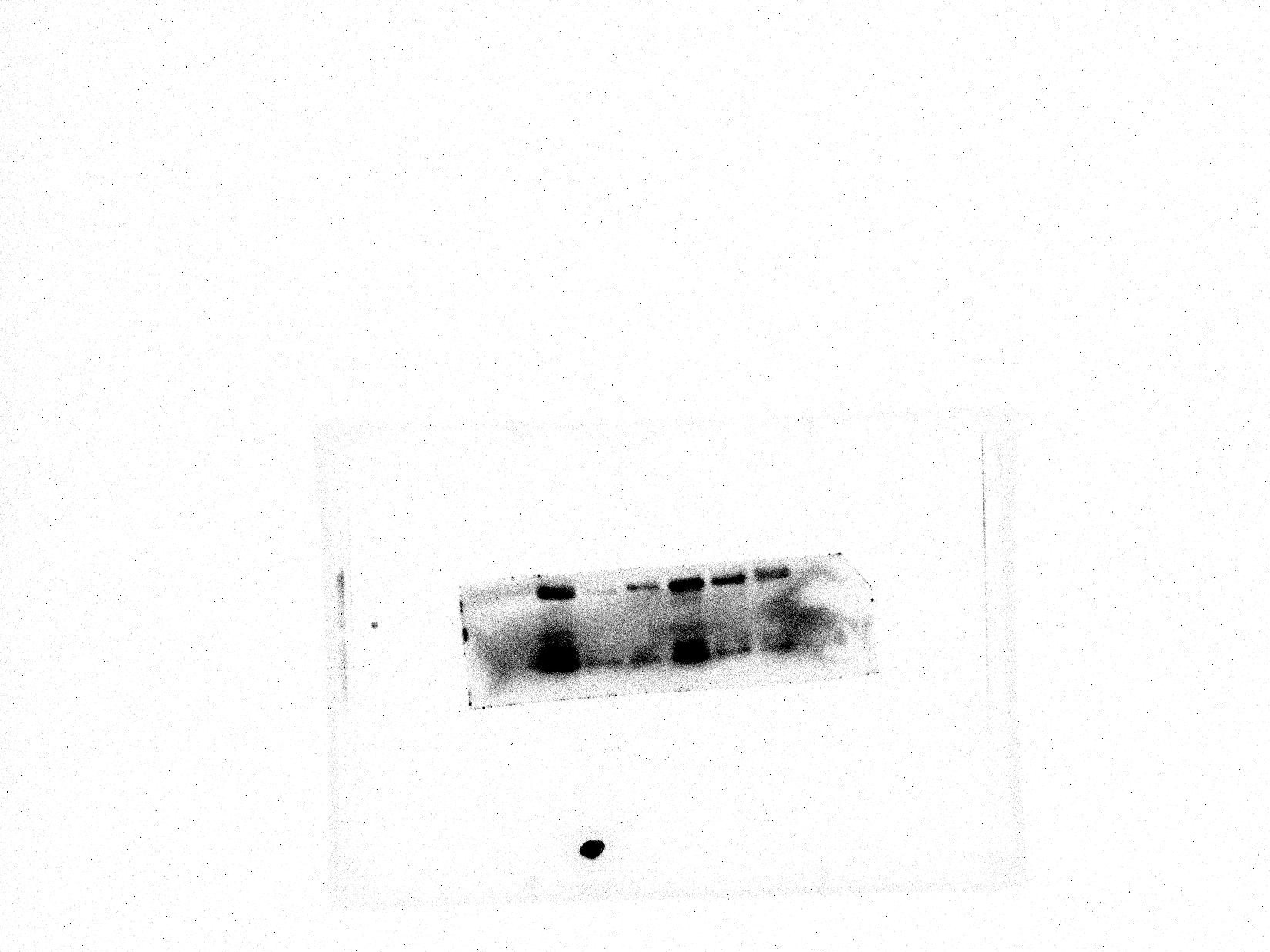

Supplement: S6 File — (ZIP) [file pone.0294566.s006.zip › support information/wb/tissue/VEGF/wb-tissue-VEGF (2).TIF]

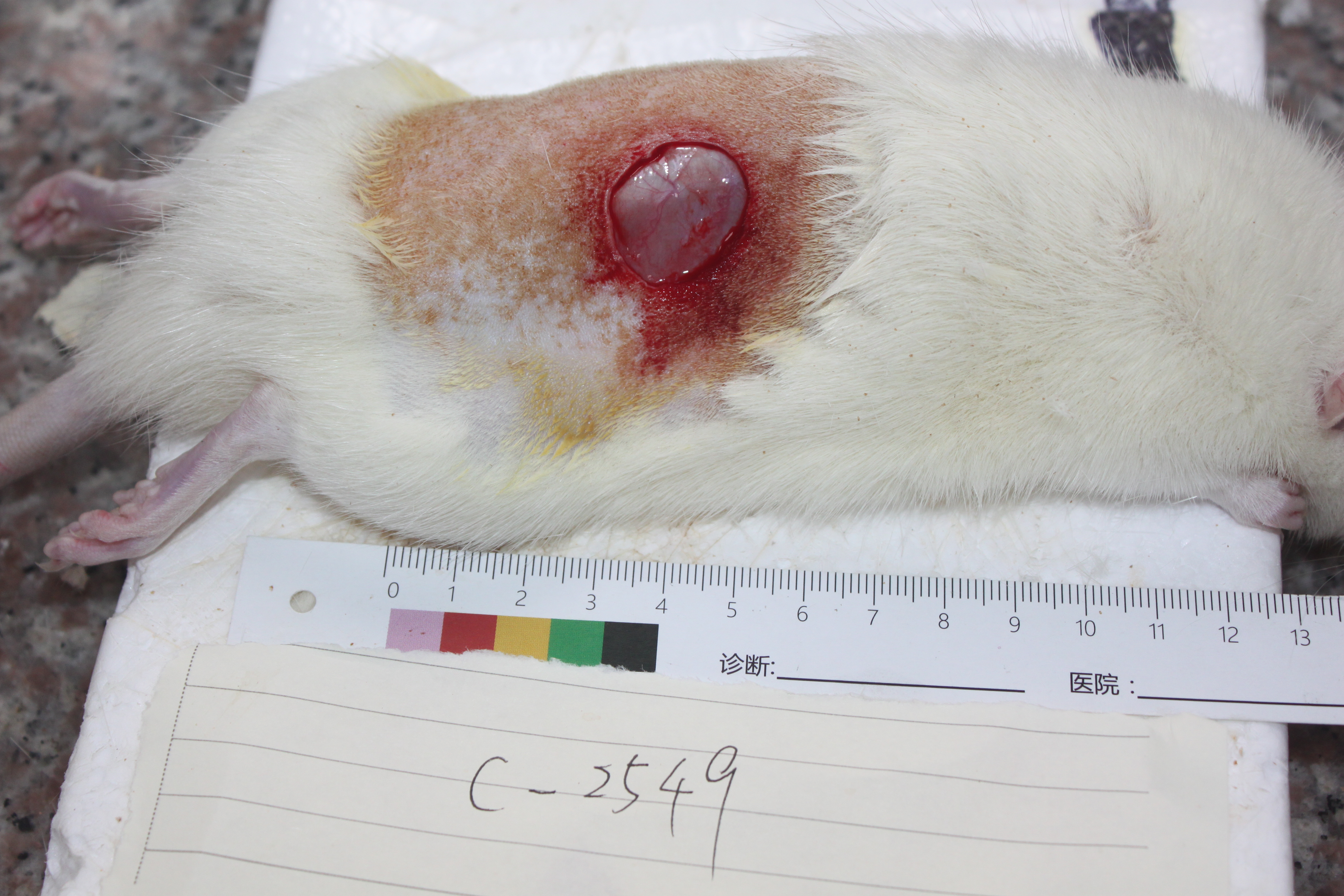

Supplement: S7 File — (ZIP) [file pone.0294566.s007.zip › support information/Wound healing rateú¿day 0 3 7ú⌐/day 0/sh-Control/Wound healing rate-day0-sh-Control (1).JPG]

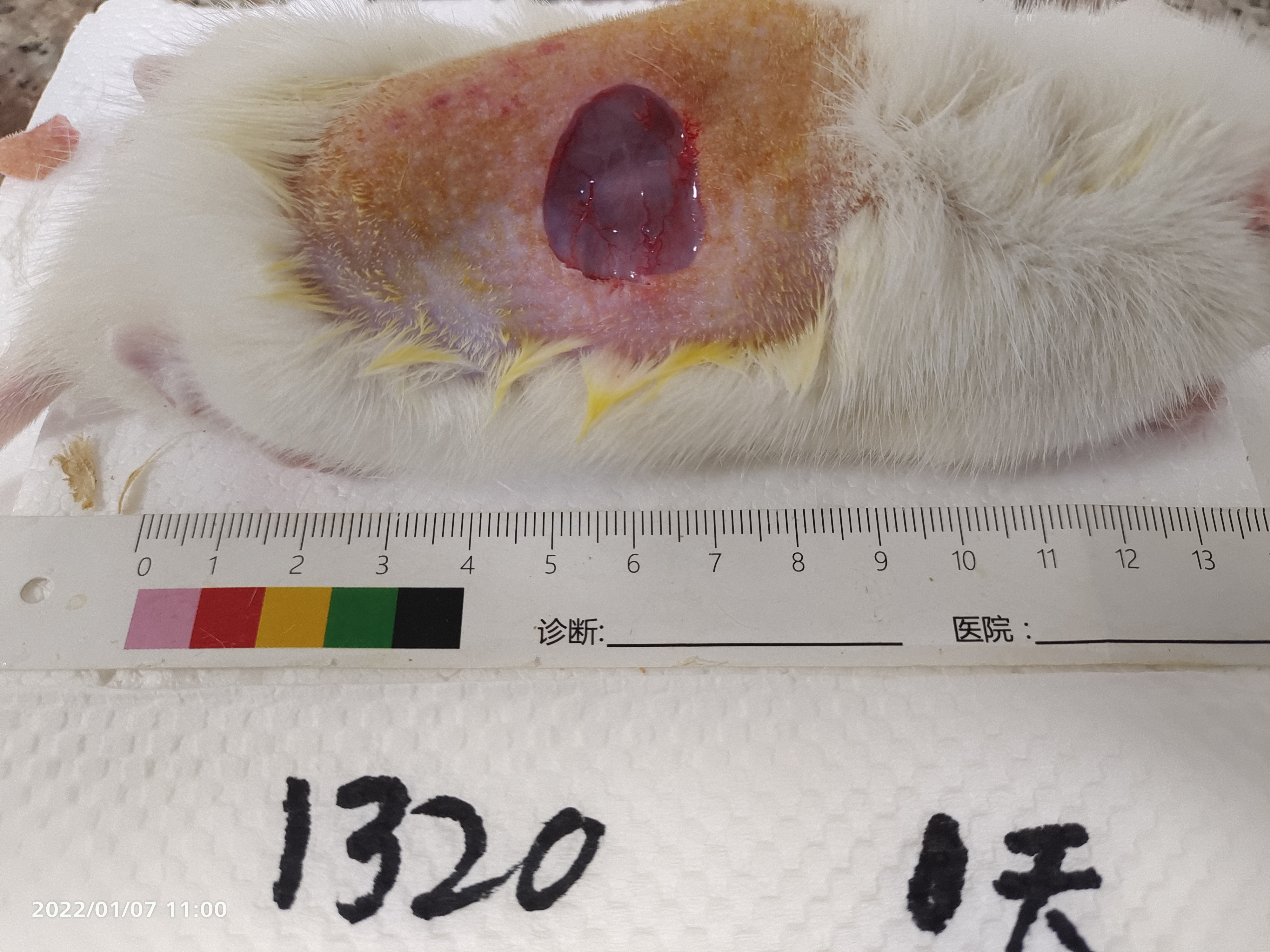

Supplement: S7 File — (ZIP) [file pone.0294566.s007.zip › support information/Wound healing rateú¿day 0 3 7ú⌐/day 0/sh-Control/Wound healing rate-day0-sh-Control (2).jpg]

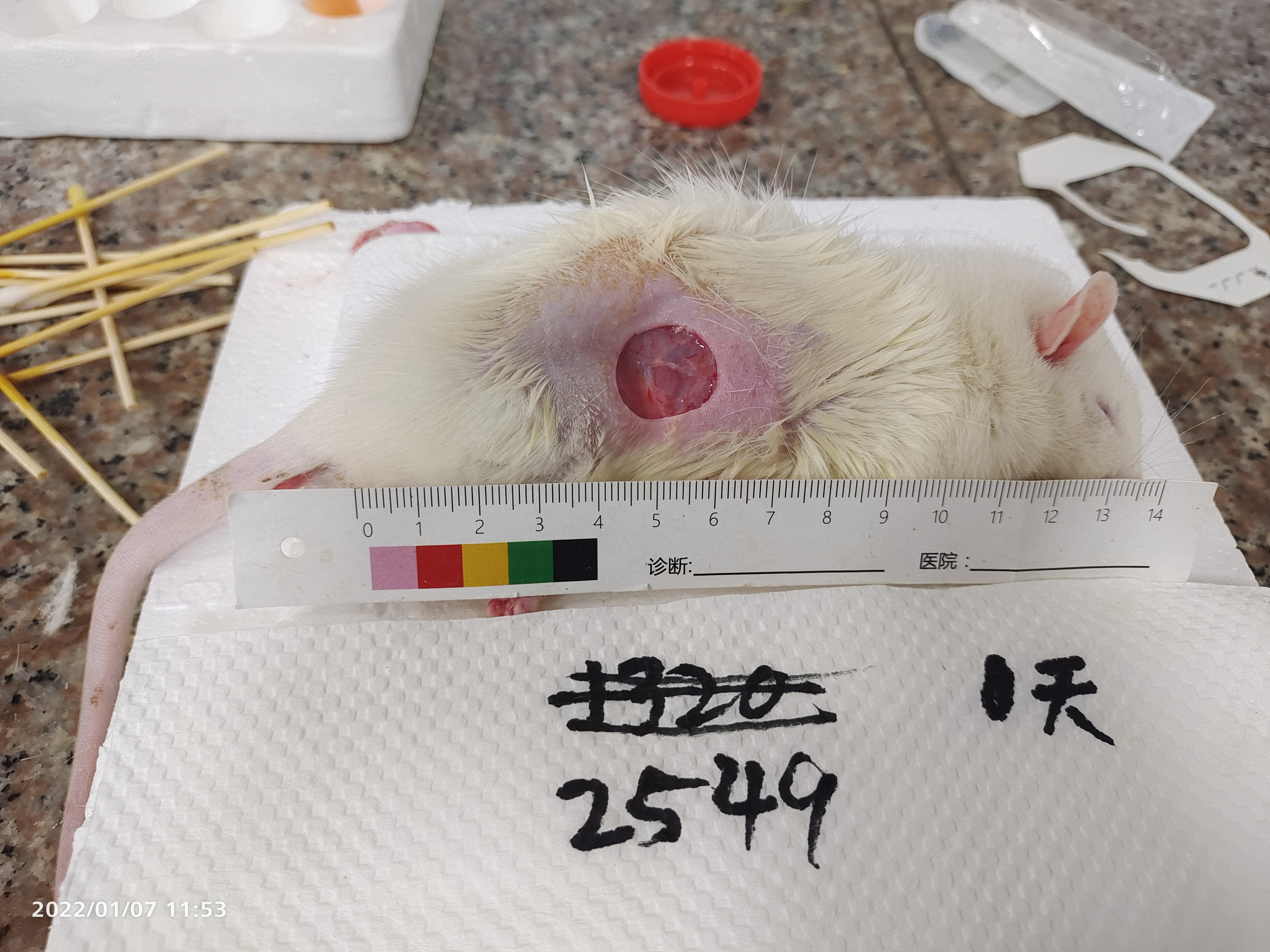

Supplement: S7 File — (ZIP) [file pone.0294566.s007.zip › support information/Wound healing rateú¿day 0 3 7ú⌐/day 0/sh-Control/Wound healing rate-day0-sh-Control (3).jpg]

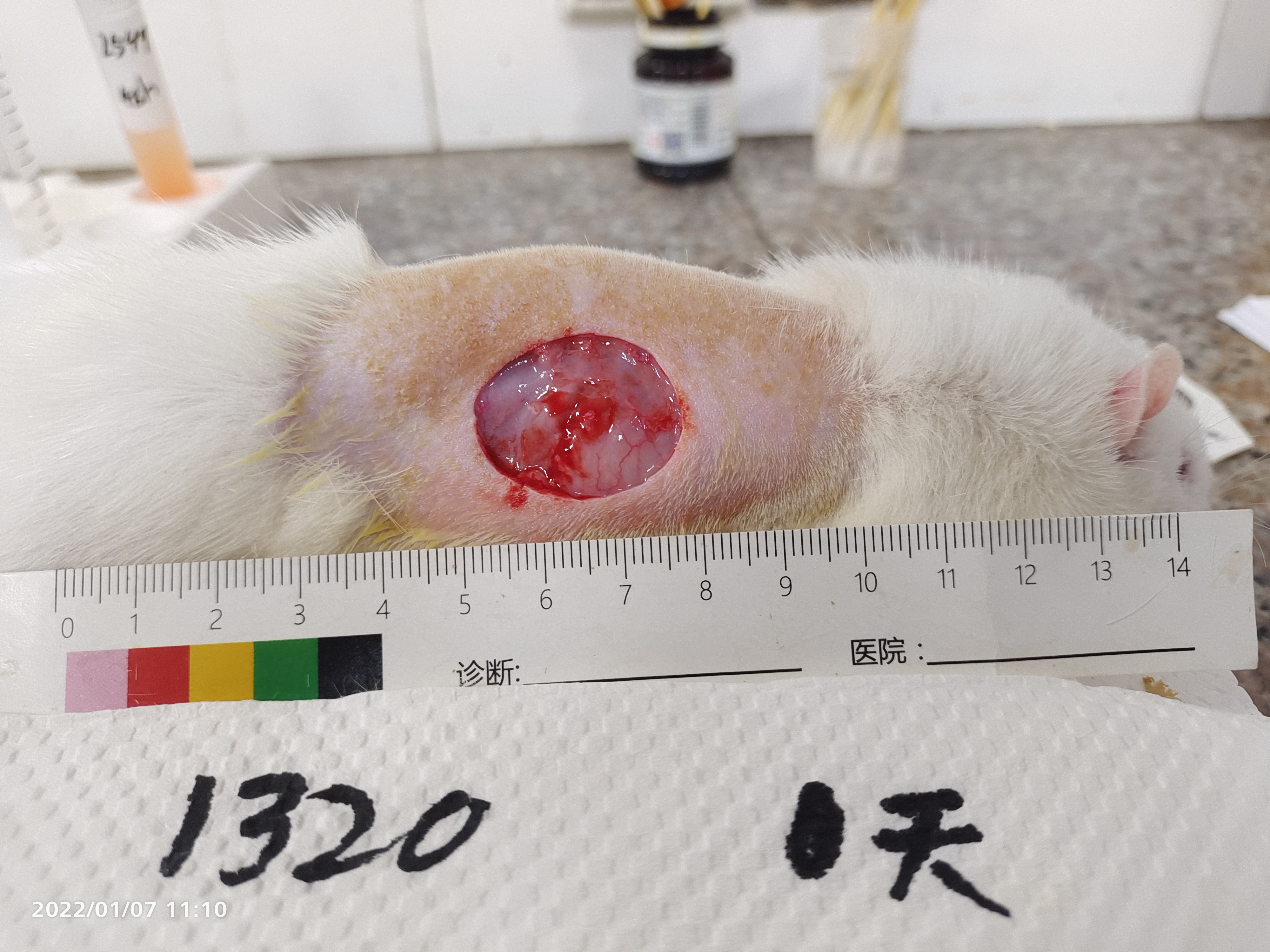

Supplement: S7 File — (ZIP) [file pone.0294566.s007.zip › support information/Wound healing rateú¿day 0 3 7ú⌐/day 0/sh-PHD2/Wound healing rate-day0-sh-PHD2 (1).jpg]

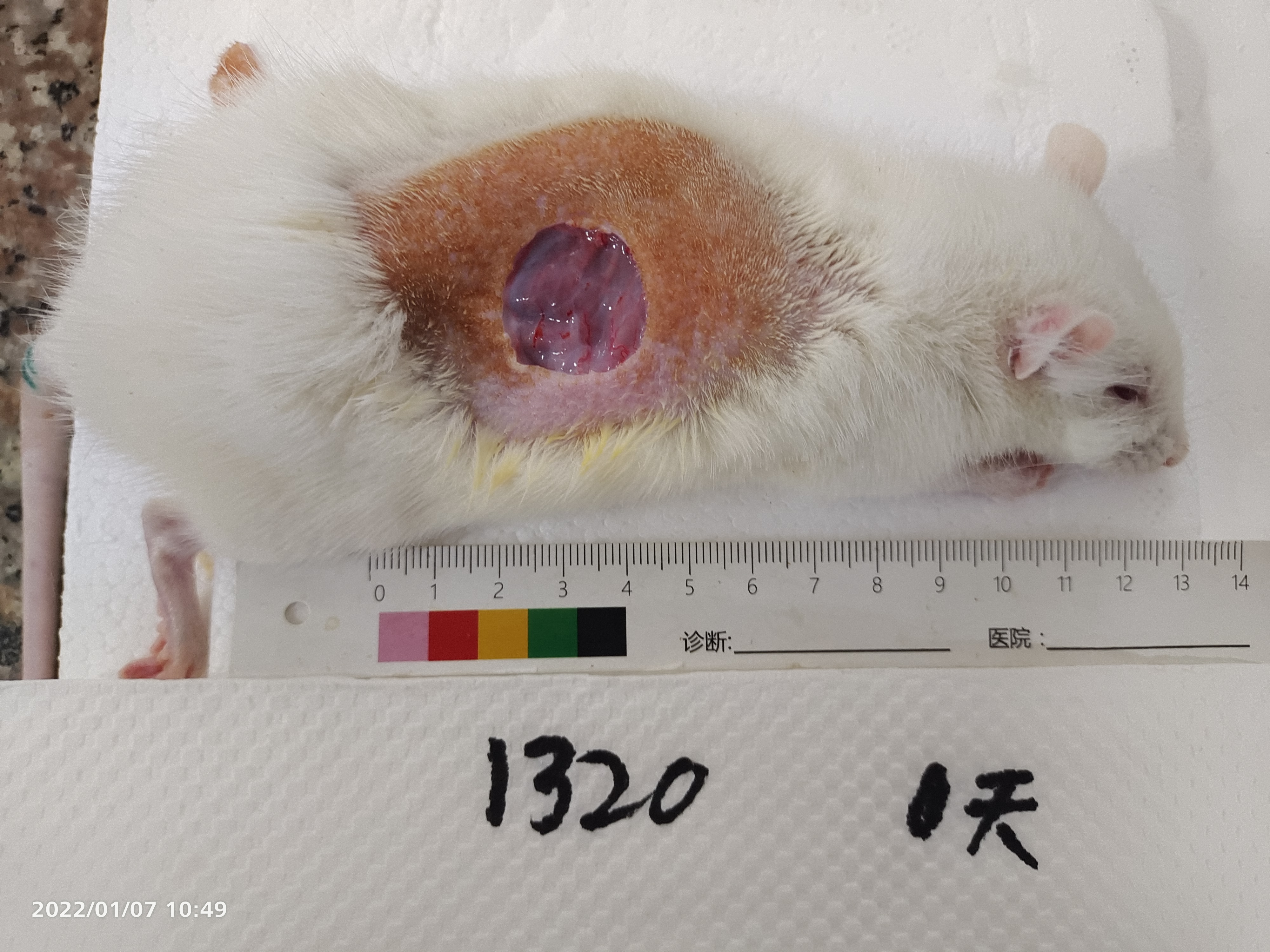

Supplement: S7 File — (ZIP) [file pone.0294566.s007.zip › support information/Wound healing rateú¿day 0 3 7ú⌐/day 0/sh-PHD2/Wound healing rate-day0-sh-PHD2 (2).jpg]

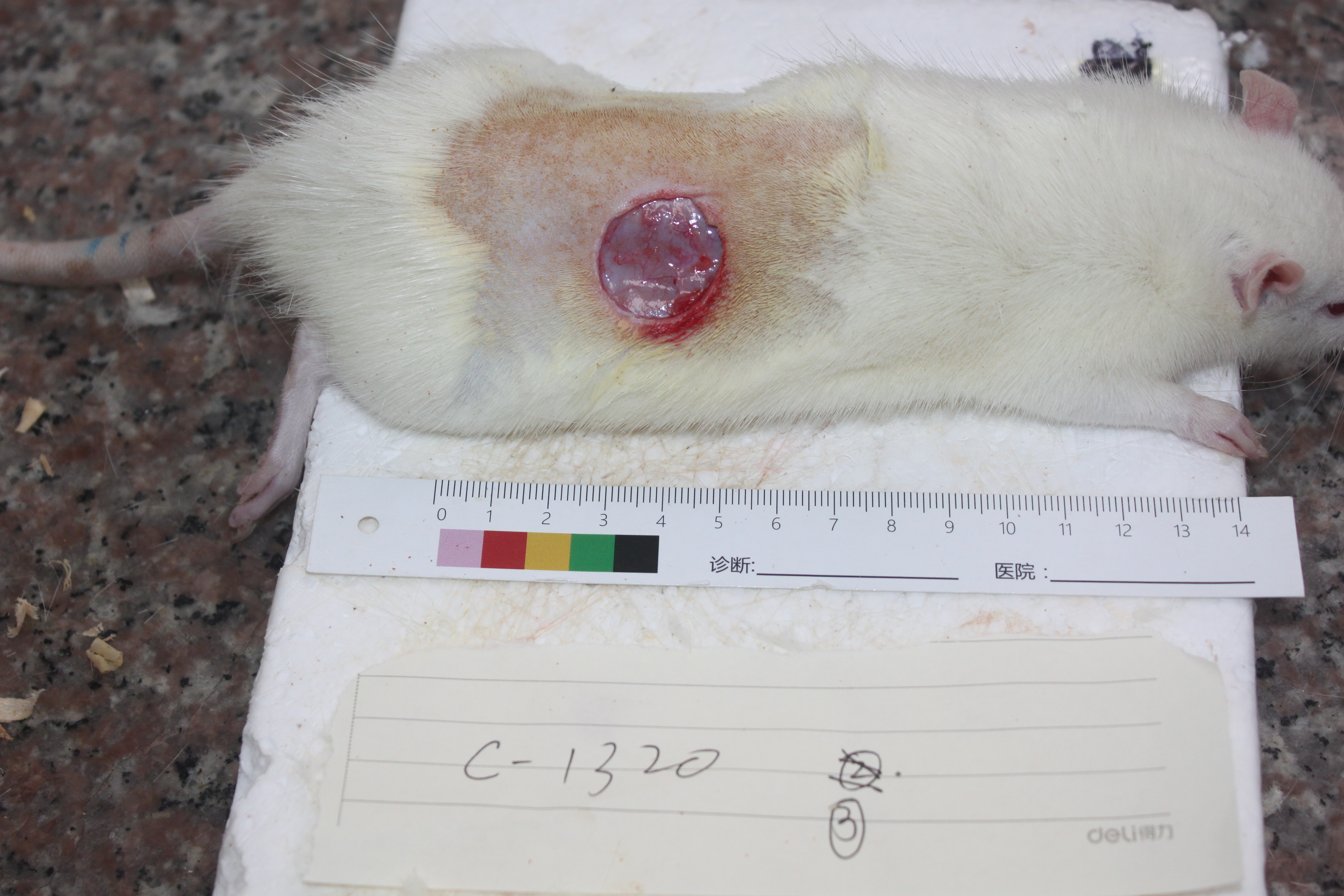

Supplement: S7 File — (ZIP) [file pone.0294566.s007.zip › support information/Wound healing rateú¿day 0 3 7ú⌐/day 0/sh-PHD2/Wound healing rate-day0-sh-PHD2 (3).JPG]

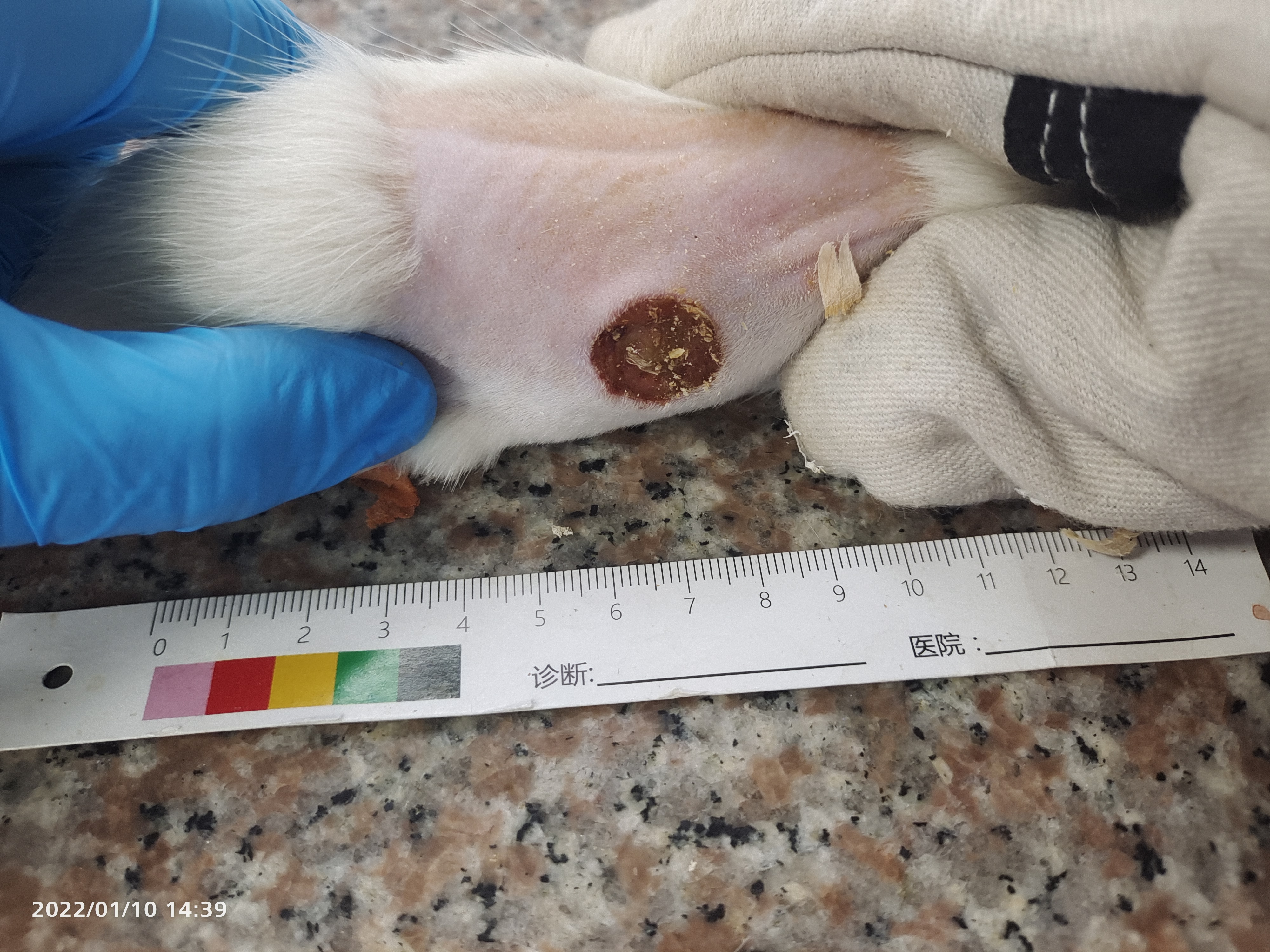

Supplement: S7 File — (ZIP) [file pone.0294566.s007.zip › support information/Wound healing rateú¿day 0 3 7ú⌐/day 3/sh-Control/Wound healing rate-day3-sh-Control (1).jpg]

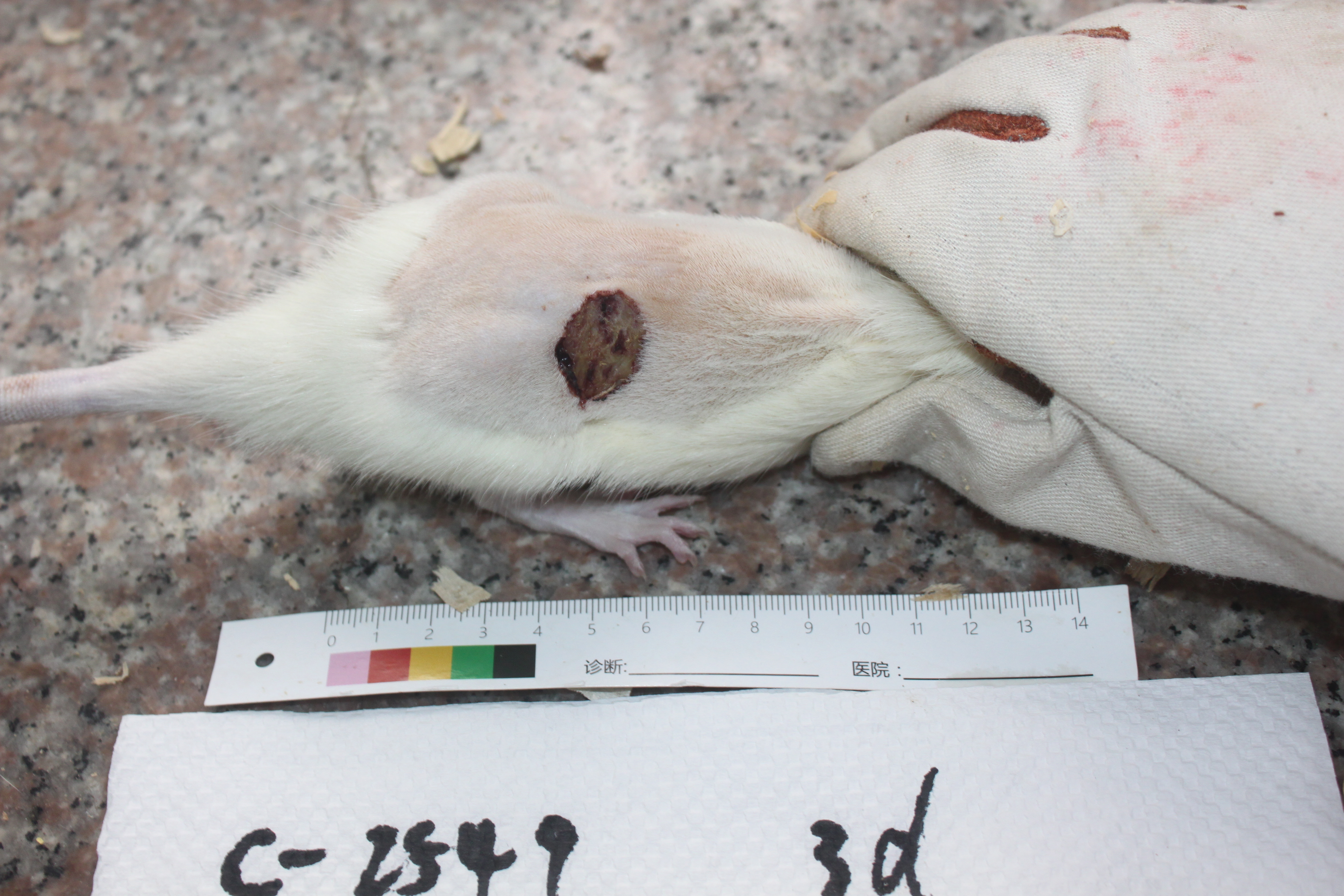

Supplement: S7 File — (ZIP) [file pone.0294566.s007.zip › support information/Wound healing rateú¿day 0 3 7ú⌐/day 3/sh-Control/Wound healing rate-day3-sh-Control (2).JPG]

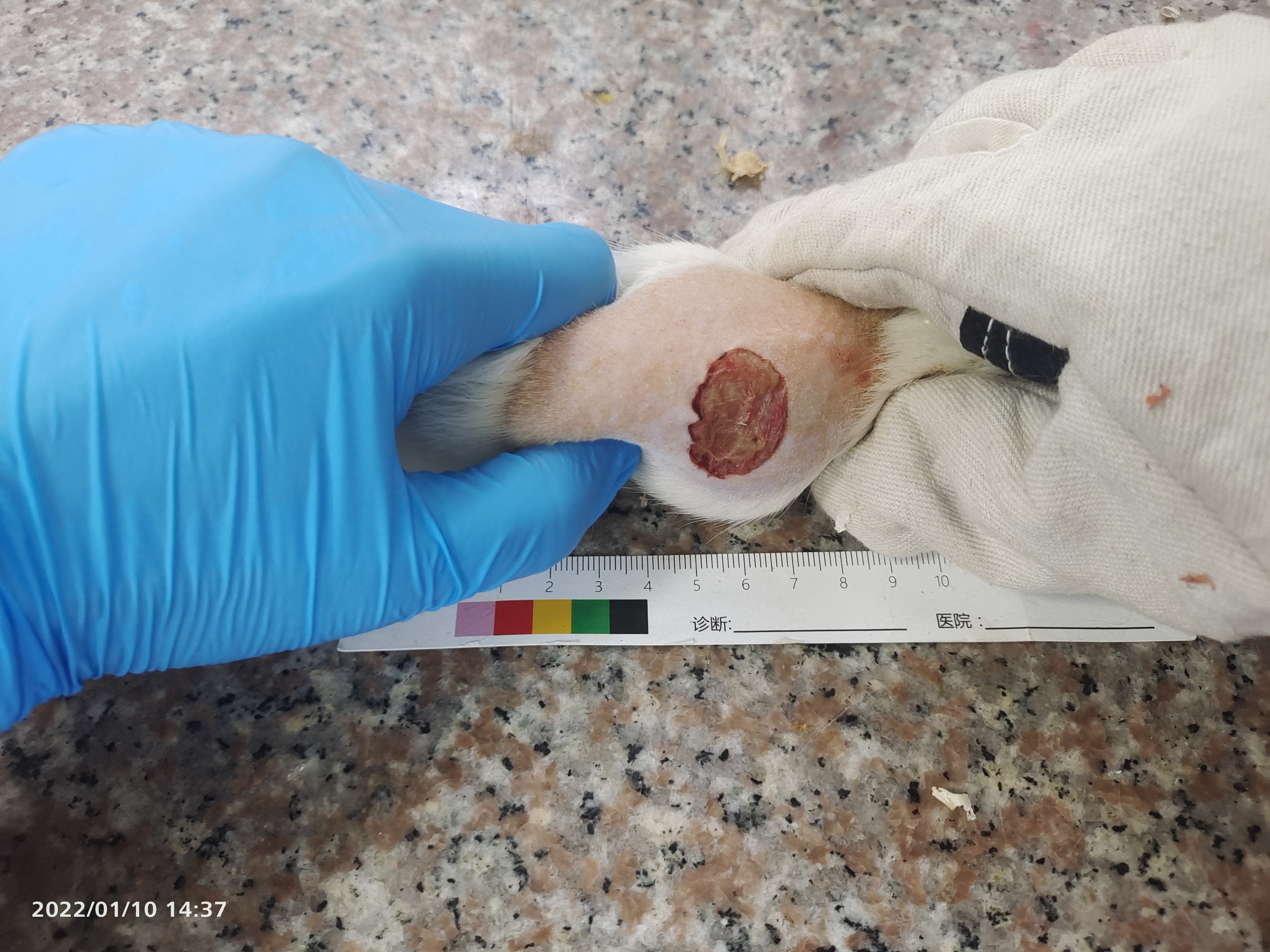

Supplement: S7 File — (ZIP) [file pone.0294566.s007.zip › support information/Wound healing rateú¿day 0 3 7ú⌐/day 3/sh-Control/Wound healing rate-day3-sh-Control (3).jpg]

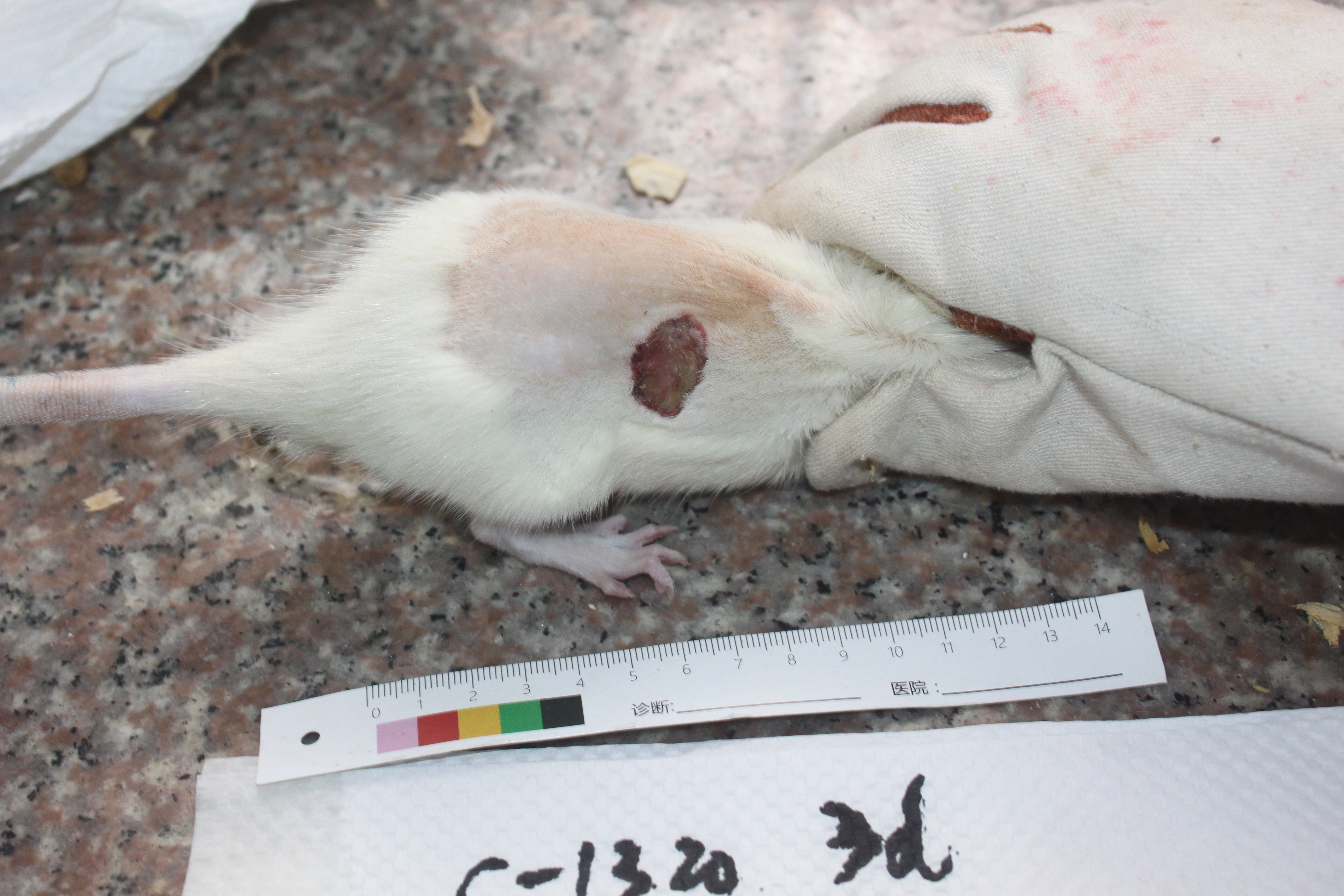

Supplement: S7 File — (ZIP) [file pone.0294566.s007.zip › support information/Wound healing rateú¿day 0 3 7ú⌐/day 3/sh-PHD2/Wound healing rate-day3-sh-PHD2 (1).JPG]

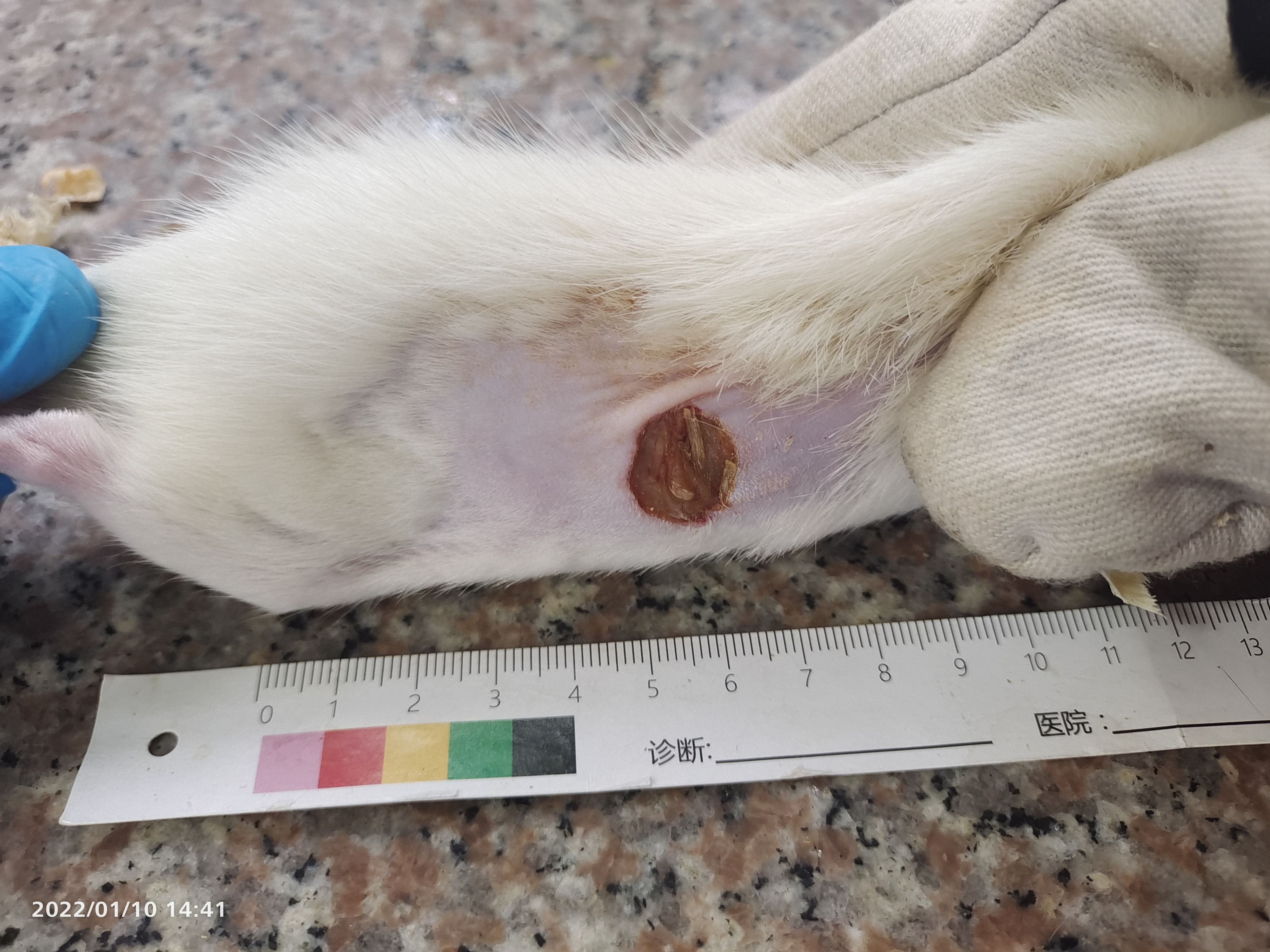

Supplement: S7 File — (ZIP) [file pone.0294566.s007.zip › support information/Wound healing rateú¿day 0 3 7ú⌐/day 3/sh-PHD2/Wound healing rate-day3-sh-PHD2 (2).jpg]

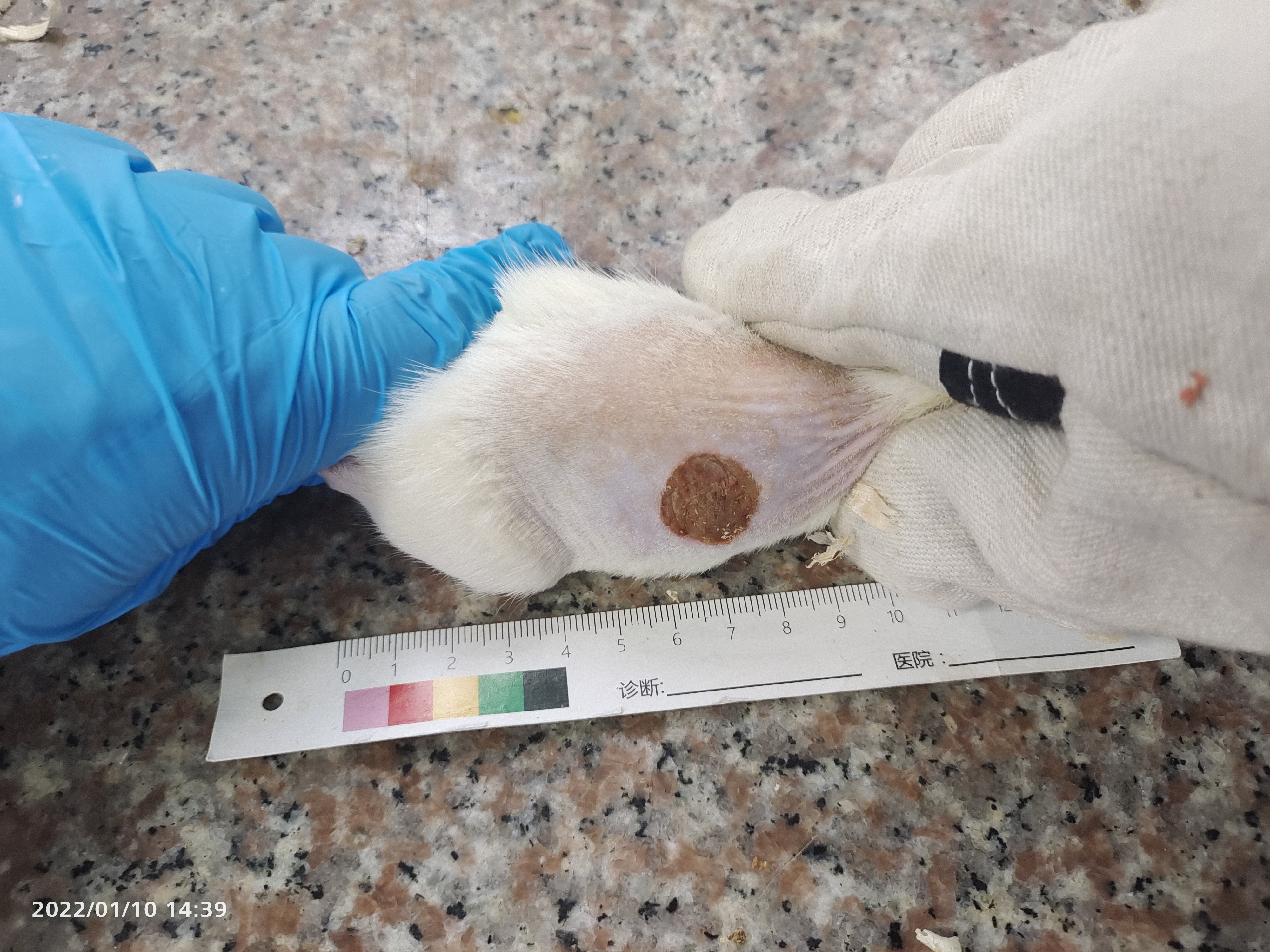

Supplement: S7 File — (ZIP) [file pone.0294566.s007.zip › support information/Wound healing rateú¿day 0 3 7ú⌐/day 3/sh-PHD2/Wound healing rate-day3-sh-PHD2 (3).jpg]

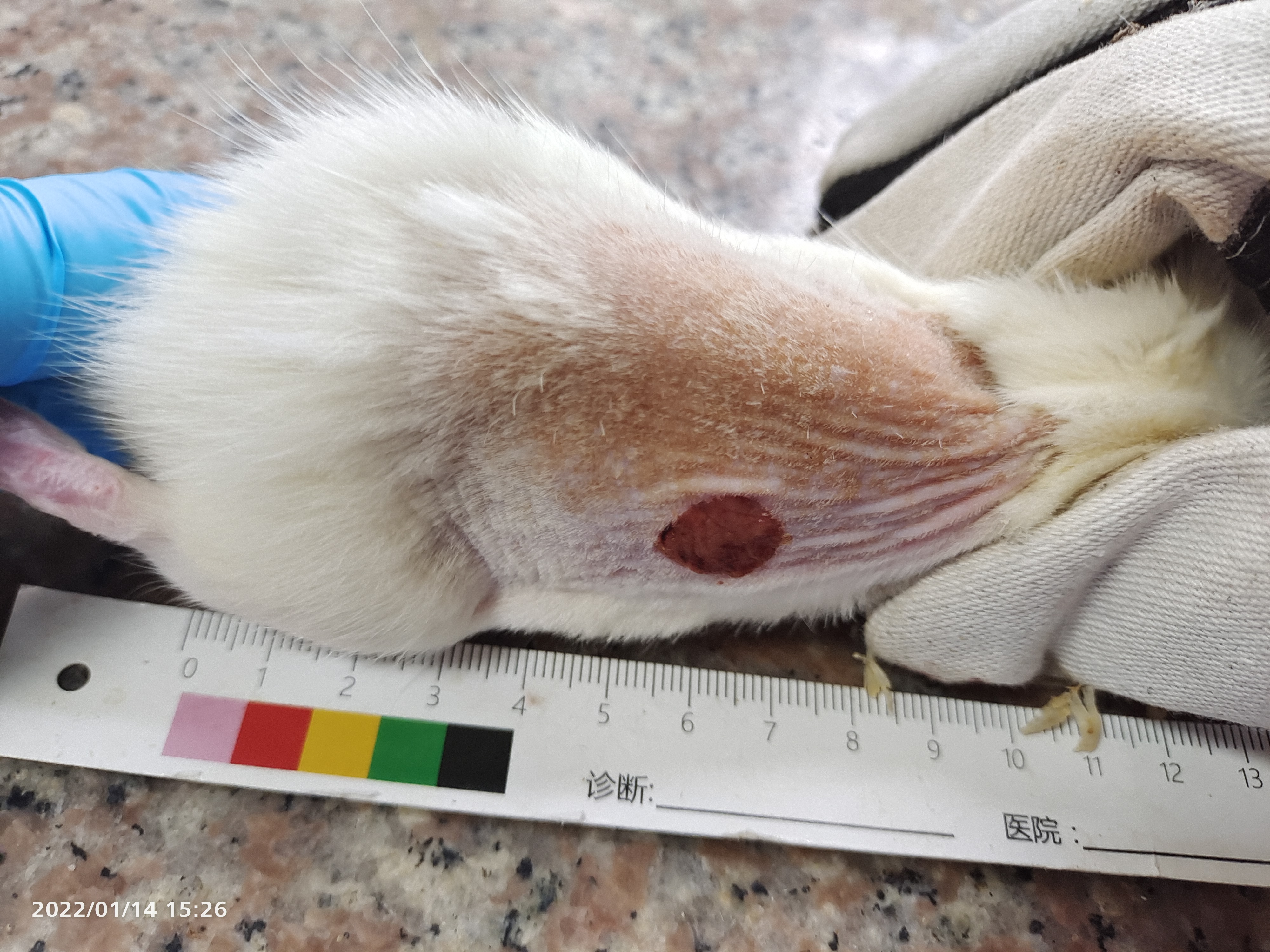

Supplement: S7 File — (ZIP) [file pone.0294566.s007.zip › support information/Wound healing rateú¿day 0 3 7ú⌐/day 7/sh-Control/Wound healing rate-day7-sh-Control (1).jpg]

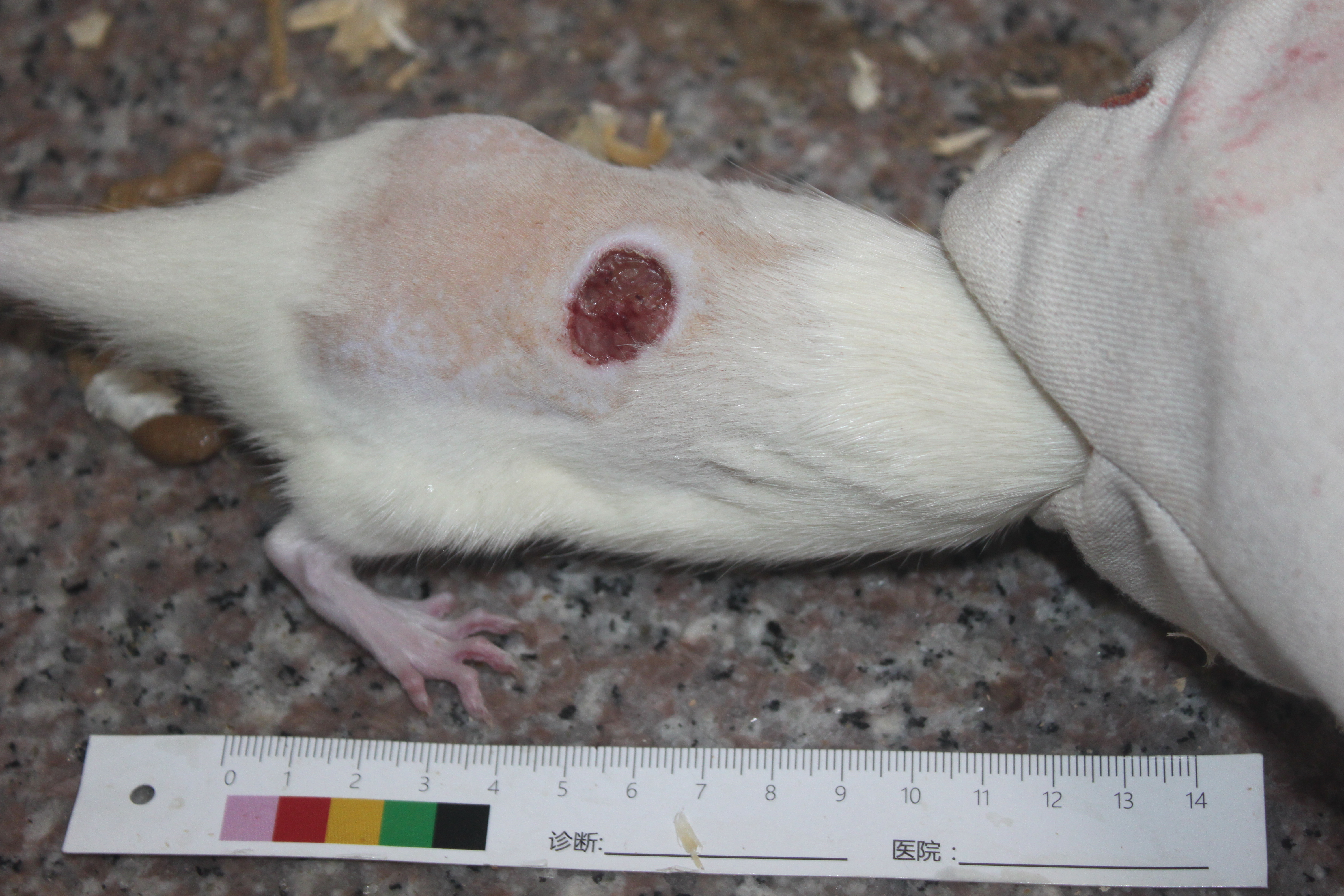

Supplement: S7 File — (ZIP) [file pone.0294566.s007.zip › support information/Wound healing rateú¿day 0 3 7ú⌐/day 7/sh-Control/Wound healing rate-day7-sh-Control (2).JPG]

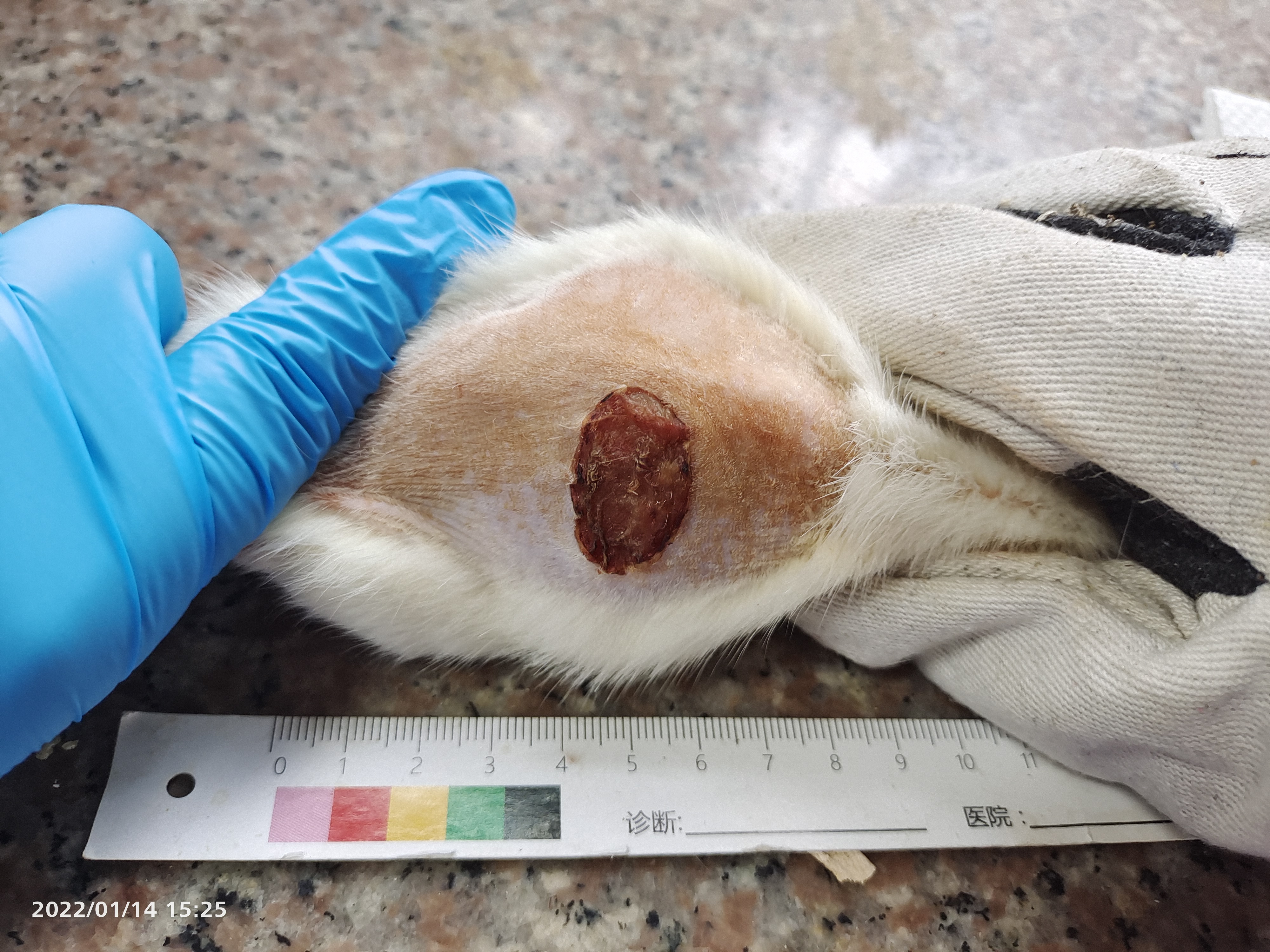

Supplement: S7 File — (ZIP) [file pone.0294566.s007.zip › support information/Wound healing rateú¿day 0 3 7ú⌐/day 7/sh-Control/Wound healing rate-day7-sh-Control (3).jpg]

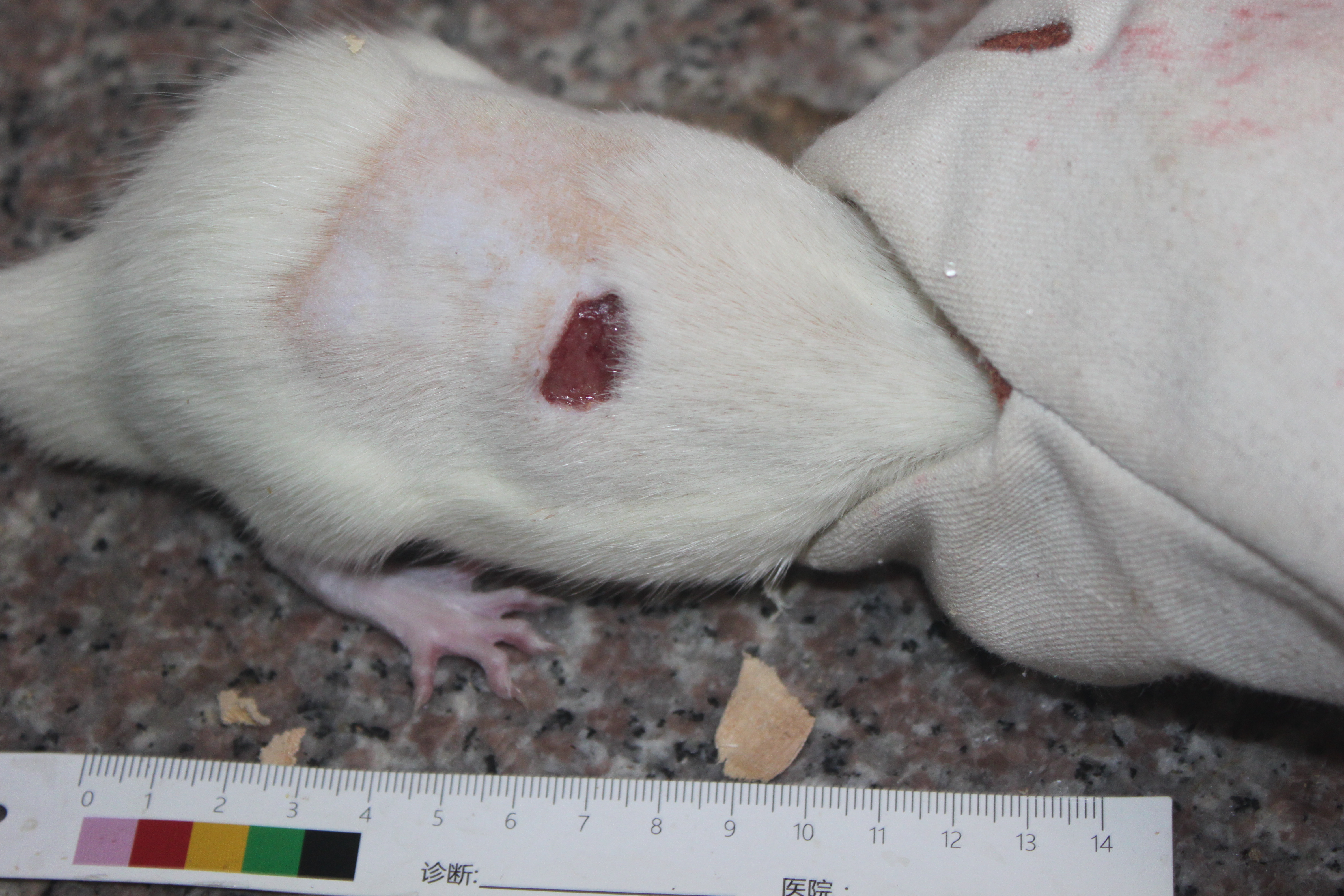

Supplement: S7 File — (ZIP) [file pone.0294566.s007.zip › support information/Wound healing rateú¿day 0 3 7ú⌐/day 7/sh-PHD2/Wound healing rate-day7-sh-PHD2 (1).JPG]
