# Supplementary figures and images for: Silencing of topical proline hydroxylase domain 2 promotes the healing of rat diabetic wounds by phosphorylating AMPK (part 2 of 2)
Source: PLoS One. 2023 Dec 1;18(12):e0294566. doi: 10.1371/journal.pone.0294566 (PMC10691724; doi:10.1371/journal.pone.0294566)

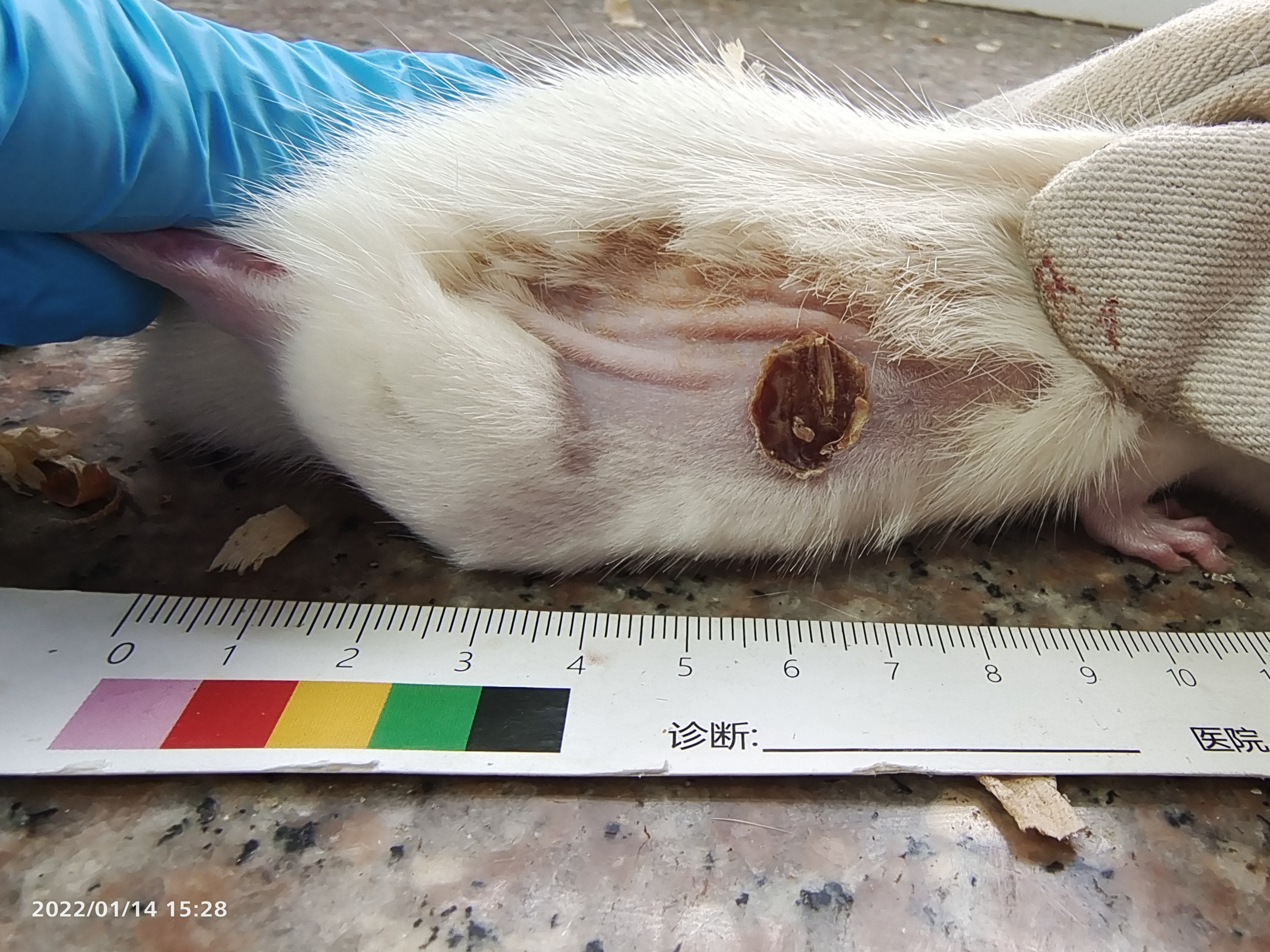

Supplement: S7 File — (ZIP) [file pone.0294566.s007.zip › support information/Wound healing rateú¿day 0 3 7ú⌐/day 7/sh-PHD2/Wound healing rate-day7-sh-PHD2 (2).jpg]

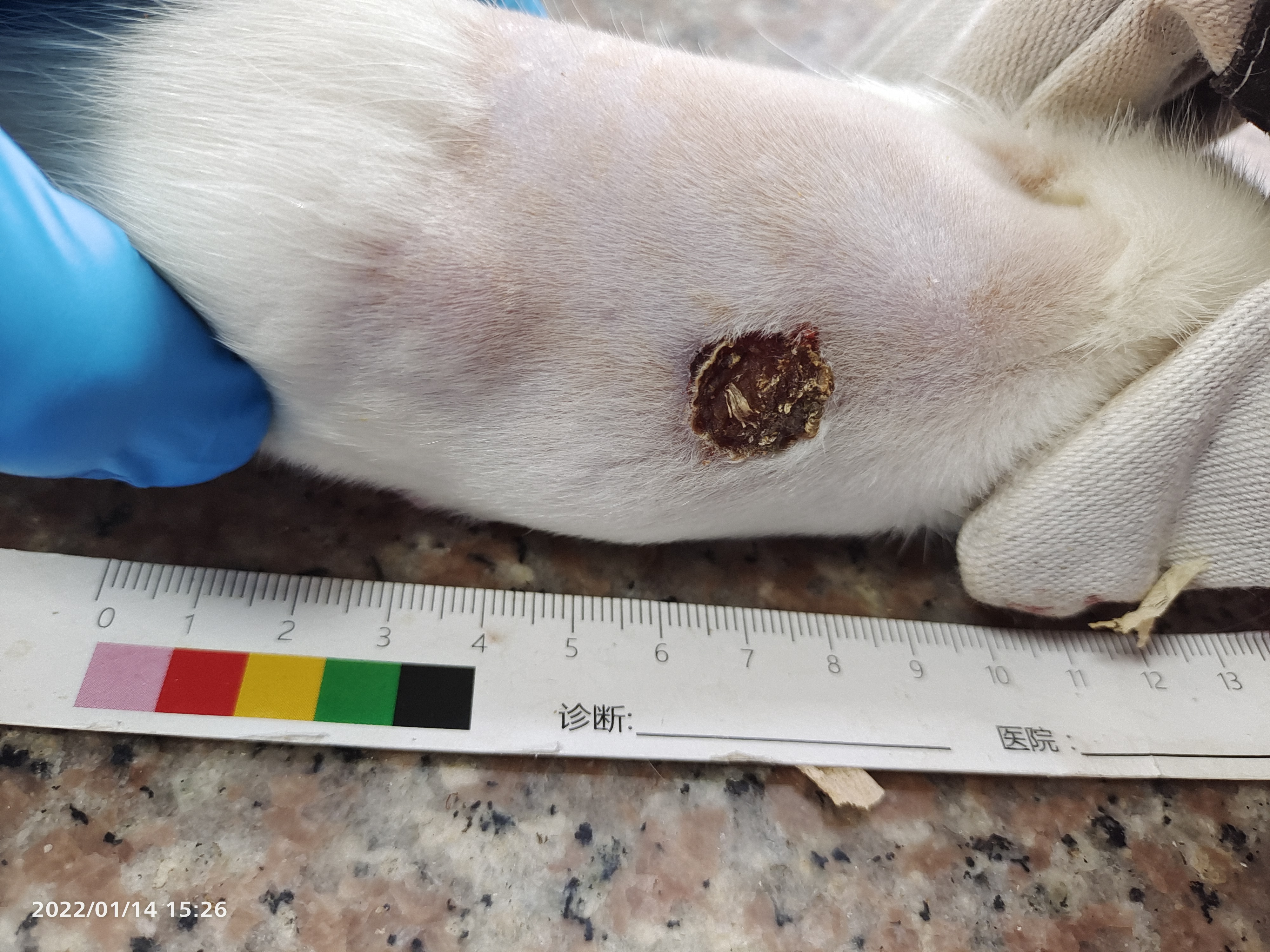

Supplement: S7 File — (ZIP) [file pone.0294566.s007.zip › support information/Wound healing rateú¿day 0 3 7ú⌐/day 7/sh-PHD2/Wound healing rate-day7-sh-PHD2 (3).jpg]

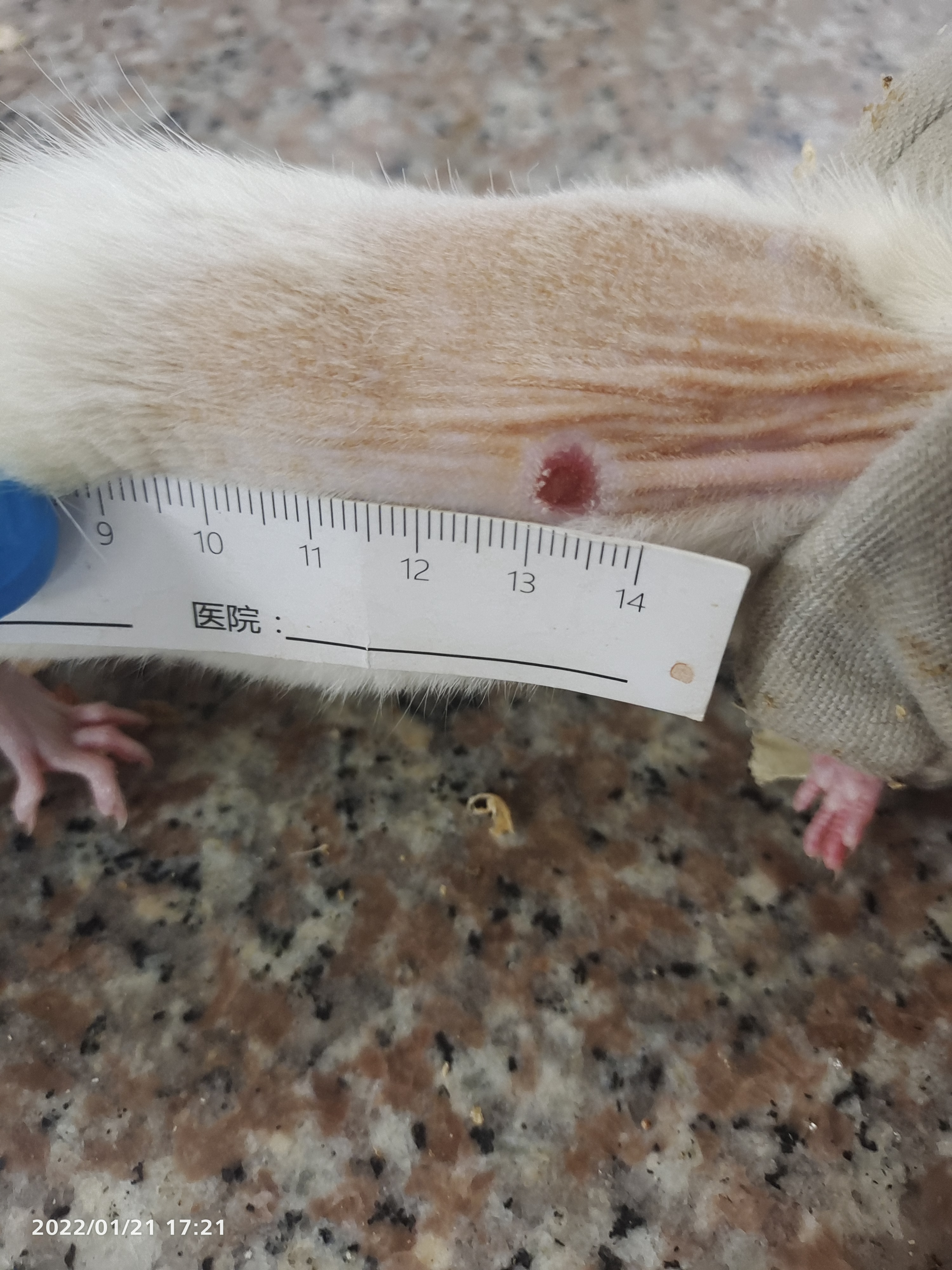

Supplement: S8 File — (ZIP) [file pone.0294566.s008.zip › support information/Wound healing rateú¿day 14 21ú⌐/day 14/sh-Control/Wound healing rate-day14-sh-Control (1).jpg]

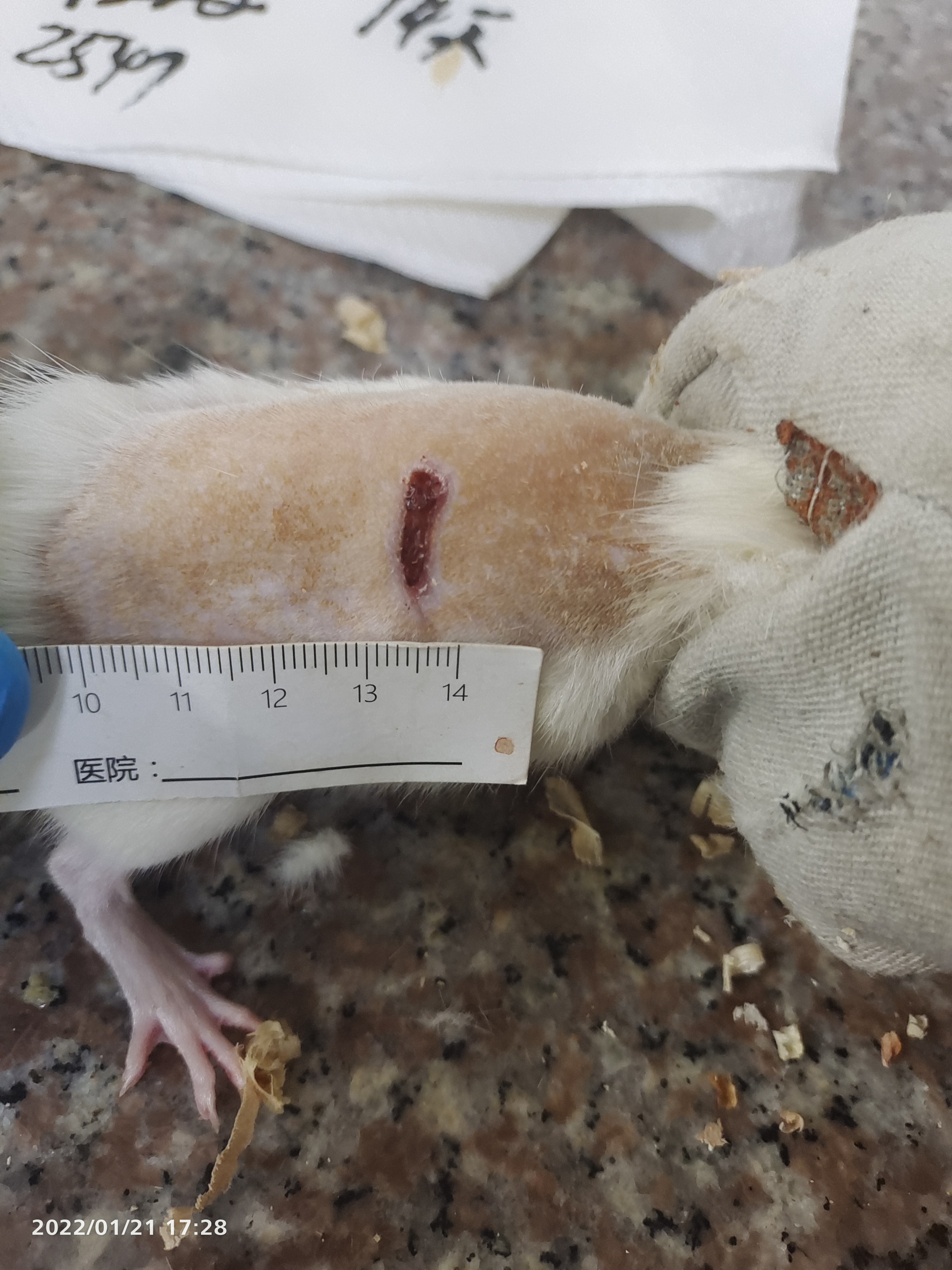

Supplement: S8 File — (ZIP) [file pone.0294566.s008.zip › support information/Wound healing rateú¿day 14 21ú⌐/day 14/sh-Control/Wound healing rate-day14-sh-Control (2).jpg]

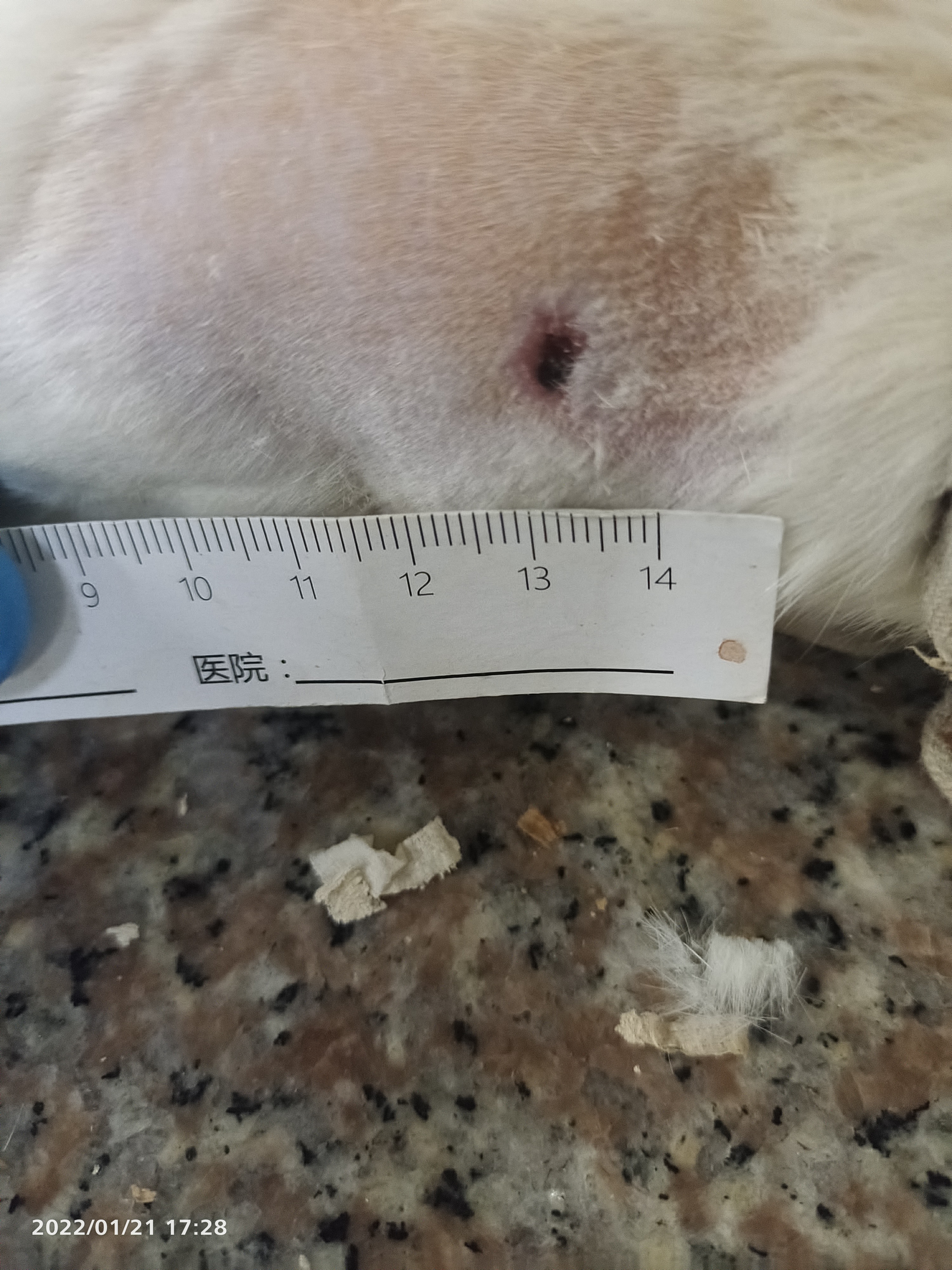

Supplement: S8 File — (ZIP) [file pone.0294566.s008.zip › support information/Wound healing rateú¿day 14 21ú⌐/day 14/sh-PHD2/Wound healing rate-day14-sh-PHD2 (1).jpg]

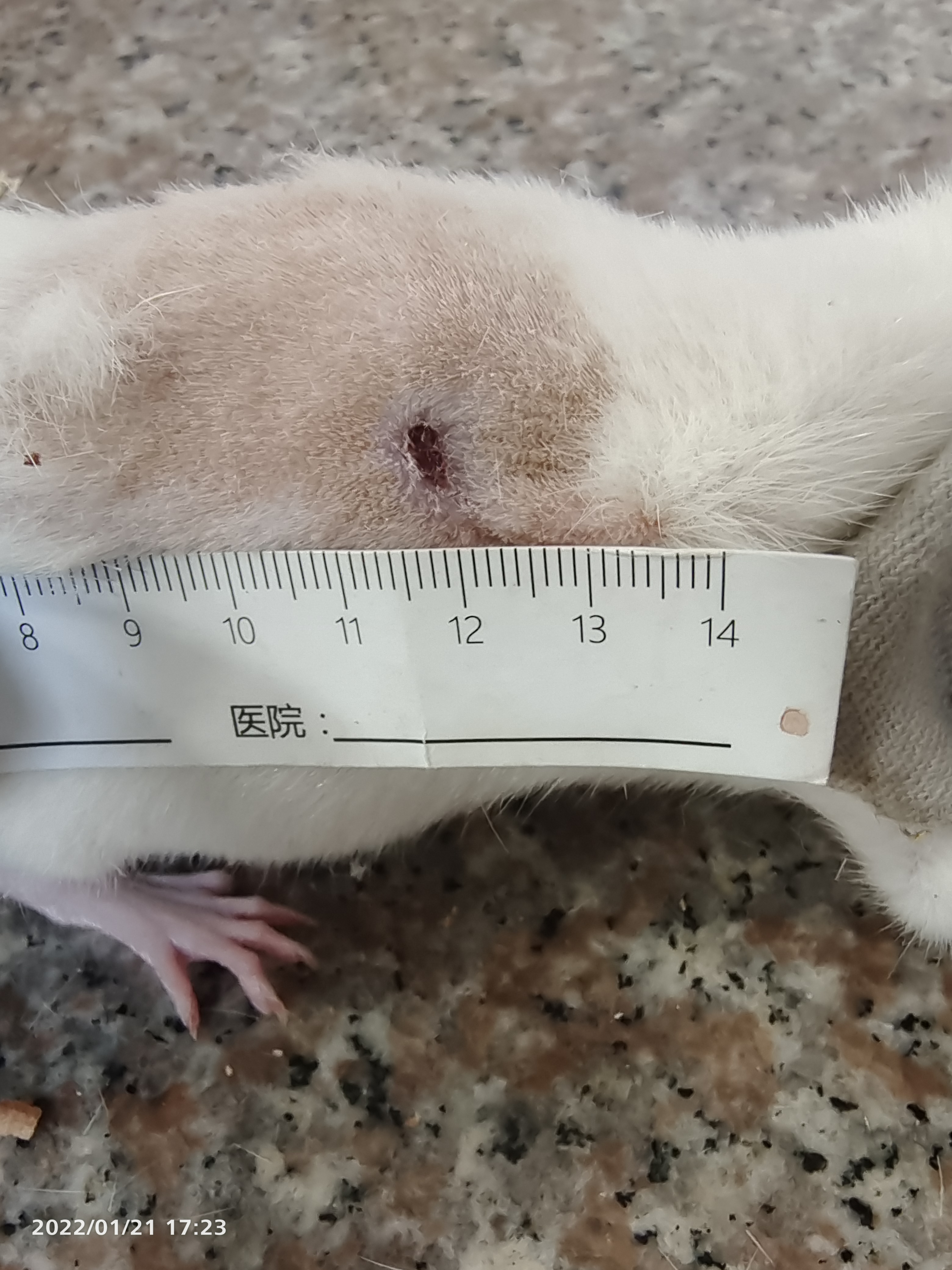

Supplement: S8 File — (ZIP) [file pone.0294566.s008.zip › support information/Wound healing rateú¿day 14 21ú⌐/day 14/sh-PHD2/Wound healing rate-day14-sh-PHD2 (2).jpg]

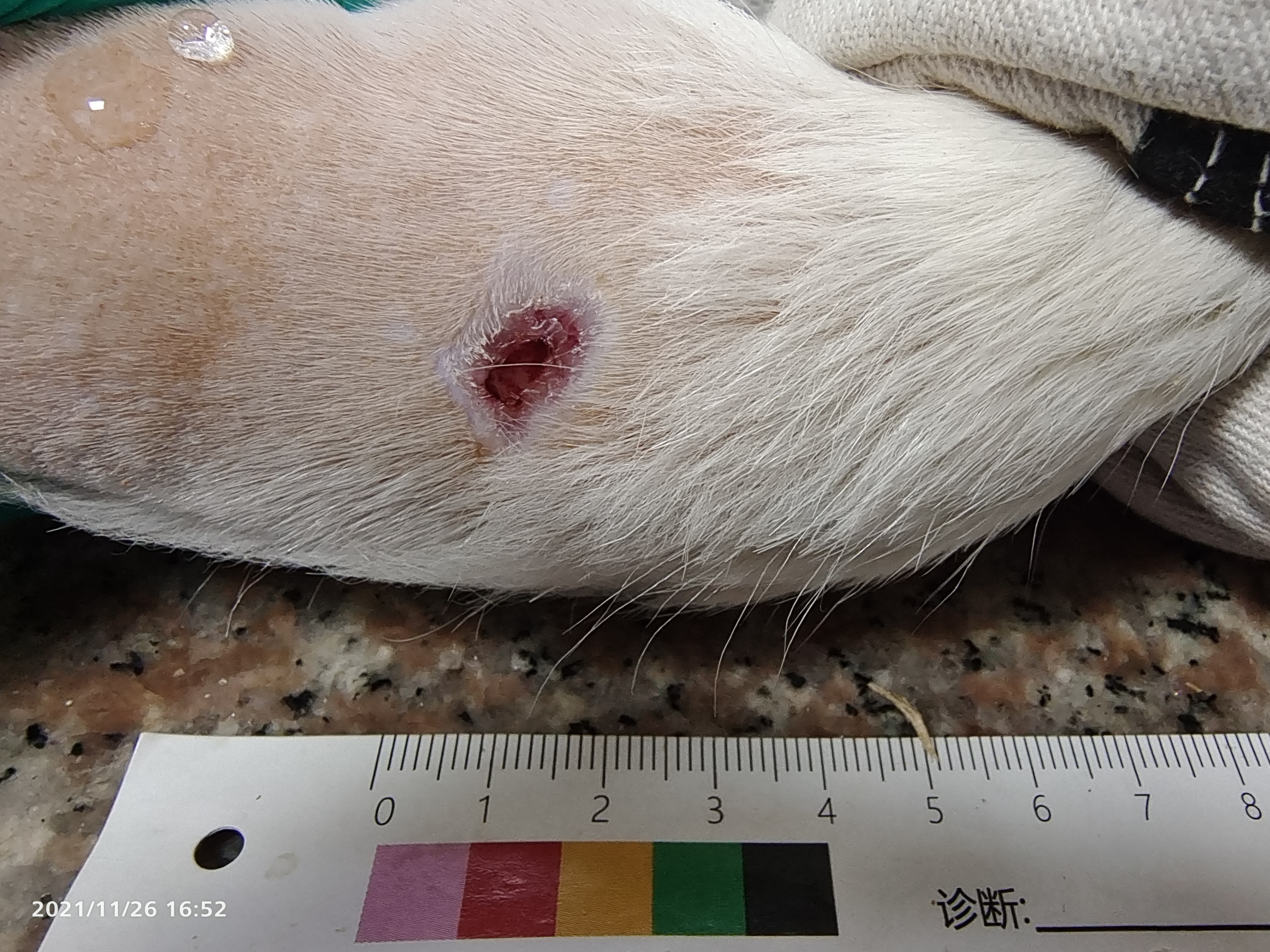

Supplement: S8 File — (ZIP) [file pone.0294566.s008.zip › support information/Wound healing rateú¿day 14 21ú⌐/day 21/sh-Control/Wound healing rate-day21-sh-Control (1).jpg]

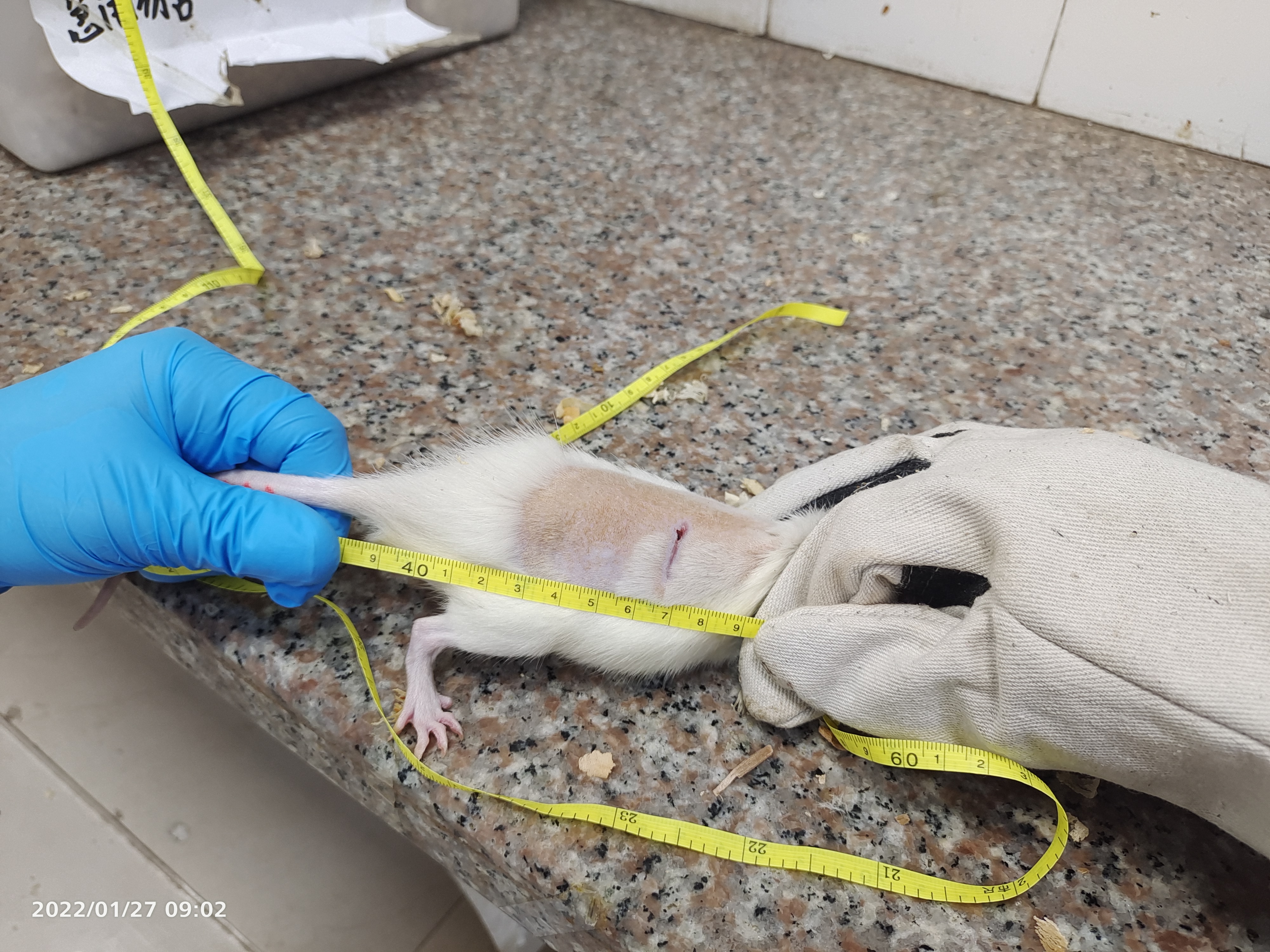

Supplement: S8 File — (ZIP) [file pone.0294566.s008.zip › support information/Wound healing rateú¿day 14 21ú⌐/day 21/sh-Control/Wound healing rate-day21-sh-Control (2).jpg]

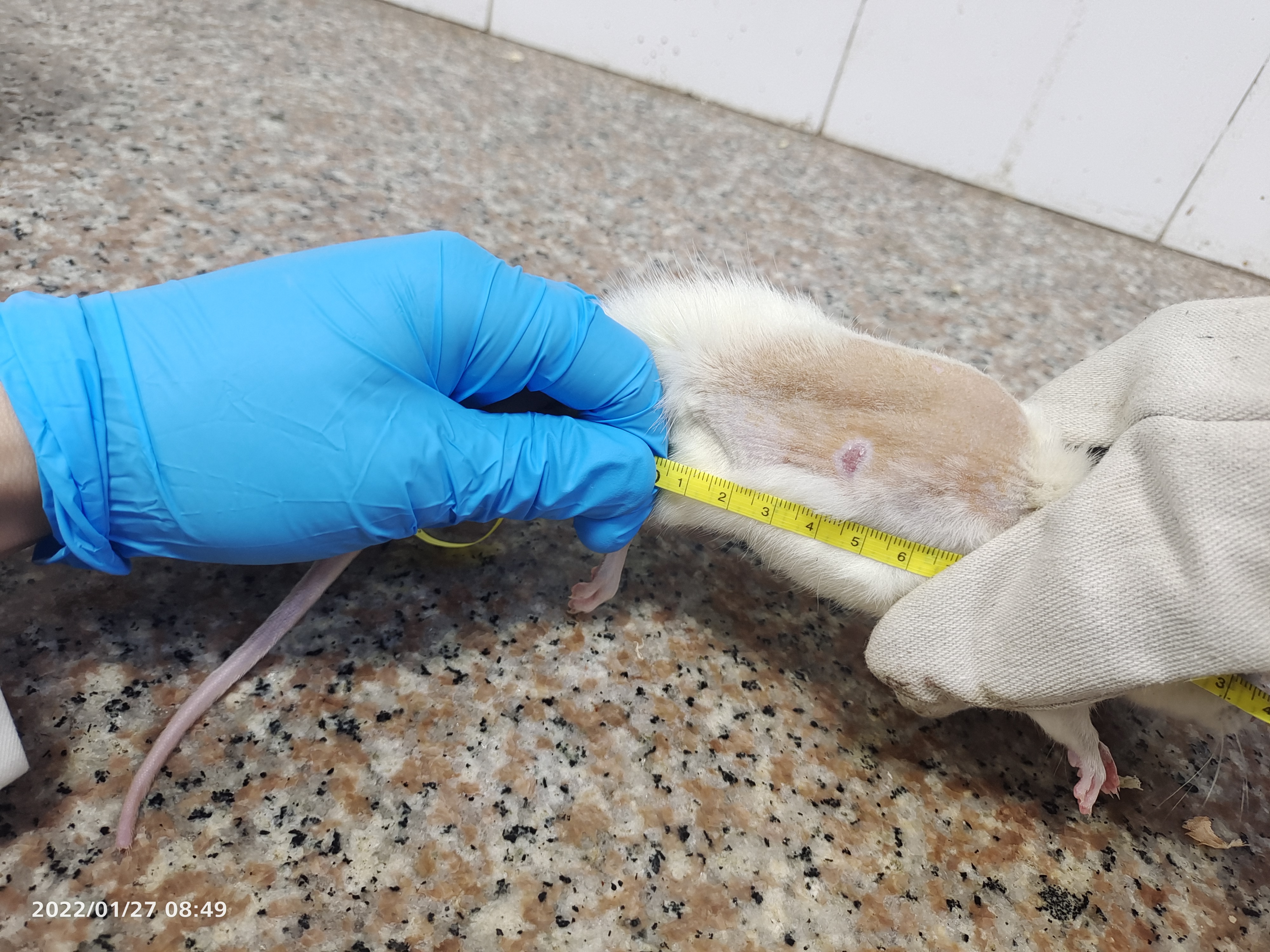

Supplement: S8 File — (ZIP) [file pone.0294566.s008.zip › support information/Wound healing rateú¿day 14 21ú⌐/day 21/sh-Control/Wound healing rate-day21-sh-Control (3).jpg]

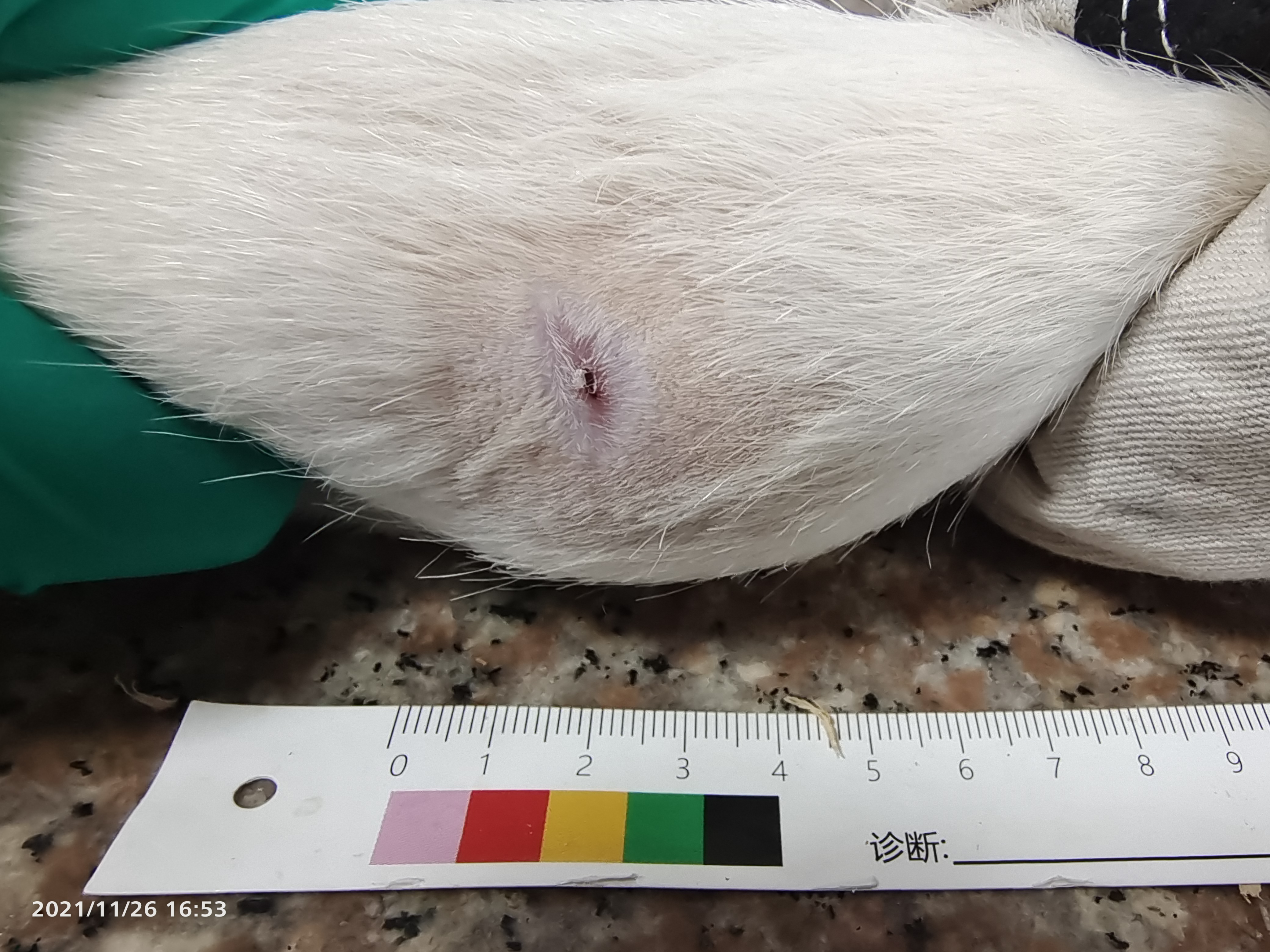

Supplement: S8 File — (ZIP) [file pone.0294566.s008.zip › support information/Wound healing rateú¿day 14 21ú⌐/day 21/sh-PHD2/Wound healing rate-day21-sh-PHD2 (1).jpg]

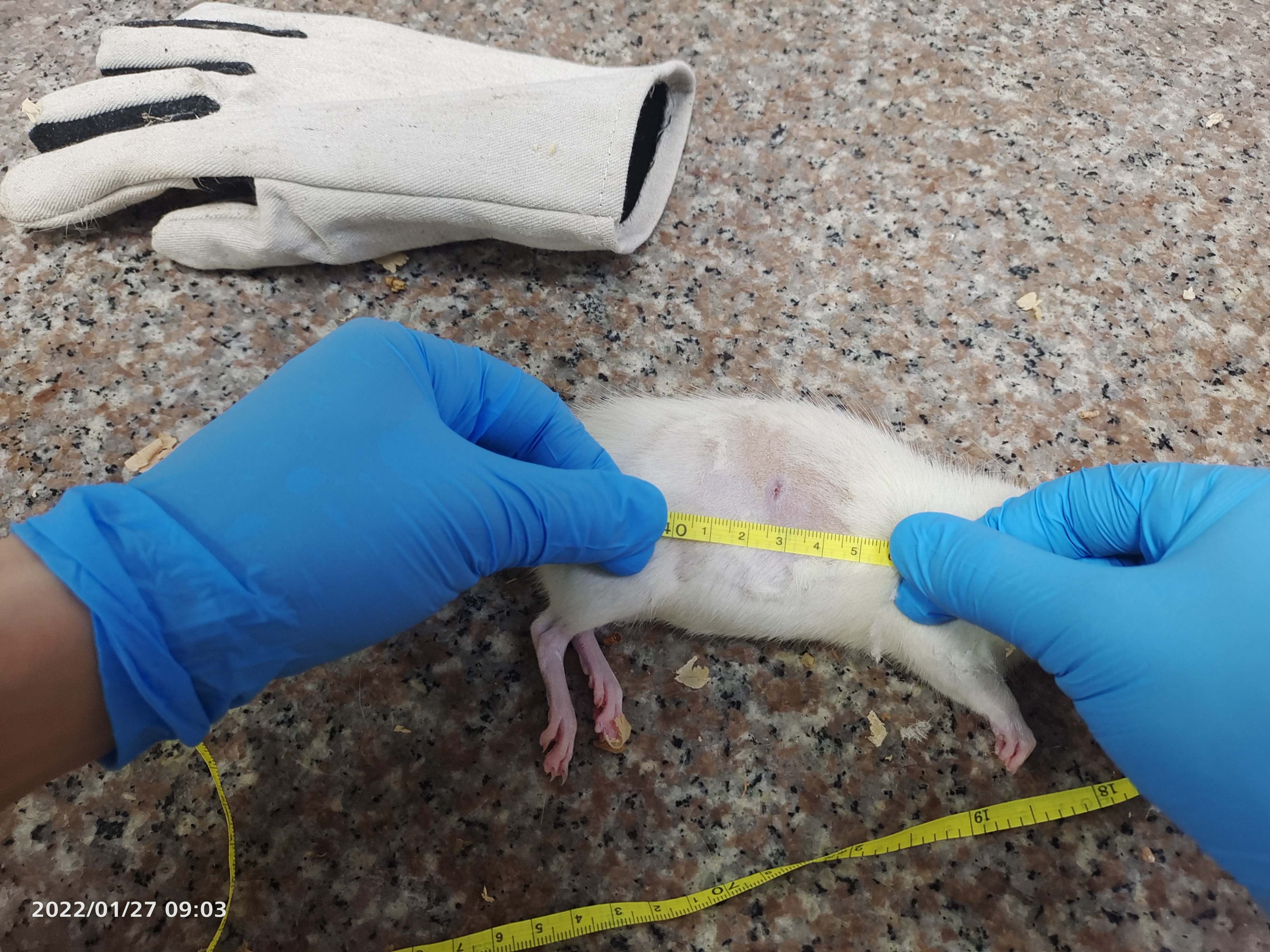

Supplement: S8 File — (ZIP) [file pone.0294566.s008.zip › support information/Wound healing rateú¿day 14 21ú⌐/day 21/sh-PHD2/Wound healing rate-day21-sh-PHD2 (2).jpg]

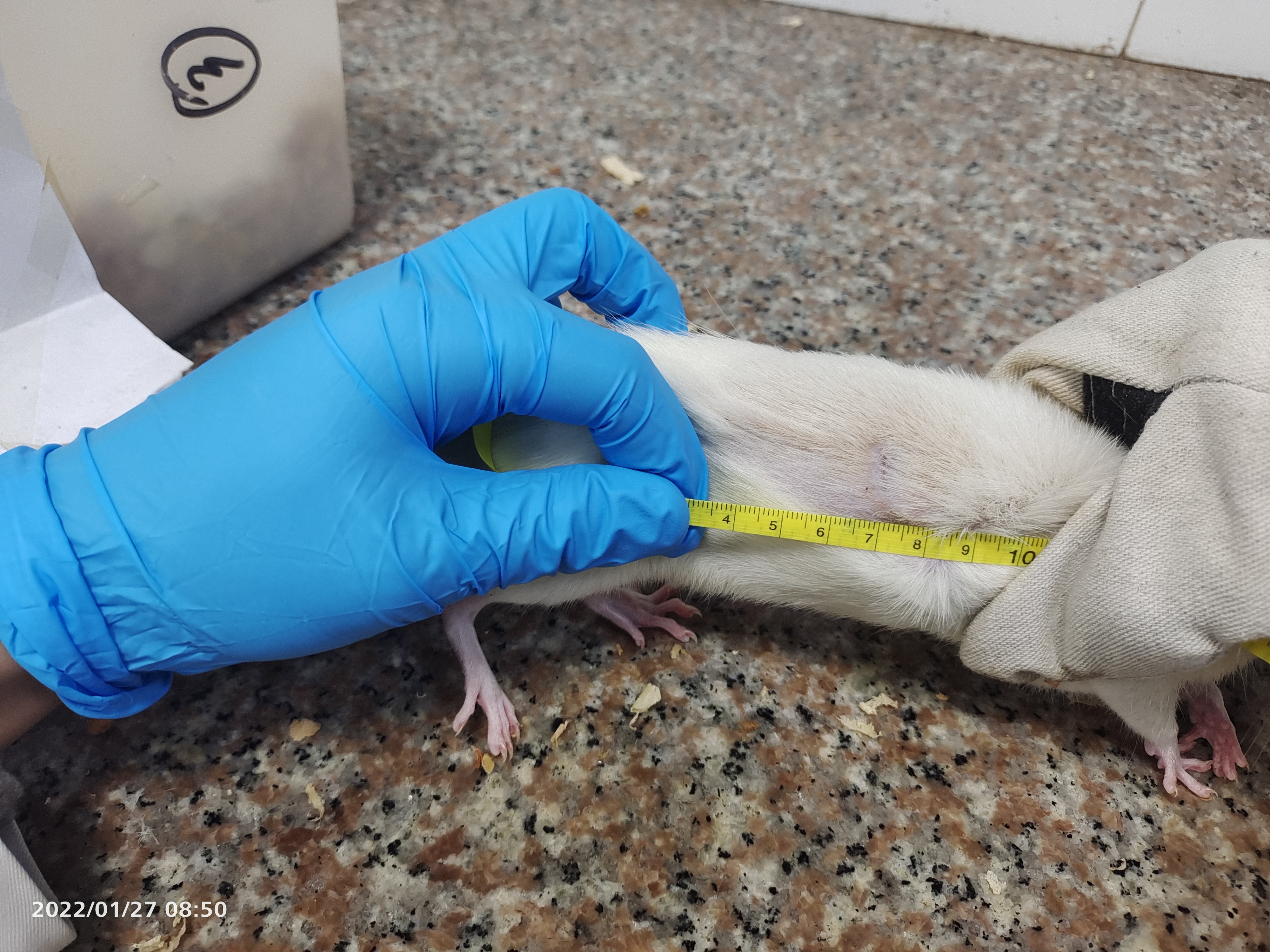

Supplement: S8 File — (ZIP) [file pone.0294566.s008.zip › support information/Wound healing rateú¿day 14 21ú⌐/day 21/sh-PHD2/Wound healing rate-day21-sh-PHD2 (3).jpg]

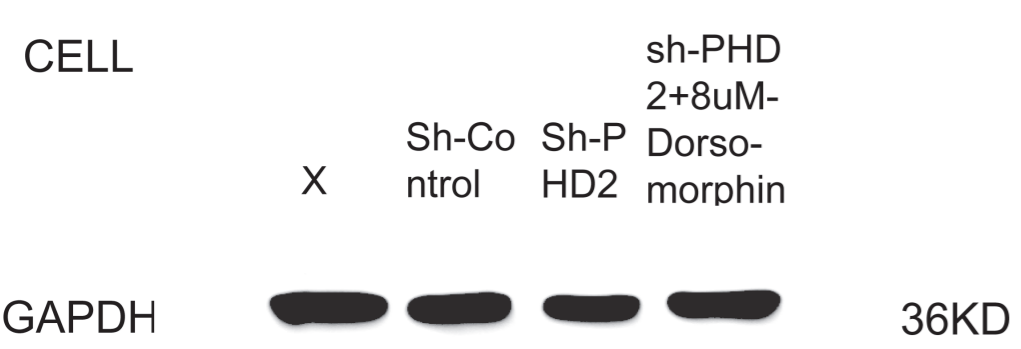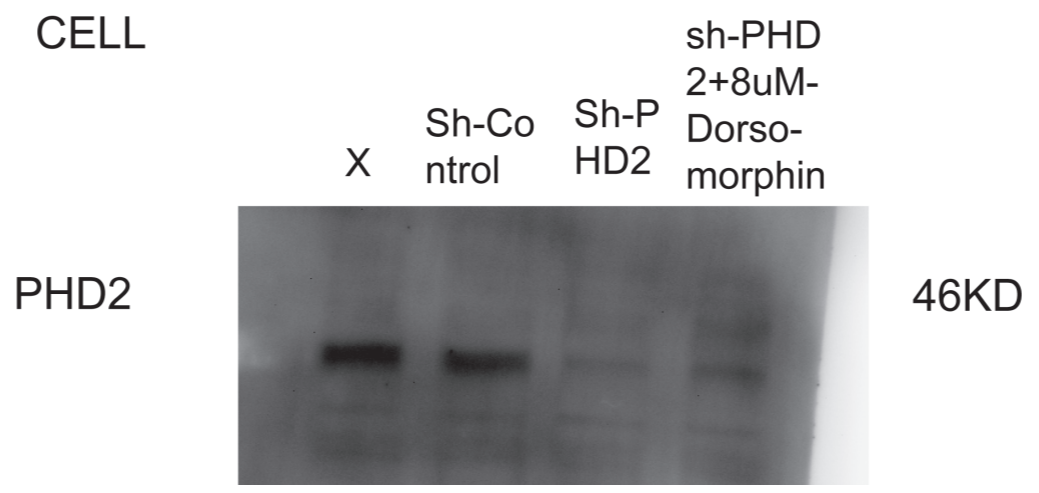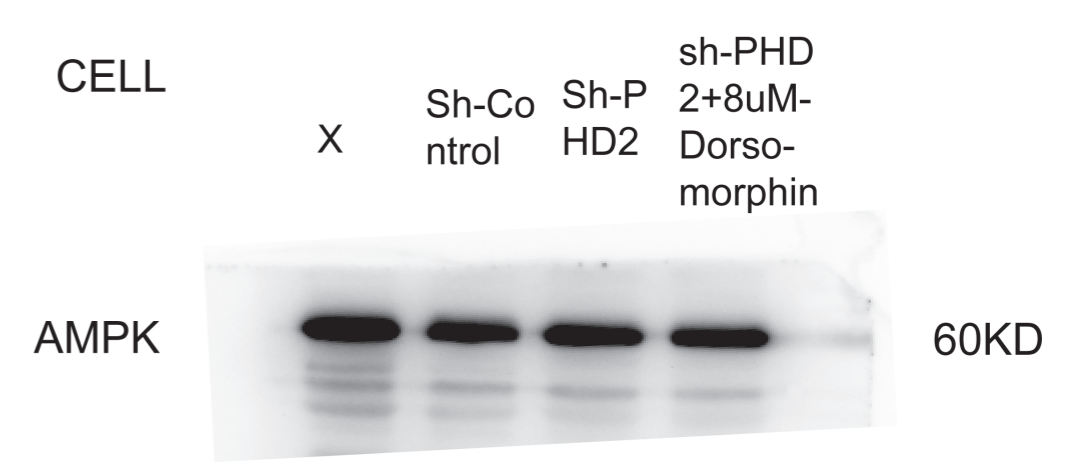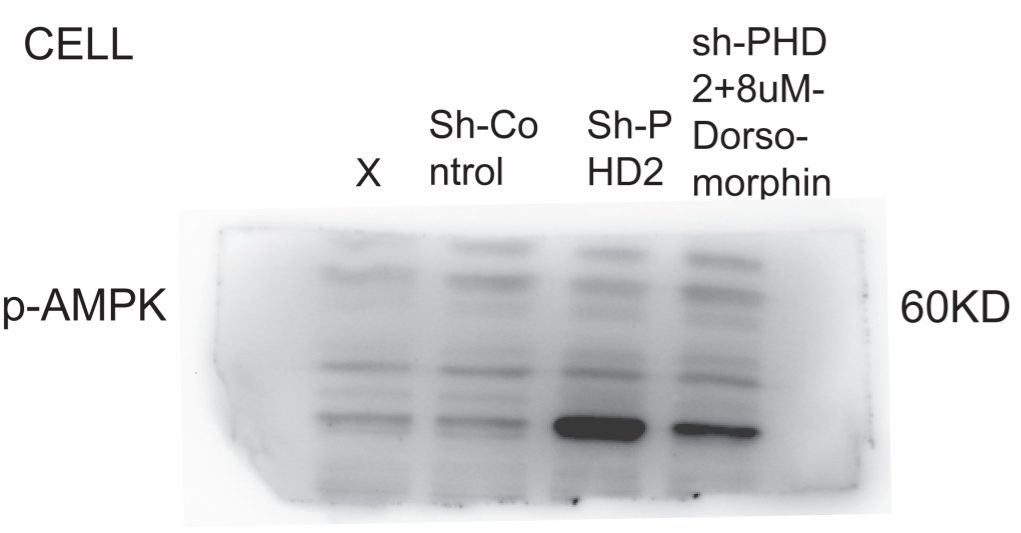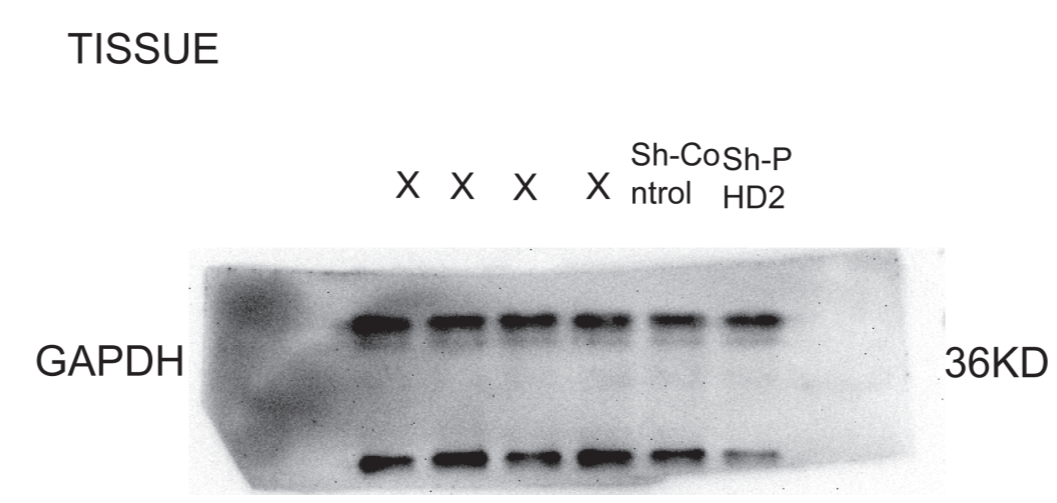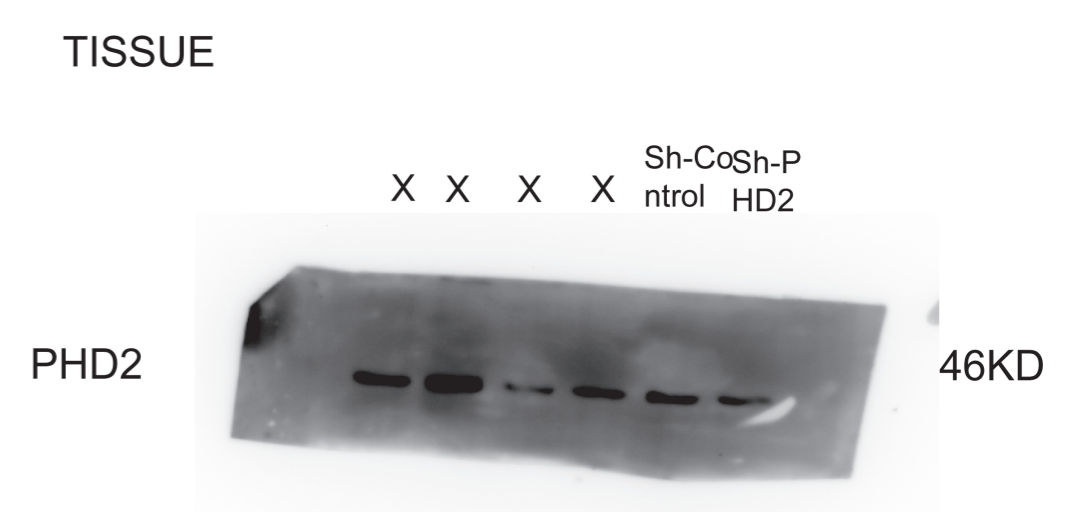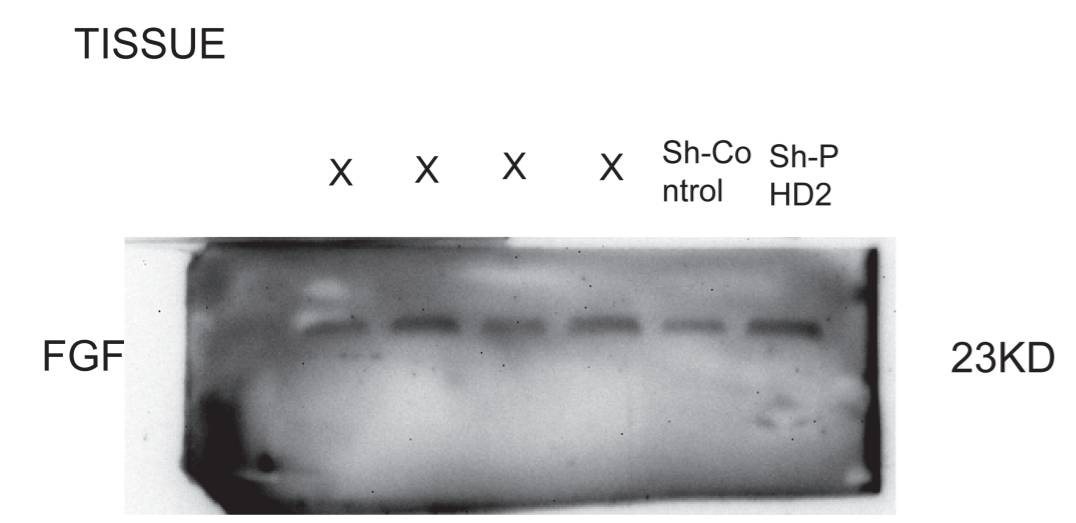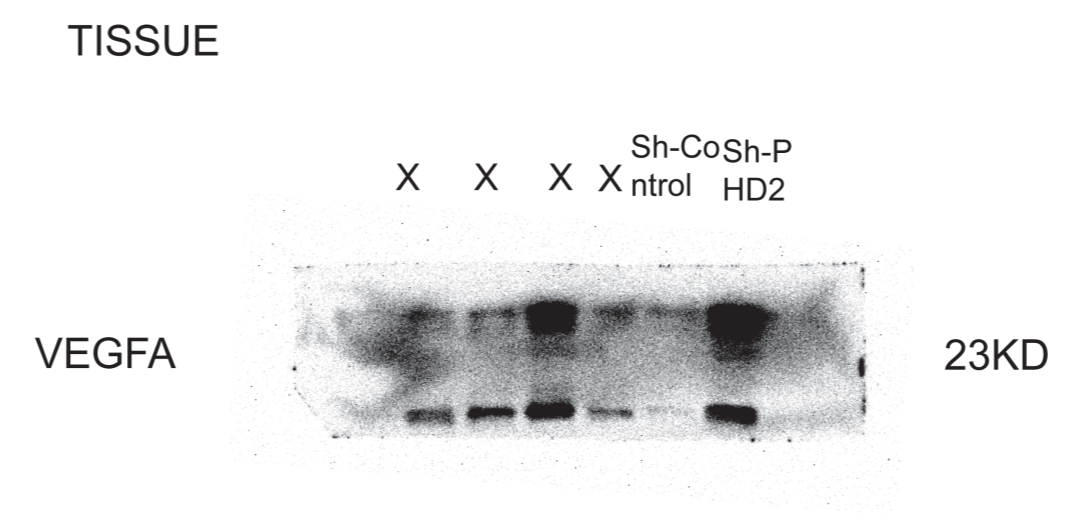

Supplement: S1 Raw images — (PDF) [file pone.0294566.s009.pdf]
