# Supplementary material for: Silencing of topical proline hydroxylase domain 2 promotes the healing of rat diabetic wounds by phosphorylating AMPK
Source: PLoS One. 2023 Dec 1;18(12):e0294566. doi: 10.1371/journal.pone.0294566 (PMC10691724; doi:10.1371/journal.pone.0294566)
Supplement: Attachment — Submitted filename: Paper.pdf [file pone.0294566.s010.pdf]

Title: **Silencing of topical proline hydroxylase domain 2 promotes the healing of rat diabetic wounds by phosphorylating AMPK**

Authors: Defu Xie, Mengchang Liu，Yingxi Lin，Xingke Liu，Hong Yan*

In the present study, the authors demonstrate the critical role of the PHD2 pathway in the hypoxic signaling cascade. Hypoxic signaling activates the AMPK system by directly phosphorylating the PHD2 pathway, leading to a significant endogenous hypoxic protective effect. The study's objective and rationale are well-described, and the authors provide a clear explanation of the methods used. The results obtained are significant.

Here are a few minor suggestions for improvement:

1. The abbreviation "DMEM" is not explained on page 4, line 86.
2. The abbreviation "XF" is not explained on page 6, line 126.
3. In the References section, the word "References" should be written before listing the references.
4. In reference number 7, the journal name is written in all capital letters. It should be formatted similarly to the other references.
5. In Figure 4, the labels for "sh-PHD2" and "sh-PDH2+8μM Dorsomorphin" are not written properly in the histogram.
