# Supplementary material for: Silencing of topical proline hydroxylase domain 2 promotes the healing of rat diabetic wounds by phosphorylating AMPK
Source: PLoS One. 2023 Dec 1;18(12):e0294566. doi: 10.1371/journal.pone.0294566 (PMC10691724; doi:10.1371/journal.pone.0294566)
Supplement: Attachment — Submitted filename: Response to Reviewers.docx [file pone.0294566.s011.docx]

Dear Editor, Dear reviewers,

Thank you for your letter dated September 1. We were pleased to know that our work was rated as potentially acceptable for publication in Journal, subject to adequate revision. We thank the reviewers for the time and effort that they have put into reviewing the previous version of the manuscript. Their suggestions have enabled us to improve our work. Based on the instructions provided in your letter, we uploaded the file of the revised manuscript. Accordingly, we have uploaded a copy of the original manuscript with all the changes highlighted by using the track changes mode in Word. Appended to this letter is our point-by-point response to the comments raised by the reviewers. The comments are reproduced and our responses are given directly afterward in a different color (red). We would like also to thank you for allowing us to resubmit a revised copy of the manuscript.

We hope that the revised manuscript is accepted for publication in“PLOS ONE” .

Sincerely, Hong Yan

Responses to journals:

1. Please ensure that your manuscript meets PLOS ONE's style requirements, including those for file naming. The PLOS ONE style templates can be found at

https://journals.plos.org/plosone/s/file?id=wjVg/PLOSOne_formatting_sample_main_body.pdf and <https://journals.plos.org/plosone/s/file?id=ba62/PLOSOne_formatting_sample_title_authors_affiliations.pdf>

Responses:We have revised the manuscript according to your template standard when we started to submit the manuscript, if there is any deficiency, we hope that you can clearly point it out, and we will make changes to it as soon as possible.

2. To comply with PLOS ONE submissions requirements, in your Methods section, please provide additional information regarding the experiments involving animals and ensure you have included details on (1) methods of sacrifice, (2) efforts to alleviate suffering.

Responses:We have added methods of sacrifice and efforts to alleviate sufferingon page 4, line 85 of the manuscript. if there are any deficiencies, we hope that you will let us know and we will perfect them at the first opportunity.

3. Thank you for stating the following financial disclosure:

"Tthe authors who received the award is Mr Yan . The full name of the funder is (Luzhou-Medical University) Cooperation Project,the grant number is No. 2019LZXNYDZ08.The URL of funder website is http://luzhou.tccxfw.com .The sponsor was involved in the design of this study."

Please state what role the funders took in the study. If the funders had no role, please state: "The funders had no role in study design, data collection and analysis, decision to publish, or preparation of the manuscript."

If this statement is not correct you must amend it as needed.

Please include this amended Role of Funder statement in your cover letter; we will change the online submission form on your behalf.

Responses:For contributions made by funders, we have added information in Cover Latter.

4. In your Data Availability statement, you have not specified where the minimal data set underlying the results described in your manuscript can be found. PLOS defines a study's minimal data set as the underlying data used to reach the conclusions drawn in the manuscript and any additional data required to replicate the reported study findings in their entirety. All PLOS journals require that the minimal data set be made fully available. For more information about our data policy, please see http://journals.plos.org/plosone/s/data-availability.

"Upon re-submitting your revised manuscript, please upload your study’s minimal underlying data set as either Supporting Information files or to a stable, public repository and include the relevant URLs, DOIs, or accession numbers within your revised cover letter. For a list of acceptable repositories, please see http://journals.plos.org/plosone/s/data-availability#loc-recommended-repositories. Any potentially identifying patient information must be fully anonymized.

Important: If there are ethical or legal restrictions to sharing your data publicly, please explain these restrictions in detail. Please see our guidelines for more information on what we consider unacceptable restrictions to publicly sharing data: http://journals.plos.org/plosone/s/data-availability#loc-unacceptable-data-access-restrictions. Note that it is not acceptable for the authors to be the sole named individuals responsible for ensuring data access.

We will update your Data Availability statement to reflect the information you provide in your cover letter.

Responses:We have uploaded the minimum dataset as support information.

5. PLOS ONE now requires that authors provide the original uncropped and unadjusted images underlying all blot or gel results reported in a submission’s figures or Supporting Information files. This policy and the journal’s other requirements for blot/gel reporting and figure preparation are described in detail at https://journals.plos.org/plosone/s/figures#loc-blot-and-gel-reporting-requirements and https://journals.plos.org/plosone/s/figures#loc-preparing-figures-from-image-files. When you submit your revised manuscript, please ensure that your figures adhere fully to these guidelines and provide the original underlying images for all blot or gel data reported in your submission. See the following link for instructions on providing the original image data: https://journals.plos.org/plosone/s/figures#loc-original-images-for-blots-and-gels.

In your cover letter, please note whether your blot/gel image data are in Supporting Information or posted at a public data repository, provide the repository URL if relevant, and provide specific details as to which raw blot/gel images, if any, are not available. Email us at plosone@plos.org if you have any questions.

Responses:We have uploaded all gel results as support information.

6. Please upload a copy of Supporting Information Figure/Table/etc. Fig.1-7 which you refer to in your text on page 22.

Responses:We have uploaded all the information corresponding to Fig.1-7 as supporting information.

7. Please review your reference list to ensure that it is complete and correct. If you have cited papers that have been retracted, please include the rationale for doing so in the manuscript text, or remove these references and replace them with relevant current references. Any changes to the reference list should be mentioned in the rebuttal letter that accompanies your revised manuscript. If you need to cite a retracted article, indicate the article’s retracted status in the References list and also include a citation and full reference for the retraction notice.

Responses:In the subsequent discussion, we have added further information on the impact of altered PHD2 on other relevant pathways, and therefore cited three additional papers, references 37, 38, and 39.All references are complete and correct, and all references are formatted and inserted in the format required by the journals.

Responses to reviewer 1:

1、The abbreviation "DMEM" is not explained on page 4, line 86.

Responses:We have added to the description of "DMEM" on page 4, line 86 of the manuscript, with the following explanation: Dulbecco's Modified Eagle Medium Thank you for your suggestion!

2、The abbreviation "XF" is not explained on page 6, line 126.

Responses:We have explained "XF" on page 6, line 126 of the manuscript, specifically: Extrancellular Flux .Thank you for your suggestion!

3、In the References section, the word "References" should be written before listing the references.

Responses:We've added this detail before the reference, thanks for the suggestion!

4、In reference number 7, the journal name is written in all capital letters. It should be formatted similarly to the other references.

Responses:We have re-corrected the formatting of all references as required by the journal, thank you for your suggestion!

5、In Figure 4, the labels for "sh-PHD2" and "sh-PDH2+8μM Dorsomorphin" are not written properly in the histogram.

Responses:As Figure 4 is not standardised enough, we have recreated Figure 4 and re-uploaded it, thank you for your suggestion.

Responses to reviewer 2:

1. To achieve effective suppression of PHD2, authors should possess a comprehensive understanding of transcriptomics through thorough and high-throughput study.

Responses:Due to the time problem, we can't improve the PHD2 related high throughput research for the time being, in the future our group will continue to improve the related research for PHD2, thank you for your suggestion!

2. The authors should emphasize the distinct pathways that undergo changes due to sh-PHD2. These pathways should be elaborated upon in greater detail within the discussion section.

Responses:On page 16, line 350 of the manuscript, we provide additional elaboration in response to the other pathways that are altered due to sh-PHD2, and thank you for the suggestion!

3. The authors should verify that the figures in the panel maintain an optimal resolution for the imaging of Figure 3, Figure 5, and Figure 6.

Responses:When uploading Figure 3, Figure 5, and Figure 6., we are sure that it is already the best resolution, we are not sure why the resolution has dropped in the finished manuscript, we will upload all the figures again, if the result is still not good, we hope that you can contact us, thank you for your suggestion!

4. Authors should ensure that the gene names should be written in italics.

Responses:We have gone through the manuscript again and changed all gene names to italics, specifically: manuscript page 8, line 174; manuscript page 17, line 360.
